# Systems Approach to Discovery of Therapeutic Targets for Vein Graft Disease: PPARα Pivotally Regulates Metabolism, Activation, and Heterogeneity of Macrophages and Lesion Development

**DOI:** 10.1161/CIRCULATIONAHA.119.043724

**Published:** 2021-04-06

**Authors:** Julius L. Decano, Sasha A. Singh, Cauê Gasparotto Bueno, Lang Ho Lee, Arda Halu, Sarvesh Chelvanambi, Joan T. Matamalas, Hengmin Zhang, Andrew K. Mlynarchik, Jiao Qiao, Amitabh Sharma, Shin Mukai, Jianguo Wang, Daniel G. Anderson, C. Keith Ozaki, Peter Libby, Elena Aikawa, Masanori Aikawa

**Affiliations:** 1 Center for Interdisciplinary Cardiovascular Sciences, Cardiovascular Division (J.L.D., S.A.S., C.G.B., L.H.L., A.H., S.C., J.T.M., H.Z., A.K.M., J.Q., A.S., S.M., J.W., E.A., M.A.), Brigham and Women’s Hospital, Harvard Medical School, Boston, MA.; 2 Channing Division of Network Medicine (A.H., A.S., M.A.), Brigham and Women’s Hospital, Harvard Medical School, Boston, MA.; 3 Department of Medicine, Division of Vascular and Endovascular Surgery, Department of Surgery (C.K.O.), Brigham and Women’s Hospital, Harvard Medical School, Boston, MA.; 4 Center for Excellence in Vascular Biology (P.L., E.A., M.A.), Brigham and Women’s Hospital, Harvard Medical School, Boston, MA.; 5 Institutes for Medical Engineering and Science, Massachusetts Institute of Technology, Cambridge (D.G.A.).; 6 Department of Human Pathology, I.M. Sechenov First Moscow State Medical University of the Ministry of Health, Russia (E.A., M.A.).

**Keywords:** arteriovenous, fistula, inflammation, macrophage, pemafibrate, proteomics, systems biology, vein graft

## Abstract

Vein graft failure remains a common clinical challenge. We applied a systems approach in mouse experiments to discover therapeutic targets for vein graft failure.

**Methods:**

Global proteomics and high-dimensional clustering on multiple vein graft tissues were used to identify potential pathogenic mechanisms. The PPARs (peroxisome proliferator-activated receptors) pathway served as an example to substantiate our discovery platform. In vivo mouse experiments with macrophage-targeted PPARα small interfering RNA, or the novel, selective activator pemafibrate demonstrate the role of PPARα in the development and inflammation of vein graft lesions. In vitro experiments further included metabolomic profiling, quantitative polymerase chain reaction, flow cytometry, metabolic assays, and single-cell RNA sequencing on primary human and mouse macrophages.

**Results:**

We identified changes in the vein graft proteome associated with immune responses, lipid metabolism regulated by the PPARs, fatty acid metabolism, matrix remodeling, and hematopoietic cell mobilization. PPARα agonism by pemafibrate retarded the development and inflammation of vein graft lesions in mice, whereas gene silencing worsened plaque formation. Pemafibrate also suppressed arteriovenous fistula lesion development. Metabolomics/lipidomics, functional metabolic assays, and single-cell analysis of cultured human macrophages revealed that PPARα modulates macrophage glycolysis, citrate metabolism, mitochondrial membrane sphingolipid metabolism, and heterogeneity.

**Conclusions:**

This study explored potential drivers of vein graft inflammation and identified PPARα as a novel potential pharmacological treatment for this unmet medical need.

## Abstract 2

Supplemental Digital Content is available in the text.

**Clinical Perspective**

**What Is New?**

- Using proteomics, network analysis, and high-resolution ultrasonography in the experimental vein graft disease model, we established a discovery platform to identify novel therapeutic targets.
- Peroxisome proliferator-activated receptor α activation suppresses the development of vein graft and arteriovenous fistula lesions.
- Peroxisome proliferator-activated receptor α reduces macrophage activation by influencing macrophage heterogeneity, mitochondrial integrity, and metabolome.

**What Are the Clinical Implications?**

- Peripheral artery disease and chronic kidney disease prevalences are increasing, warranting a need for vein grafts and arteriovenous fistula.
- Vein graft and arteriovenous fistula failure lack effective therapeutic options. Our target discovery platform is applicable to such diseases.

**Editorial, see p 2471**

Peripheral artery disease (PAD) remains a global health burden that affects 200 million people worldwide.^1^ Recent advances in pharmacotherapy have reduced major adverse limb events in patients with PAD.^2^ However, only surgical treatment of severe limb ischemia caused by the occlusive PAD using autologous vein grafts or prosthetic graft bypass can salvage jeopardized limbs.^3^ Autologous vein bypass has proven superior to prosthetic grafts.^4,5^ However, 25% to 45% of autologous vein grafts for PAD narrow or impede in the initial postoperative year.^6–8^ Saphenous vein–sourced coronary artery bypass grafts also have initial-year failure rates of 10% to 15%.^9^ Contributors to vein graft failure include poor distal runoff, a progression of distal arterial disease, anastomotic hyperplasia, and technical issues. Vein grafts develop mural lesions that share histological features with arterial atherosclerosis, including accumulation of macrophage foam cells.^10^ Previous reports have demonstrated signs of plaque rupture of inflamed vein grafts.^11,12^

Greater understanding of the cellular and molecular mechanisms that underlie vein graft failure could address this unmet medical need. Such challenges have driven efforts to seek innovative approaches in identifying underlying mechanisms. This study used unbiased omics and systems biology to gain insight into the pathogenesis of vein graft disease. This undertaking identified various candidate pathways. Among them, we chose the peroxisome proliferator-activated receptor α (PPARα) pathway to substantiate our target discovery platform by examining whether this pathway indeed participates in the pathogenesis of vein graft disease. The results show that PPARα regulates macrophage metabolism, inflammatory activation, and vein graft lesion development, which merits evaluation for the medical therapy of vein graft disease.

## Methods

Detailed methods are provided in the Data Supplement. We will make the data and methods used to conduct the research available to any researcher on request.

All animal experiments conformed to institutional guidelines. Low-density lipoprotein receptor–deficient *Ldlr*^−/−^ mice were used for vein graft and arteriovenous fistula (AVF) surgery. Loss-of-function and gain-of-function studies used in vivo silencing by PPARα small interfering RNA (siRNA) encapsulated in macrophage-targeted lipid nanoparticles and the PPARα-selective activator pemafibrate, respectively. Blind randomization of control and treatment groups was carried out, and assignments remained undisclosed until after completion of the analysis.

Liquid chromatography–tandem mass spectrometry for proteomics used the Thermo LTQ-Orbitrap. The data were normalized by the protein median area under the curve,^13,14^ with protein abundance trends, analyzed using Qlucore Omics Explorer 3.2, R (version 3.3.2) and our original software, XINA (multiplexed isobaric mass tagging-based kinetics data for network analysis).^15^ All data were queried against the mouse UniProt database via Proteome Discoverer Package (version 1.4, Thermo Fisher Scientific). MetaCore was used for pathway enrichment. Pathway enrichment, network building, and topology analysis were done using MetaCore, Python scripts-Networkx package, Cytoscape, and Gephi.

Human peripheral blood mononuclear cells were derived from the buffy coats. Single-cell quantitative polymerase chain reaction was done using C1+HD BioMark (Fluidigm). Single-cell RNA sequencing was done on the 10x Genomics platform. Cell hashing (Totalseq, Biolegend) was performed to minimize sample batch-to-batch variation. After cDNA library sequencing (Novagene), raw data were analyzed using Cell Ranger, Loupe, SeqGeq, MetaCore, Plotly, and Excel. Metabolomics and lipidomics were outsourced (Metabolon). A mitochondrial stress test was performed using Seahorse Bioanalyzer. Mitochondrial integrity was tested using tetramethylrhodamine methyl ester perchlorate (TMRM) (ThermoFisher) staining. Mitochondrial membrane damage by reactive oxygen species was detected by MitoSox staining (Thermo Fisher Scientific). Flow cytometry was done on a BD Aria LSR II SORP.

### Statistical Analysis

Two-group comparisons between tests and control groups were made using a *t* test after assessing their normality distribution (GraphPad). Multigroup comparison in proteomics was made using ANOVA filtering for *P* values and false discovery rates <0.05 (Qlucore Omics Explorer). Spearman correlation assessed a 2-parameter interrelationship. Time course proteomics was evaluated using XINA, an R package that provides a statistical workflow to investigate the trend clusters and coabundance patterns of proteins.^15^ Network building and pathway enrichment using MetaCore and R created a set of network modules associated with input proteomic data or single-cell transcriptomic data. The algorithm was evaluated for creating modules that have higher than random saturation with the genes/proteins of interest. MetaCore calculated *P* values for the networks generated on the basis of hypergeometric distribution and evaluated its relevance to gene ontology biological processes. Benjamini-Hochberg correction was done in the network comparisons to avoid “the multicomparison problem” by adjusting individual *P* values from every pairwise comparison for significance (false discovery rate <0.001). Metabolomics data were tested (Metabolon) using standard statistical analyses (*t* test and ANOVA) in ArrayStudio on log-transformed data. Single-cell data were analyzed using principal component analysis using the genes accounting for the high variability across all the cells. The resulting top principal components accounting for the 95% variability between cells were used for tSNE dimensionality reduction (SeqGeq).

## Results

### Vein Graft Lesion Development in Fat-Fed *Ldlr*^−/−^ Mice

**Figure 1.**
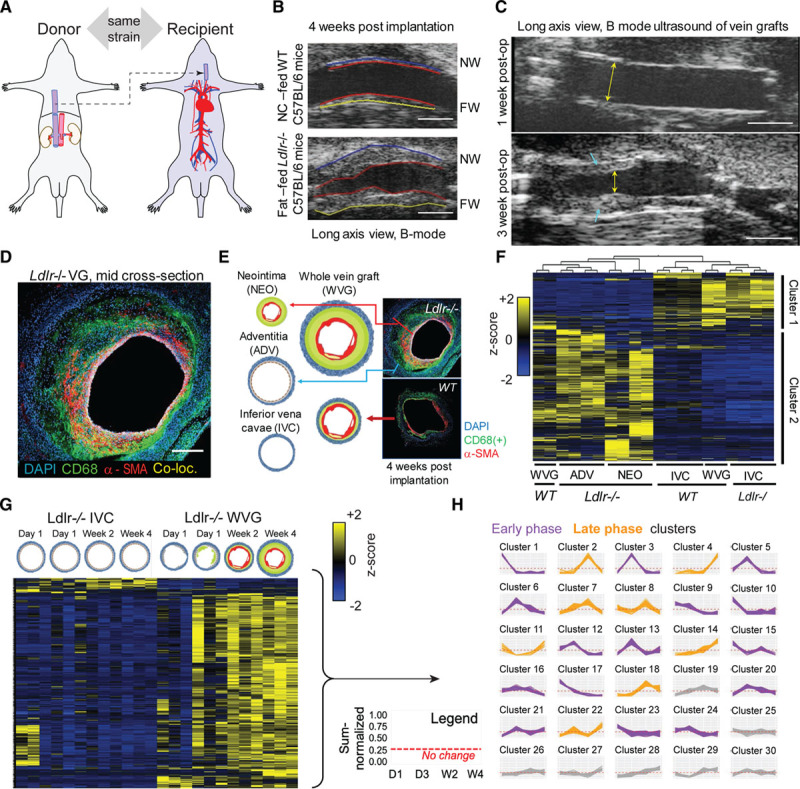
**Vein graft target discovery.** **A**, Mouse model: donor’s suprahepatic inferior vena cava (IVC) transplanted into the recipient’s left midcommon carotid artery. **B**, Normal chow (NC)–fed wild-type (WT) C57BL6 12-week-old male mice and fat-fed (2 weeks prefed) low-density lipoprotein receptor (*Ldlr*^−/−^) 12-week-old male mice (C57BL6 background) vein grafts in ultrasound imaging (long-axis view) at week 4 after operation. (n=6 versus n=6). Near wall (ventral) = NW, far wall (dorsal) = FW. Scale =1 mm. **C**, Long axis view (scale bar 1 mm) of a representative vein graft (VG) lesion showing an increase of lesion size (blue arrowheads) from 1 week to 3 weeks after operation. Luminal stenosis evident at 3 weeks (yellow arrows) **D**, Immunofluorescence of vein graft at 4 weeks after operation using AF488-anti-CD68 (macrophages, green color), Cy3-α-smooth muscle actin, SMA (vascular smooth muscle cells, VSMCs, red color), and DAPI (4′,6-diamidino-2-phenylindole, nucleus, blue color). Co-loc. indicates co-localization. Scale=100 µm. **E**, VG tissue layer dissection of neointimal (NEO) and adventitial (ADV) layers of *Ldlr*^−/−^ VG samples. WT VG samples were not microdissected. IVC was used as controls. Representative immunofluorescence staining of *Ldlr*^−/−^ versus WT VG tissue (n=2 biological replicates, n=2 technical replicates). **F**, Tissue layer proteomics (n=2 mice, 2 technical replicates per tissue) by multigroup comparison, false discovery rate ≤0.05. Proteins that are relatively increased in IVC samples and VG of 1 WT animal are also relatively decreased in *Ldlr*^−/−^ VG tissues in both NEO and ADV layers and in 1 VG of another WT animal, and vice versa. **G**, Time course proteomics of *Ldlr*^−/−^ VGs: 1 day (D1), 3 days (D3), 14 days (W2), and 28 days (W4) after VG surgery (n=12 VG donors, 3 biological replicates per time point). Vein grafts were processed as a whole (no layer dissection) and paired with matching IVC controls from the same animals. **H**, Multiplexed analysis of proteome across time points showing “coabundance” proteins in time (see Expanded Methods in the Data Supplement).

The inferior vena cava of syngeneic donor mice was anastomosed end-to-end to the carotid artery to create vein grafts (Figure 1A).^16,17^ The vein graft wall at 4 weeks after implantation was thicker in fat-fed *Ldlr*^−/−^ mice compared with normal chow–fed C57BL/6 wild-type mice (Figure 1B, Figure IA in the Data Supplement). Fat-fed *Ldlr*^−/−^ mice showed accelerated lesion development (Figure 1C, Figure IB in the Data Supplement) with the midvein cross-sectional histology (Figure IC and ID in the Data Supplement) correlating with 3-dimensional ultrasound volumes (Figure IE–IH in the Data Supplement). Vein graft lesions harbored macrophages and smooth muscle cells (Figure 1D). There were more circulating CD45+ cells in fat-fed *Ldlr*^−/−^ mice than in normal chow–fed wild-type mice (Figure I-I and IJ in the Data Supplement), with Ly6C^++^ monocytes proportionally higher, whereas CD19^+^ B cells and CD3^+^ T cells fractions remain even (Figure IK in the Data Supplement).

### Label-Free Proteomic Profiling of Vein Graft Tissues

Global proteomics for target discovery used neointimal (NEO) and adventitial (ADV) layers dissected from 4-week vein grafts from 2 *Ldlr*^−/−^ mice along with their equal-length nonarterialized endogenous vein controls, the inferior vena cava (IVC) of each mouse (Figure 1E). These were paired with 2 age-matched wild-type mouse vein grafts and IVC controls whose relatively thinner layers prevented accurate dissection. A total of 1357 proteins were quantified (Expanded Methods in the Data Supplement), then filtered using a multigroup comparison, resulting in 729 proteins (Figure 1F).

Cluster 1 (Figure 1F, upper) included proteins that were either predominantly expressed in the IVC samples and in 1 wild-type vein graft with minimal to nonexistent plaque formation (Figure IG in the Data Supplement) or diminished in the NEO and ADV samples. Conversely, Cluster 2 (Figure 1F, lower) includes proteins that are predominant in the NEO and ADV of *Ldlr*^−/−^ vein grafts (thick plaque, Figure IH in the Data Supplement) or diminished in the IVC and “nonplaque” wild-type vein graft sample. Although NEO and ADV layers are distinct and expected to have different proteome profiles, multigroup comparison analysis revealed them as part of 1 major cluster on the basis of principal component analysis (Figure IIA in the Data Supplement), and first-level hierarchical clustering because of their proteome similarity when compared against IVC samples (along with 1 lesion-free wild-type vein graft specimen). Even when considering only *Ldlr*^−/−^ samples, NEO and ADV proteomes are still clustered together, apart from the IVC (Figure IIB and IIC in the Data Supplement). However, only by omitting the IVC can group comparison show a statistical difference between NEO and ADV as depicted in principal component analysis and hierarchical clustering, with only 33 proteins accounting for variability between the 2 sample types (Figure IID and IIE in the Data Supplement). Hence, we considered NEO and ADV to be 1 group compared with the IVC counterparts.

Because the vein graft lesion increases gradually across 4 weeks (Figure IIIA in the Data Supplement), the kinetics of these molecular signatures was monitored. We performed a time course proteome profile of developing vein grafts at day 1, day 3, week 2, and week 4 after implantation in *Ldlr*^−/−^ animals. IVCs were time point–matched (Figure 1G; Figure IIIA in the Data Supplement). A heat map and principal component analysis of statistically filtered protein abundances for each sample further underscore the contrast between the changing vein graft proteome and the relatively unchanged IVC proteome in the time course proteomics (Figure 1G; Figure IIIB and IIIC in the Data Supplement).

To parse proteins on the basis of kinetic profiles, we performed cluster analysis XINA established by us^15^ that permits combining protein kinetic profiles from both *Ldlr*^−/−^ vein grafts and IVC tissues for a single clustering step and output (Figure 1H). Within each cluster trend line containing both vein graft and IVC proteins, only the proteins uniquely present in vein grafts, but not on IVCs, maybe the relevant molecular signatures for the diseased vein (vein grafts). Day 1 and day 3 time points may include immediate changes and adaptations of vein grafts and exposure to arterial flow. Early thrombosis and inflammatory damage may come into play during these early time points. Clusters 1, 3, 5, 6, 9, 10, 12, 13, 15, 16, 17, 20, 21, 23, and 24 represent proteins that are abundant during these early time points in both the vein graft and the IVC samples and are depicted in purple line graphs (Figure 1H). Later time points may characterize factors responsible for accelerated lesion development, thickening, or adverse remodeling of the plaque that is evident during imaging (Figure IIIA in the Data Supplement). Clusters 2, 4, 7, 8, 11, 14, 18, and 22 represent proteins abundant during the late phase: week 2 and week 4 after vein graft surgery in both the vein graft and the IVC samples are depicted in orange line graphs. The minimally changing trend which were clusters 19, 25, 26, 27, 28, 29, and 30 were omitted to focus on proteins with more significant temporal fluctuation (Figure 1H). Proteins derived from IVC samples in early- or late-phase clusters were removed from further analysis.

A comparison of the static and kinetic proteomic experiments demonstrates some concordance between the 2 studies. The IVC layer (static) shares more of the predominant proteins with the IVC time course than the vein graft time course (Figure IIID and IIIF in the Data Supplement). The NEO+ADV layer (diseased vein graft) shares more predominant proteins with the vein graft time course than with the IVC time course (Figure IIIE and IIIG in the Data Supplement).

### Network Analysis, Pathways Enrichment, and Target Prioritization Reveal PPARα as a Target Candidate

**Figure 2.**
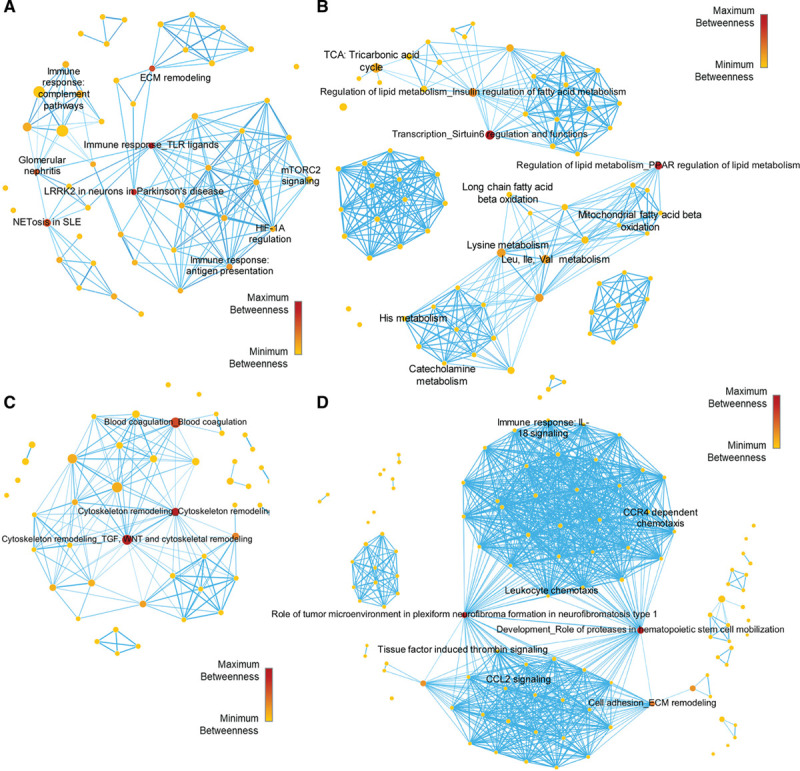
**Pathways network of static proteomics.** **A**, Low-density lipoprotein receptor (*Ldlr*^−/−^) vein graft (VG) neointimal (NEO)/adventitial (ADV) predominant proteins pathways enrichment network. **B**, Inferior vena cava (IVC) and wild-type (WT) VG predominant proteins pathways enrichment network. **C**, *Ldlr*^−/−^ VG early phase predominant proteins pathways enrichment network. **D**, *Ldlr*^−/−^ VG late phase predominant proteins pathways enrichment network. Node sizes are proportional to the number of dataset proteins present in that pathway node. Node color and scale bar depict the level of betweenness centrality for that pathway node.

We constructed our pathways networks (Figure 2) from proteins filtered from the week 4 end point vein graft tissue layer–static proteome (Figure 1F) and from the kinetic proteome profile of the whole vein graft lesion development (Figure 1G). For the first tissue layer network (Figure 2A, Table I in the Data Supplement), we input proteins elevated in lesion-positive *Ldlr*^−/−^ NEO and ADV layers or wild-type whole vein grafts (cluster 2 in Figure 1F). The lesion-positive static proteome which defined, for instance, the *Ldlr*^−/−^ NEO and ADV layers, showed enriched biological processes associated with inflammation and extracellular matrix remodeling (Figure 2A, Figure IVA in the Data Supplement, in a higher resolution).

The second tissue network (Figure 2B, Table II in the Data Supplement) was generated from proteins higher in the IVC controls of both *Ldlr*^−/−^ and the wild-type mouse with minimal lesion development (cluster 1, Figure 1F). This IVC proteome enriched mainly mitochondrial metabolic processes such as nonglycolytic bioenergetics (ie, PPARα regulated lipid metabolism, tricarboxylic acid (TCA) cycle, and amino acid metabolism) (Figure 2B, Figure IVB in the Data Supplement). Similarly, we constructed pathway network modules for the early-phase and late-phase filtered proteins (Figure 2C and 2D, Figures V and VI in the Data Supplement, Tables III and IV in the Data Supplement). The early-phase kinetic clusters protein-enriched pathways associated with blood coagulation, cytoskeleton remodeling, and inflammasomes may relate to the initial tissue damage response to vein implantation into an arterial environment (Figure 2C, Figure V in the Data Supplement). As the vein graft matures and the lesion further develops, the late-phase kinetic clusters (Figure 2D, Figure VI in the Data Supplement) enriched biological processes related to extracellular matrix remodeling, further inflammation, thrombin signaling, and leukocyte chemotaxis.

Enriched pathways (nodes) in each group were connected through shared proteins to form a network. Intermediary pathways in central-most network positions act as a primary conduit for “passing” information between the “nonshared proteins” pathways. By this “traffic” conduit logic,^18^ the 3 top-ranked central pathways/nodes for each sample-condition network may contain the most desirable target(s). Pathways with high centrality may serve as key pathobiological roles in disease processes, as we demonstrated.^19^ The top 3 biological processes for each dataset and the most central protein within each top-ranked pathway were identified (Table V in the Data Supplement). By plotting each protein’s betweenness centrality versus closeness centrality (Figure VIIA in the Data Supplement), betweenness centrality versus degree centrality (Figure VIIB in the Data Supplement), and closeness centrality versus degree centrality (Figure VIIC in the Data Supplement), a consensus was attained for the most central proteins for each pathway: SIRT6, PPARα, SREBP1, thrombin, Rictor, PAK, LRRK2, TLR2, SDF-1, MMP-13 (matrix metalloproteinase 13), and c-kit (Table V in the Data Supplement). Among them, we selected PPARα (Figure VIID in the Data Supplement) as an example to verify that our target discovery platform can identify pathways/molecules that indeed play causal roles in the pathogenesis of vein graft lesion development.

### Macrophage PPARα siRNA Silencing Accelerates Vein Graft Lesion Development

**Figure 3.**
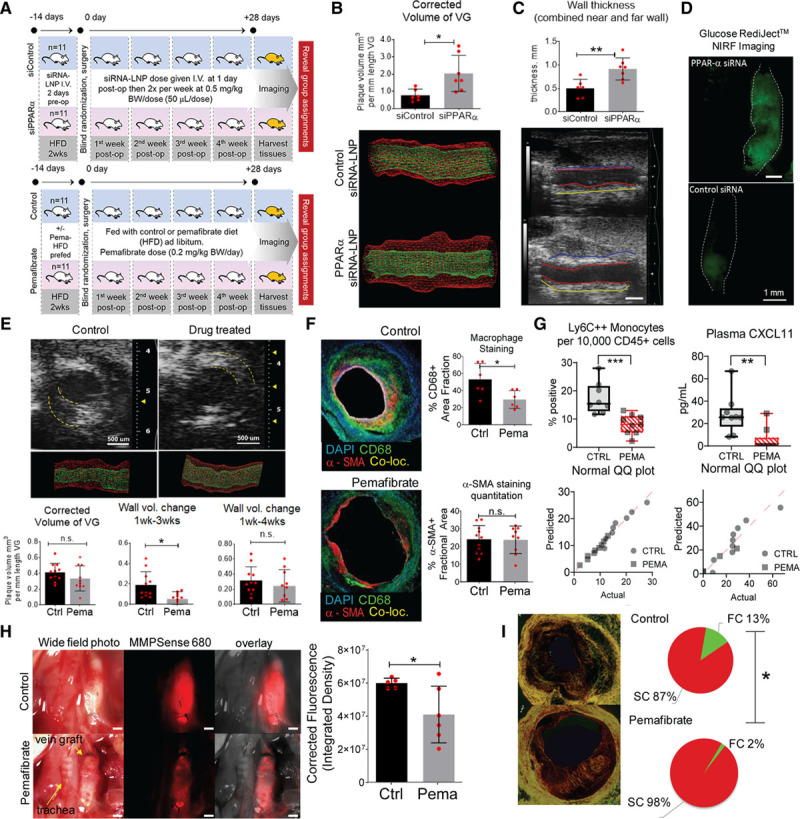
**In vivo PPARα loss-of-function and gain-of-function studies.** **A**, Study design. **Upper**, Loss-of-function. Twenty-two low-density lipoprotein receptor (*Ldlr*^−/−^) male mice used, aged 12 weeks, prefed with high-fat diet 2 weeks before operation. Randomization in 2 treatment groups: (1) set 1: siRNA control conjugated to C12-200 lipid nanoparticles (LNP); (2) set 2: siRNA PPARα (small interfering RNA or siRNA of PPAR alpha, 1:1 mixture of oligonucleotide 1 and 2 conjugated to C12-200 LNP. A 0.5 mg siRNA-LNP/kg body weight (BW)/dose given intravenously 11 times throughout the study. Dosage schedule: 2 days before operation, right after surgery, then 2 times per week (every 3 days) after surgery for 4 weeks with 1 extra dose 2 to 3 days after day 28. **Lower**, Pemafibrate (PPARα gain-of-function) study (n=11 versus n=11). **B**, Three-dimensional ultrasound rendered wall volume comparison of siControl (siControl= non-specific small interfering RNA, siRNA), and siRNA PPARα silenced group at 4 weeks after surgery. **C**, Vessel wall thickness, long-axis view. siRNA PPARα group had thicker vessel walls than siRNA control group (n=11 versus n=11). Scale=1 mm. **D**, Glucose uptake by RediJect assay (Perkin Elmer) and intravital fluorescence in the VG. Representative image. **E**, Gain-of-function study using the highly selective PPARα activator pemafibrate (drug) (n=11 versus n=11, control versus drug-treated) resulted in lesser neointimal plaque up to 3 weeks after surgery. **F**, Immunofluorescence histology of the midgraft transverse sections using CD68 (green) and α-SMA (red) antibodies (blue=DAPI [4′,6-diamidino-2-phenylindole], nuclear stain). Pemafibrate-treated group had less macrophage accumulation at the lesion than control group. There was no difference in vascular smooth muscle cell content. **G**, Fluorescence-activated cell sorting analysis showing pemafibrate decreased circulating Ly6C^++^ monocytes. ELISA of blood plasma shows pemafibrate decreases plasma recruitment chemokine CXCL11 levels. Corresponding normality tests. **H**, In situ transmitted light and intravital near-infrared fluorescence (NIRF) imaging of vein grafts 12 hours after MMPSense 680 intravenous injection. Red fluorescence indicates intensity of proteases MMP-2, 3, 9, and 13 (activity and relative abundance). **I**, Picrosirius red staining (PSR) of 4-week vein grafts midcross-section with quantification of red and green birefringence of collagen fibrils. *P<0.05; **P<0.01; ***P<0.001; n.s., *P*≥0.05. Co-loc indicates colocalization; Ctrl, control; FC, fragmented collagen; HFD, high-fat diet; LNP, lipid nanoparticle; Pema, pemafibrate; post-op, after operation; PPARα, peroxisome proliferator-activated receptor α; QQ or Q-Q, quantile-quantile; SC, stable collagen; siRNA, small interfering RNA; SMA, smooth muscle actin; and VG, vein graft.

Testing PPARα loss-of-function in macrophages on vein graft lesions used in vivo delivery of PPARα siRNA or control siRNA encapsulated in macrophage-targeted lipid nanoparticles C12–200.^17,20–22^ We confirmed PPARα silencing in peritoneal macrophages and splenic mononuclear cells from *Ldlr*^−/−^ mice (Figure VIIIA and VIIIB in the Data Supplement). The entire study was conducted blindly until all analyses were finalized (Figure 3A, upper). The ultrasound 3-dimensional measurements showed that vein grafts of siPPARα (small interfering RNA of PPARa)-treated mice had higher wall volume and thickness than controls (Figure 3B and 3C). Enhanced glucose uptake, a feature of inflamed tissue, appeared increased in the PPARα siRNA versus control as well (Figure 3D).

### PPARα Activation Reduces Vein Graft Lesion Burden and Inflammation

To clarify in vivo evidence for the suppressive role of PPARα in vein graft lesion development, the gain-of-function study (Figure 3A, lower) used the first-in-class highly selective PPARα modulator pemafibrate in a blind fashion.^23,24^ Pemafibrate (0.2 mg/kg body weight per day) admixed with the diet lessened the volume of the developing lesion up to the third week (3-dimensional ultrasonography, Figure 3E). At the 4-week time point, although the control group had a higher mean wall volume increase compared with the drug-treated group, this difference was statistically insignificant. The control group showed increased weight gain after the second week postoperatively (Figure VIIIC in the Data Supplement) despite similar food consumption (Figure VIIID in the Data Supplement).

The neointima of the pemafibrate-treated grafts at the 4-week time point contained fewer macrophages than those of control grafts, whereas smooth muscle cell content did not differ (Figure 3F). MMP-9 and MMP-13 staining was less in pemafibrate-treated mice (Figure IXA in the Data Supplement). We chose the dose of pemafibrate that would not affect plasma triglyceride, cholesterol, and glucose levels to examine whether the effects of PPARα activation are independent of changes in the blood lipid profile. There were no differences between nonfasted control and drug treatment groups at 4 weeks after surgery (Figure IXB in the Data Supplement). However, in a separate no-surgery experiment, in high fat–fed *Ldlr*^−/−^ mice, fasting plasma triglyceride levels decreased in the pemafibrate-treated group but not the fasting plasma cholesterol or glucose levels (Figure IXC in the Data Supplement). Circulating Ly6C^++^ monocytes decreased in the pemafibrate-treated group but not the B cells or T cells (whole blood flow cytometry, Figure 3G, Figure IXD in the Data Supplement). Plasma levels of CXCL11, a proinflammatory monocyte-recruiting chemokine, also decreased in the pemafibrate group (Figure IXD in the Data Supplement). Flow cytometry counts per group were identical (Figure IXE in the Data Supplement). A Spearman correlation plot between fasting triglyceride versus circulating Ly6C^++^ monocytes did not indicate that these parameters correlate with each other (Figure IXF in the Data Supplement), suggesting that lowering triglyceride levels may not have caused a reduction in circulating proinflammatory monocytes. In the PPARα loss-of-function study, fasting lipid levels did not change (Figure IXG in the Data Supplement).

Plaque rupture may occur in inflamed human vein grafts^11,12^ where macrophages may secrete MMPs that degrade fibrillar collagen, a determinant of plaque stability. MMP-9 and MMP-13 staining showed lower expression in vein grafts of pemafibrate-treated mice (Figure IXA in the Data Supplement). Intravital molecular imaging using the MMP-activatable (MMP-9 and MMP-13) near-infrared fluorescence substrate tracer MMPSense 680 (PerkinElmer, MA) showed lower activity in the pemafibrate group (Figure 3H), supporting immunostaining data (Figure IXA in the Data Supplement). Collagen hue analysis of picrosirius red staining viewed under polarized light demonstrated a decrease in green birefringence of fragmented collagen fibers in pemafibrate-treated vein graft lesions compared with control lesions (Figure 3I).

### Disease Network Proximity Analysis Links the Vein Graft Proteomics Network With Human AVF Disease Network

**Figure 4.**
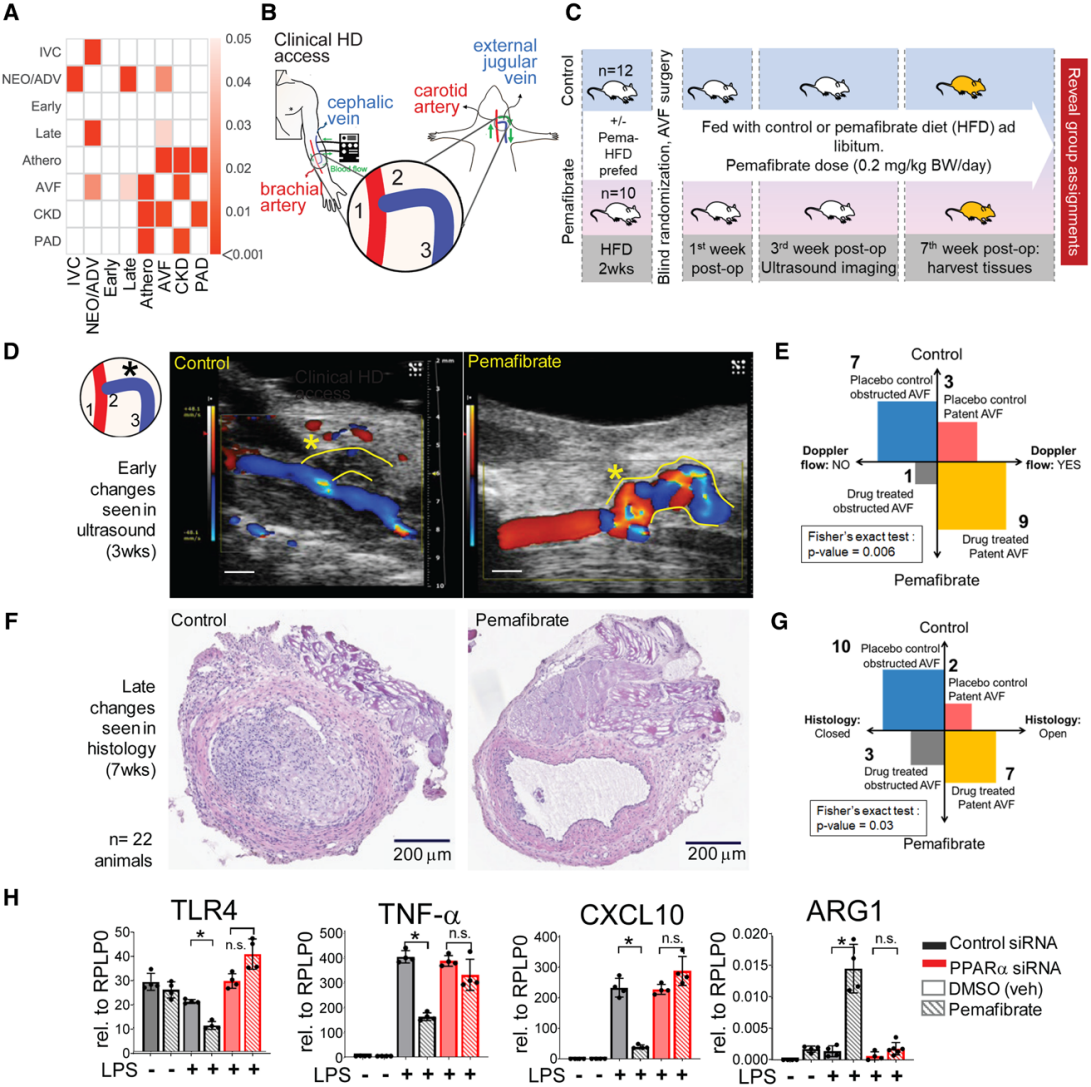
**Experimental AVF failure (disease) and PPARα gain-of-function study.** **A**, Heatmap of associations between modules of proteomics and diseases of interest, measured in terms of network closeness. Empirical *P* values are calculated by 1000 randomizations and corrected for multiple testing by the Benjamini-Hochberg procedure. Darker shades indicate a higher significance, whereas insignificant associations (empirical *P* value >0.05) are indicated as blank cells. **B**, Human AVF access model using low-density lipoprotein receptor (*Ldlr*^−/−^) mice with side-to-end anastomosis of the left external jugular vein (EJV, blue, 2, 3) to the midportion of the left common carotid artery (CCA, red, 1). **C**, In vivo study design with randomization in 2 treatment groups: (1) animals are prefed with HFD only or (2) HFD + pemafibrate ≈ dose of 0.2 mg/kg BW/d, at 2 weeks before surgery and continued to 7 weeks after surgery. Color Doppler ultrasound (Vevo 2100) of AVF at 3 weeks after surgery. At 7 weeks after surgery, AVFs were harvested for histology. **D** and **E**, Color Doppler shows better patency (*) and blood flow through AVFs of the pemafibrate-treated group. If there was color Doppler signal in the a-EJV (anastomosis-connected external jugular vein), we called it as “open” or “patent” because of high-velocity turbulent flow of blood as detected by the color Doppler. If there was no color Doppler signal, we called this as “nonpatent” or “closed.” VevoLab software version 1.6.0 build 6078 (Fujifilm) was used in the assessment. **F** and **G**, Late change in histology (hematoxylin and eosin [H&E] staining) shows the pemafibrate-treated group had less neointima as measured in the mid CSA portion of the proximal (to the anastomosis) third of the venous limb of the AVF. **H**, Bulk quantitative polymerase chain reaction of bone marrow–derived mouse macrophages for inflammatory markers with either gain-of-function or loss-of-function of PPARα. *P<0.05; n.s., *P*≥0.05. Athero indicates atherosclerosis; AVF, arteriovenous fistula; BW, body weight; CKD, chronic kidney disease; Cont., control; CSA, cross-sectional area; DMSO, dimethyl sulfoxide; FDR, false discovery rate; HD, hemodialysis; HFD, high-fat diet; IVC, inferior vena cava predominant proteins; LPS, lipopolysaccharide; NEO/ADV, combined neointimal and adventitial layer predominant proteins; PAD, peripheral artery disease; Pema., pemafibrate; post-op, after operation; PPARα, peroxisome proliferator-activated receptor α; siRNA, small interfering RNA; and veh, vehicle.

To highlight clinical translatability of this target discovery platform, we evaluated the network proximity of the vein graft disease module to the gene modules for 4 linked or allied human vascular diseases^25^: atherosclerosis/coronary artery disease, AVF failure, chronic kidney disease with diabetes, and PAD. Because of scant information about human gene–associated vein graft disease or failure, the human vein graft disease–gene module remained unbuilt. An N × N plot (Figure 4A) showed false discovery rate values <0.05 (Benjamini-Hochberg correction) in dataset modules that have close associations by “first neighbor” proteins or shared proteins. The NEO/ADV predominant proteins network module has close associations with the early- and late-phase proteins but not the IVC module from vein graft tissue. The NEO/ADV and late time point vein graft modules also have close associations with the human AVF failure. It indicates that our experimental vein grafts share some similar pathological processes with AVF failure, another arterialized vein disease.

### PPARα Activation Reduces Lesion Size and Increases Patency in Experimental AVF in Mice

Experimental AVF construction in fat-fed *Ldlr*^−/−^ mice used a previously reported technique.^26^ An end (of vein)-to-side (of artery) anastomosis of the left external jugular vein to the midportion of the left carotid artery was performed in the same mouse (Figure 4B). The in vivo study, carried out blindly, tested whether PPARα activation by pemafibrate would improve AVF patency and retard lesion progression (Figure 4C). Three weeks after surgery, pemafibrate-treated mice had more patent AVF and better blood flow by color Doppler (Figure 4D and 4E). At 7 weeks after surgery, pemafibrate-treated animals had more patent arterio-venous fistulas as determined histologically (Figure 4F and 4G).

### PPARα Activation Mitigates Proinflammatory Activation of Mouse Macrophages In Vitro

In vitro gain-of-function and loss-of-function assays on mouse bone marrow–derived macrophages demonstrated that pemafibrate-induced PPARα activation reduced expression of proinflammatory factors *Tlr4*, *Tlr2, Tnf*α, *Il6, Il1β*, *Cxcl9, Cxcl10*, and *Cxcl11* in lipopolysaccharide-elicited macrophages (Figure 4H, Figure XA–XC in the Data Supplement). Patterns of differential gene expression after PPARα silencing mimicked the lipopolysaccharide (alone) condition, despite pemafibrate treatment. Arginase 1 and chitinase like-3 (Ym1), molecules associated with reparative macrophage polarization,^27,28^ increased after PPARα activation and fell after PPARα silencing (Figure 4H, Figure XD in the Data Supplement). Ym1, detected in the vein graft time-course proteomics, decreased from day 1 to week 4 (Figure XE in the Data Supplement), coincident with plaque increase and growth. Yet IVC Ym1 levels were unchanged (Figure XE in the Data Supplement). To further evaluate PPARα’s role, we proceeded with single-cell RNA sequencing (Figure XF in the Data Supplement) and single-cell quantitative polymerase chain reaction (Figure XG in the Data Supplement) on primary human macrophages.

### Single-Cell Transcriptomics of Proinflammatory Macrophages Identifies a Distinct Inflammatory Cluster of Cells Among Lipopolysaccharide-Stimulated Macrophages M(LPS)

**Figure 5.**
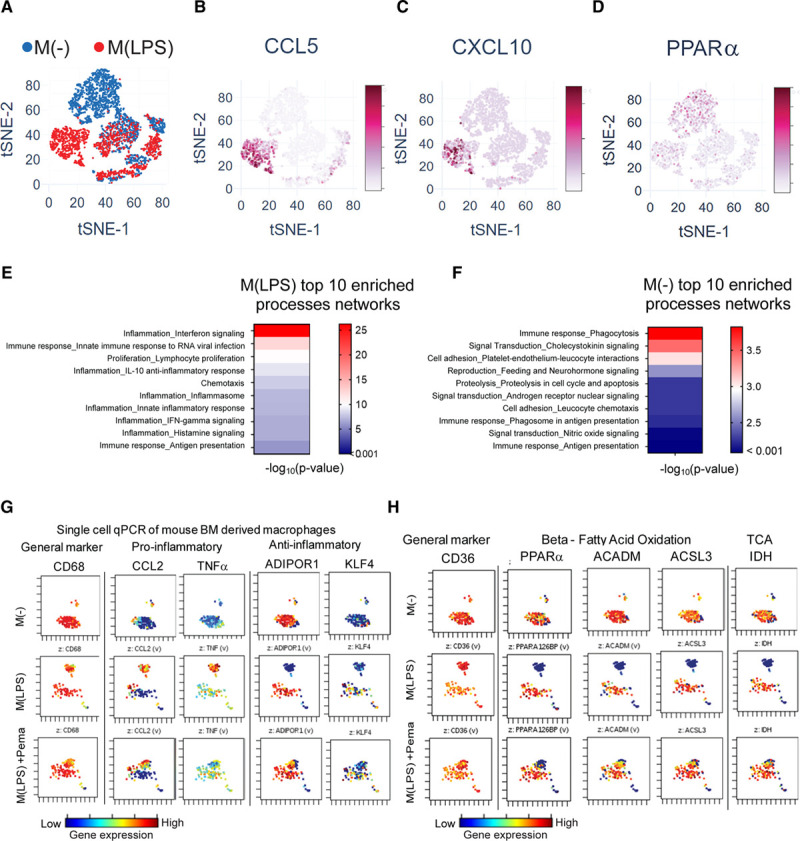
**In vitro validation: single-cell mRNA expression in primary macrophages.** **A**, tSNE of single-cell RNA sequencing data of M(–) and M(LPS) primary human macrophages (2557 cells M(–), 1680 cells M(LPS), single donor). Gene expression projected onto tSNE plot of CCL5 (**B**), CXCL10 (**C**), and PPARα (**D**). **E**, Top 10 enriched process networks for M(LPS) cluster 1 differentially expressed genes relative to M(–) cluster 1 genes. **F**, Top 10 enriched process networks for M(–) cluster 1 differentially expressed genes relative to M(LPS) cluster 1 genes. **G** and **H**, Single-cell quantitative polymerase chain reaction (qPCR) of human PBMC-derived primary macrophages (from 3 donors) in 3 conditions: (1) M(–): LPS(–), DMSO; (2) M(LPS): LPS(+), DMSO; (3) Pema + M(LPS): LPS(+), pemafibrate. ACADM indicates acyl-CoA dehydrogenase, medium-chain; ACSL3, acyl-CoA synthetase long-chain family member 3; ADIPOR1, adiponectin receptor 1; BM, bone marrow; CCL2, C-C motif chemokine ligand 2; DMSO, dimethyl sulfoxide; IDH, isocitrate dehydrogenase; IFN, interferon; KLF4, Krüppel-like factor 4; LPS, lipopolysaccharide; M(–), unstimulated or baseline macrophage; M(LPS), LPS-stimulated macrophage; PBMC, peripheral blood mononuclear cell; PBS, phosphate-buffered solution; PPARα, peroxisome proliferator-activated receptor α; siRNA, small interfering RNA; TCA, tricarbonic acid cycle; and TNF, tumor necrosis factor.

Single-cell RNA sequencing (Expanded Methods in the Data Supplement, Figure XF in the Data Supplement) on the baseline or unstimulated (M(–)) and M(LPS) primary human macrophages revealed a heterogeneous population (Figure 5A). The M(LPS) macrophage population exhibits a distinct cluster, M(LPS) cluster 1, that does not contain M(–) cells within, on the basis of tSNE plot coordinates (Figure XIA in the Data Supplement). This observation that activated macrophages remain heterogeneous rather than simply polarized was consistent with our previous single-cell analysis data in primary human macrophages elicited with interferon-γ or indoxyl sulfate.^20,29^ This cluster harbor cells with the most expression of proinflammatory genes (Figure 5B and 5C, Figure XIB and XIC in the Data Supplement). The M(–) population also exhibits a distinct cluster, M(–) cluster 1, that does not appear to contain any M(LPS) cells or have a high expression of proinflammatory genes, but has a high expression of PPARα (Figure 5D) and genes associated with the TCA cycle (Figure XID in the Data Supplement). On the basis of the differential gene expression between these 2 clusters, we identified which genes are at least 1.5-fold increased in 1 cluster versus the other (Figure XIB in the Data Supplement). MetaCore analysis on each set of differentially increased genes reveals each cluster’s enriched biological processes. The top-ranked processes in the M(LPS) cluster 1, relative to the M(–) cluster 1, are associated mainly with proinflammatory signaling (Figure 5E), whereas M(–) cluster 1, relative to M(LPS) cluster 1, was related with phagocytosis and chemotaxis (Figure 5F).

Single-cell quantitative polymerase chain reaction of primary human macrophages measured the expression of 32 genes corroborating cell heterogeneity reported above. We also confirmed the presence of the inflammatory cluster of M(LPS) cells and PPARα activation’s effect on this cluster. In total, 250 cells per condition were used from 3 pooled donors: M(–), M(LPS), and pemafibrate-treated M(LPS). M(–) had less variability among cells (Figure 5G). On LPS stimulation, however, a small cluster separated from the larger cluster. With PPARα activation by pemafibrate in M(LPS) cells, the separation between these clusters appeared less distinct. Reference mRNAs changed minimally (Figure XIIA in the Data Supplement). Proinflammatory mediators tumor necrosis factor-α, interleukin-6, CCL2, and interleukin-1β expression increased in the small cluster of M(LPS) cells but decreased in a similar cluster on PPARα activation (pemafibrate-treated M(LPS)) (Figure 5G, Figure XIIB in the Data Supplement). In contrast, PPARα and genes related to fatty acid oxidation and other oxidative pathways (Figure 5H, Figure XIIC in the Data Supplement) had lower expression in the small cluster at the LPS condition but increased on PPARα activation.

### Metabolomic Profiling Reveals Metabolic Reprogramming of Activated Human Primary Macrophages Via PPARα Activation

**Figure 6.**
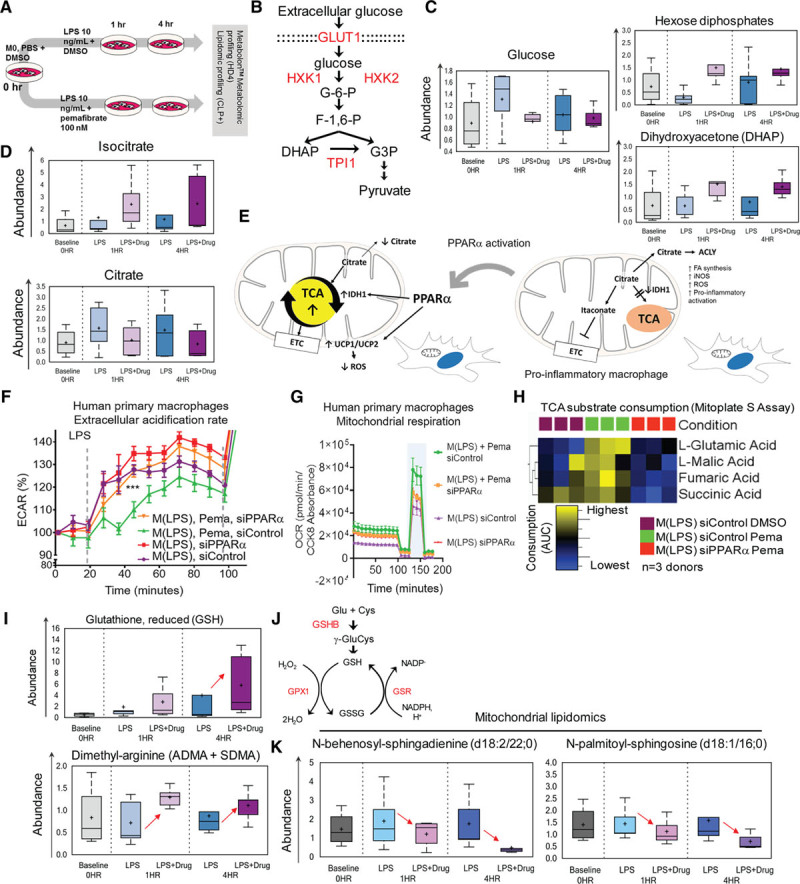
**Whole-cell metabolomics and metabolic function of PBMC-derived macrophages.** **A**, Human PBMC-derived macrophages—metabolomic profiling study (Metabolon, see Expanded Methods in the Data Supplement). n=5 donors per time point; samples were processed for (1) whole cell lysate and (2) isolated mitochondria lysate metabolomic and lipidomic screening. **B**, Glycolysis pathway. PPARα transcription-regulated enzymes (red). **C**, Glucose, hexose diphosphates, and DHAP metabolite levels. **D**, Isocitrate and citrate metabolite levels **E**, PPARα activation may release the TCA block in M(LPS) cells. **F**, Silencing PPARα of M(LPS) + Pema raises ECAR to M(LPS) levels. siControl (M(LPS) + Pema) have lower ECAR and **G**, higher OXPHOS rate (increase in OCR) and maximal respiratory reserve (blue band). **H**, TCA substrate consumption as measured by Mitoplate S assay (Biolog). **I**, Reduced form of glutathione is relatively increased in M(LPS +Pema) at the 4-hour time point. Glutathione pathway/cycle. PPARα transcription regulated enzymes (red). **J**, Asymmetrical and symmetrical dimethylarginine metabolites (ADMA, SDMA, whole-cell lysate) levels. **K**, Ceramides: N-behenoyl-sphingadenine (d18:2/22:0) and N-palmitoyl-sphingosine (d18:1/16:0) mitochondria levels. ACLY indicates Adenosine triphosphate-Citrate Synthase; ADMA, asymmetrical dimethylarginine; AUC, area under the curge; DHAP, dihydroxyacetone phosphate; DMSO, dimethyl sulfoxide; ECAR, extracellular acidification rate; ETC, electron transport chain; FA, fatty acid; GSHB, glutathione synthetase; GSR, glutathione reductase; GSH, glutathione reduced form; GSSG, glutathione disulfide oxidized form; iNOS, inducible nitric oxide synthase; LPS, lipopolysaccharide; M0, unstimulated or baseline macrophage state or M(-); M(LPS), LPS-stimulated macrophage; NADP–, nicotinamide adenine dinucleotide phosphate; NADPH, nicotinamide adenine dinucleotide phosphate, reduced form; OCR, oxidative consumption rate; OXPHOS, oxidative phosphorylation; PBMC, peripheral blood mononuclear cell; Pema, pemafibrate 100 nmol/L; ROS, reactive oxygen species; SDMA, symmetrical dimethylarginine; siControl, control non-specific small interfering RNA; TCA, tricarboxylic acid; and UCP, uncoupling protein.

Because fatty acid oxidation is related to oxidative phosphorylation, PPARα activation may shift macrophage metabolic state by reprogramming the M(LPS) cells from a hyperglycolytic state to an oxidative phosphorylation–dependent state. We profiled metabolomic changes in peripheral blood mononuclear cell–derived human macrophages during proinflammatory activation and subsequent PPARα activation using the HD4 and CLP^+^ metabolomics platforms (Metabolon). Peak extracellular acidification rate (≈ glycolytic rate) of bone marrow–derived macrophages occurred between 60 and 65 minutes after lipopolysaccharide stimulation (Figure XIIIA in the Data Supplement). Given this information, we designed an in vitro timed assay for the changing metabolome (3 time points: 0 hour, 1 hour, and 4 hours, Figure 6A). We analyzed whole-cell metabolites, whole-cell lipids, and metabolites from isolated mitochondria.

Glucose intracellular uptake and glycolysis involve several proteins regulated by PPARα: GLUT1, HXK, and TPI1 (Figure 6B). Whole-cell metabolomics showed elevated intracellular glucose both 1 and 4 hours after LPS stimulation of human primary macrophages but decreased on PPARα activation, whereas hexose diphosphates and dihydroxyacetone phosphate (DHAP) increased (Figure 6C). Citrate was elevated in M(LPS), similar to reports of LPS-primed mouse macrophages,^30,31^ and decreased on PPARα activation. An increase of isocitrate, a TCA cycle intermediate, accompanied this shift (Figure 6D), suggesting a preferred feed-forward mechanism from glycolysis to TCA rather than the expected cytosolic escape of excess mitochondrial citrate during LPS activation (Figure 6E).^31^ In both primary human and mouse macrophages, on PPARα activation, the extracellular acidification rate remained low after LPS injection (arrow in Figure 6F, Figure XIIIB in the Data Supplement). In a glycolytic stress test, we examined glycolytic reserve, glycolytic capacity, and nonglycolytic acidification rate (Figure XIIIC–XIIIF in the Data Supplement). PPARα silencing in human M(LPS) increased glycolytic capacity. However, the addition of pemafibrate, given nonsilenced PPARα expression, increased glycolytic reserve (Figure XIIID and XIIIE in the Data Supplement).

Oxidative respiration related oxygen consumption rate and maximal respiratory reserve increased on additional PPARα activation (pemafibrate-treated M(LPS), light blue band in Figure 6G). PPARα silencing negated metabolic effects of pemafibrate on M(LPS) (Figure 6F and 6G). Thus, PPARα shifts bioenergetic preferences of M(LPS) from a highly glycolytic state to a lesser one accompanied by increased oxidative respiration (oxidative phosphorylation). A PPARα-mediated feed-forward mechanism to TCA among M(LPS) is demonstrated using the Mitoplate S assay (Biolog). TCA substrate utilization of fumarate, succinate, glutamate, malate, isocitrate, and α-ketobutyrate appear to show a trend of increasing rate on pemafibrate treatment and reversal on PPARα silencing (Figure 6H). However, only glutamate and succinate consumption showed statistically significant differences (Figure XIVA–XIVF in the Data Supplement). In comparing isolated functional mitochondria from M(LPS) + DMSO versus M(LPS) + pemafibrate–conditioned THP-1 macrophage-like cells, pemafibrate increased citrate, succinate, malate, and glutamate consumption (Figure XVA–XVD in the Data Supplement).

### PPARα Activation Regulates Macrophage Mitochondrial Fitness and Oxidative Damage

PPARα activation decreases nonmitochondrial oxygen consumption in LPS-treated mouse bone marrow–derived macrophages (Figure XIIIB in the Data Supplement), which may be a result of nicotinamide adenine dinucleotide phosphate, reduced form, oxidases and nitric oxide synthase (NOS) activity. PPARα activation increased asymmetric and symmetric dimethylarginine (ADMA and SDMA) levels (Figure XVIA and XVIB in the Data Supplement). ADMA can inhibit inducible NOS.^32^ ADMA and SDMA mediate how PPARα activation reduces NOS activity in a gain- and loss-of-function NOS activity assay in human primary macrophages (Figure XVIA–XVIC in the Data Supplement). NOS activity increases pro-oxidant stressors like reactive oxygen species that damages the mitochondrial membranes, which may be mitigated by PPARα activation through ADMA and SDMA, and by increasing the presence of antioxidants like reduced glutathione (Figure 6I and 6J)

**Figure 7.**
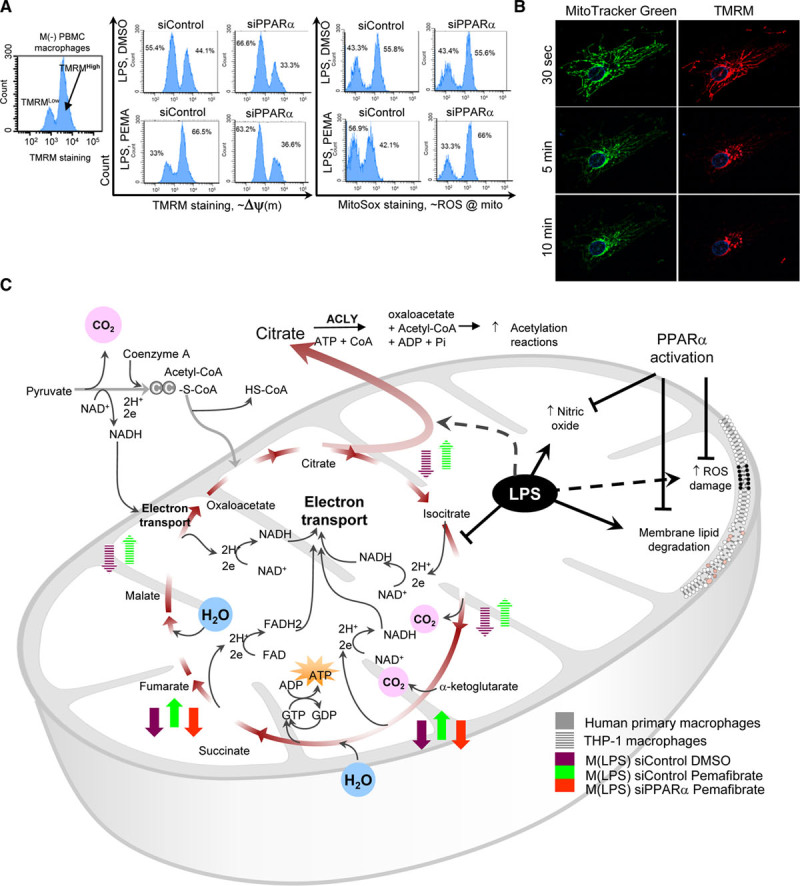
**Effects of peroxisome proliferator-activated receptor α (PPARα) on the mitochondria.** **A**, Baseline PBMC macrophages, M(–) with high TMRM staining indicates cells with high mitochondrial membrane potential. In M(LPS), low TMRM staining fraction increases, but pemafibrate promotes preservation of high TMRM fraction (high membrane gradient potential). Silencing PPARα abolishes this effect. Mitochondrial ROS damage ≈ high MitoSox staining (fluorescence-activated cell sorting). **B**, LPS-stimulated RAW264.7 cells show a decrease in mitochondrial TMRM staining, implying “leaky” membrane. Ten-minute time-lapse fluorescence **C**, Summary of TCA substrate consumption and mitochondrial membrane damage during LPS stimulation and how PPARα affects these changes. ACLY indicates adenosine triphosphate citrate lyase; FAD, flavin adenine dinucleotide oxidized; HS-CoA, free coenzyme A, hydrogen-sulfur; LPS, lipopolysaccharide; mito, mitochondria; M(–), unstimulated or baseline macrophage; M(LPS), LPS-stimulated macrophage; NAD, nicotinamide adenine dinucleotide oxidized form; NADH, nicotinamide adenine dinucleotide reduced form; PBMC, peripheral blood mononuclear cell; ROS, reactive oxygen species; siControl, control non-specific small interfering RNA; TCA, tricarboxylic acid; TMRM, tetramethylrhodamine methyl ester; and Δψ(m), mitochondrial membrane potential.

Mitochondria lipidomics showed that PPARα activation reduces sphingolipid degradation molecules such as ceramides in human M(LPS) (Figure 6K). It may indicate reduced degradation of sphingolipids from the mitochondrial membranes, preserving membrane integrity and fluidity. A resulting less “leaky” membrane maintains high mitochondrial membrane potential and “fitness.”^33,34^ M(–) macrophages contained more TMRM-high than TMRM-low cells (Figure 7A), similar to M(LPS) treated with pemafibrate, with no PPARα silencing (Figure 7A). In contrast, there is a lesser frequency of TMRM-high than TMRM-low cells in M(LPS) when PPARα is silenced or pemafibrate withheld (Figure 7A).

MitoSox staining, which indicates mitochondrial membrane oxidative stress damage, demonstrated that pemafibrate-treated M(LPS) without PPARα silencing (siControl) have minimal difference (count frequency) between high versus low MitoSox staining fractions compared with M(LPS) (Figure 7A). This difference in counts between high versus low MitoSox staining cells in pemafibrate-treated M(LPS) was abolished when PPARα was silenced (lower right quadrant of right panels in Figure 7A). High TMRM staining indicates high mitochondrial membrane gradient potential typically seen in healthy mitochondria with a large capacity for oxidative phosphorylation.^34^ Low TMRM staining is concordant with high MitoSox staining after lipopolysaccharide stimulation, as oxidative damage decreases the membrane gradient. TMRM staining decreased to minimal levels after 10 minutes in a RAW264.7 cell primed with lipopolysaccharide for an hour (Figure 7B).

### PPARα Increases Lipid Loading in M(LPS)

Lipidomic profiling revealed that treating proinflammatory M(LPS) macrophages with pemafibrate increased intracellular loading of cholesterol esters, mono-, di-, and triacylglycerols (Figure XVIIA–XVIID in the Data Supplement). However, functional analysis of fatty acid oxidation by kinetic substrate utilization assay (Biolog) of D,L-β-hydroxybutyric acid, and acetyl-L-carnitine with malic acid, on isolated mitochondria of M(LPS)-treated THP-1 cells shows increased rate utilization on pemafibrate treatment (Figure XVIIIE and XVIIIF in the Data Supplement). PPARα activation may increase the capacity of M(LPS) to utilize free fatty acids while increasing the capacity to load lipids.

### Metabolomic Data and the PPARα Regulatory Network Correlate With Macrophage Gene Expression and Vein Graft Proteomics

Vein graft proteomic profiles included differentially metabolic enzymes under the transcriptional control of PPARα (Figures XVIII and XIX in the Data Supplement). We therefore constructed a small “directed” gene regulatory network (Figure XX in the Data Supplement) demonstrating how PPARα may increase expression of the following enzymes: ACON (aconitase/aconitate hydratase), IDH (isocitrate dehydrogenase), PMRT1 (protein arginine-N-methyl transferase), GPX (glutathione peroxidase), GSR (glutathione reductase), GSHB (glutathione synthetase), and HXK1 and HXK2 (hexokinases). The network also shows how PPARα may decrease expression of the proteins GLUT1 (glucose transporter 1), TPI1 (triosephosphate isomerase), and ASM (acid sphingomyelinase), explaining the changing metabolites seen in M(LPS) treated with pemafibrate. In the mitochondrial lipidomic survey, sphingomyelin degradation (via ASM) by-products N-behenoyl-sphingadienine (d18:2/22:0) and N-palmitoyl-sphingosine decreased with PPARα activation in M(LPS) (Figure 6K). Intracellular glucose availability for glycolysis decreased in M(LPS) treated with pemafibrate. Glucose uptake in mouse vein grafts that received PPARα siRNA increased (Figure 3D). Increased hexose diphosphates and DHAP accumulation in the PPARα-activated M(LPS) over vehicle-treated M(LPS) (Figure 6C) suggest increased HXK and decreased TPI1 activity. Vein graft proteomics show HXK2 protein increased whereas TPI1 protein decreased in nondiseased veins (IVC and wild-type tissues) versus *Ldlr*^−/−^ vein grafts (neointimal + adventitial layers) (Figure XVIIIB and XVIIID in the Data Supplement). Other enzymes decreased in the neointima and adventitia but increased in the IVC tissues, and wild-type vein grafts were IDHG1 and IDH3A (Figure XVIIIA and XVIIIC in the Data Supplement). Isocitrate, an IDH substrate, decreased in M(LPS). Time course vein graft data parallel this finding because IDH3A and ACO2 are decreasing from day 1 to day 28 in the vein grafts (Figure XIXA in the Data Supplement), coinciding with worsening lesion burden, although unchanged in IVC samples (Figure XIXB in the Data Supplement). Thus, the regulatory network corroborates the above findings (Figure XX in the Data Supplement).

## Discussion

This study used a systems approach to profile vein graft lesion development, a major clinical problem promoted by maladaptive responses to changes from the venous flow to the arterial flow environment (“arterialization”). This platform, involving proteomics and network analysis, analyzed the proteome kinetics of experimental vein grafts in mice^16,17^ to increase understanding of vein graft disease. The specific goals were to establish a systems approach to identify potential targets for vein graft disease and verify this new platform via in vitro and in vivo studies involving loss-of-function/gain-of-function experiments. To accomplish the second goal, we chose the well-known PPARα pathway because it would be hard to support a new approach by examining the causal role of lesser-known pathways. In addition, we chose PPARα, for which specific drugs are available. Gain- and loss-of-function experiments substantiated our discovery platform by demonstrating that PPARα indeed exerts antiatherogenic and anti-inflammatory actions during vein graft lesion development. PPARα activation also attenuated lesion development in AVF, another vein maladaptation disorder. Multiple in vitro studies demonstrated PPARα modulation of macrophage metabolism, muting its inflammatory properties. The beneficial effects of PPARα may not necessarily depend on triglyceride lowering.^35–37^ A low dose of pemafibrate, a novel potent and selective PPARα modulator,^24^ suppressed vein graft lesion development. Metabolomic analyses revealed that pemafibrate may exert beneficial effects on vein grafts by modulating intracellular metabolism.

Although PPARα is a relatively known molecule, our findings on its role in vein grafts are novel. The present study successfully reports that our new platform identified previously unknown targets for vein graft disease and further provided new findings demonstrating that specific inhibition or activation indeed worsened or attenuated vein graft lesion development. We realize the value of the other targets, particularly SIRT6, which are subjects of our future studies.

Network analysis linked the mouse vein graft proteomics data with AVF disease, indicating similar mechanisms shared by these 2 “arterialized” vein diseases. The network closeness implied therapies for vein graft disease may also benefit AVF disease. In this regard, activation of PPARα, a target derived from vein graft proteomics, indeed reduced AVF lesion development.

Although tissue proteomics and network analysis provided these pathways and targets, an experimental limitation lies in the inherent tissue heterogeneity of mouse vein grafts. It is also complicated by minimal starting vein graft materials to attempt cell sorting before proteomics because of their small sizes. The varying proportions of macrophages and smooth muscle cells may be a concern for the skewed contribution of proteome origins. However, macrophage and smooth muscle cell content among syngeneic, diet-controlled, genetically modified proatherogenic *Ldlr*^−/−^ mice with postsurgical vascular disease modeling tend to have similar graft cellular compositions. Yet with the inability to correct for cell distribution during proteomic processing, we therefore resorted to network and pathway analysis to explore the possible significant source of the resulting vein graft proteome.

Metabolic and inflammatory pathways enriched in our in vivo analysis suggest macrophages being key agents in vein grafts. In vitro, the expected glycolytic slant and proinflammatory activation of M(LPS) macrophages^38^ were countered by PPARα activation.

LPS-stimulated macrophages exhibit a “blocked” mitochondrial IDH activity, causing citrate accumulation and escape to the cytoplasm.^31^ It results in increased inducible NOS transcription and reactive oxygen species generation.^39,40^ PPARα activation, through gene regulatory control of IDH, may relax the block, easing citrate to fuel into the TCA cycle.

PPARα activation increased both inducible NOS inhibitor ADMA and reactive oxygen species scavenger reduced-form of glutathione (GSH) in macrophages, mitigating oxidative damage.^32^ Consequently, lysophospholipids and ceramides, the reactive oxygen species breakdown byproducts of mitochondrial membrane phospholipids and sphingomyelins, are decreased, attenuating membrane potential depletion and “leakiness.”^41^ PPARα activation may thus promote mitochondrial membrane integrity and fluidity.

Our results augmented our understanding of vein graft disease, substantiated our target discovery platform, and provided molecular bases for the PPARα-targeted therapies for this major health burden.

## Acknowledgments

The authors thank Jung Choi, Peter Mattson, Alexander Mojcher, Jennifer Wen, Anna Ha, and Michael Creager for their technical assistance. We especially thank William Oldham, MD, PhD, for his assistance and guidance with the Seahorse metabolic assays. Some figures were created with BioRender.com. Author contributions: conceptualization, J.L.D. and M.A.; methodology, J.L.D., S.A.S., H.Z., J.W., E.A., and M.A.; investigation, J.L.D., H.Z., S.A.S., L.H.L., A.H., C.G.B., A.M., J.Q., S.C., J.T.M., and D.G.A.; writing—original draft, J.L.D.; writing—review and editing, M.A., E.A., P.L., S.A.S., L.H.L., A.H., C.S., J.T.M., and C.K.O.; funding acquisition, M.A.; resources, M.A.; supervision, M.A., S.A.S., A.S., and E.A.

## Sources of Funding

This study was supported by research grants from Kowa Company Ltd, Nagoya, Japan (to M.A.), and the National Institutes of Health (R01HL107550, R01HL126901, and R01HL149302 to M.A.).

## Disclosures

Outside the present study, M.A. has been supported by grants from Pfizer, Inc, and Sanofi US Services, Inc, on vein graft research. P.L. is an unpaid consultant to or involved in clinical trials for Amgen, AstraZeneca, Esperion Therapeutics, Ionis Pharmaceuticals, Kowa Pharmaceuticals, Novartis, Pfizer, Sanofi-Regeneron, and XBiotech, Inc. P.L. is a member of the scientific advisory board for Amgen, Corvidia Therapeutics, DalCor Pharmaceuticals, IFM Therapeutics, Kowa Pharmaceuticals, Olatec Therapeutics, Medimmune, Novartis, and XBiotech, Inc. P.L. serves on the board of XBiotech, Inc. The laboratory of P.L. has received research funding in the last 2 years from Novartis. P.L. has a financial interest in XBiotech, a company developing therapeutic human antibodies. His interests were reviewed and are managed by Brigham and Women’s Hospital and Partners HealthCare in accordance with their conflict of interest policies. The other authors report no conflicts.

## Supplemental Materials

Expanded Methods

Data Supplement Figures I–XIX

Data Supplement Tables I–V

References 42–46

## Supplementary Material


