# Supplementary material for: Systems Approach to Discovery of Therapeutic Targets for Vein Graft Disease: PPARα Pivotally Regulates Metabolism, Activation, and Heterogeneity of Macrophages and Lesion Development
Source: Circulation. 2021 Apr 6;143(25):2454–70. doi: 10.1161/CIRCULATIONAHA.119.043724 (PMC8212880; doi:10.1161/CIRCULATIONAHA.119.043724)
Supplement: Supplementary file 1 [file cir-143-2454-s001.pdf]

SUPPLEMENTAL MATERIAL

Systems Approach to Discovery of Therapeutic Targets for Vein Graft Disease: PPARα Pivotally Regulates Macrophage Metabolism, Activation, and Lesion Development

Julius L. Decano, MD[1], Sasha A. Singh, PhD[1], Cauê Gasparotto Bueno, MD[1], Lang Ho Lee, PhD[1], Arda Halu, PhD[1,2], Sarvesh Chelvanambi, PhD[1], Joan T. Matamalas, PhD[1], Hengmin Zhang, MD[1], Andrew K. Mlynarchik, BS[1], Jiao Qiao, BS[1], Amitabh Sharma, PhD[1,2], Shin Mukai, PhD[1], Jianguo Wang, PhD[1], Daniel G. Anderson, PhD[3], C. Keith Ozaki, MD[4], Peter Libby, MD[5], Elena Aikawa, MD, PhD[1,5,6], and Masanori Aikawa, MD, PhD[1,2,5,6]*

[1]Center for Interdisciplinary Cardiovascular Sciences, Cardiovascular Division, Department of Medicine, Brigham and Women's Hospital, Harvard Medical School, Boston, MA 02115, USA

[2]Channing Division of Network Medicine, Department of Medicine, Brigham and Women's Hospital, Harvard Medical School, Boston, MA 02115, USA

[3]Institutes for Medical Engineering and Science, Massachusetts Institute of Technology, Cambridge, MA, 02139, USA

[4]Division of Vascular and Endovascular Surgery, Department of Surgery, Brigham and Women's Hospital, Harvard Medical School, Boston, MA 02115, USA

[5]Center for Excellence in Vascular Biology, Brigham and Women's Hospital, Harvard Medical School, Boston, MA 02115, USA

EXPANDED METHODS

Materials, Specimen and Reagents Table

REAGENT OR RESOURCE	SOURCE	CATALOG #, RRID IDENTIFIER
**Antibodies**		
Anti-CD68 antibody	Abcam	ab53444, RRID:AB_869007
Goat anti-mouse IgG AF488	Invitrogen	A11017, RRID:AB_143160
Anti-a-SMA Cy3	Sigma	C6198, RRID:AB_476856
Anti-Mmp9- RPE	Sigma	SAB5200308-100UG, no RRID
Anti-Mmp13	Thermo Fisher	MA5-14247, RRID:AB_10985956
Donkey anti-rat IgG AF594	Thermo Fisher	A-11058, RRID:AB_2534105
**Chemicals, Peptides, Recombinant proteins, and Kits**		
Formalin	Thermo Fisher Scientific	SF96-20
Picrosirius red solution	VWR	KT037
Picric acid		
SHUR/Mount™ Liquid Mounting Medium, TBS®	VWR	15148-062
VECTASHIELD® Antifade Mounting Medium with DAPI	Vector Laboratories	H-1200
Harris Modified Method Hematoxylin Stains	Thermo Fisher Scientific	SH30-4D; SH30-500D
Eosin Y 1%	VWR	10143-130
Radioimmunoprecipitation assay buffer (RIPA) buffer	Cell Signaling Technologies	9806

Polyetheretherketone, P.E.E.K.; Extruded Sub-Lite-Wall® plastic tubing; 0.006+/-0.002;*;.004+/-.001;Natural;73 +2 -.000	Zeus Industrial Products, Inc.	OPN: 0000091922 (customized service catalog#)
Protease inhibitor cocktail	Sigma Aldrich	P8340-5ML
Isoflurane, USP 99.9%	Patterson Veterinary, Greeley CO	N/A
Buprenorphine HCl (Buprenex) 0.03 mg/mL in saline solution	Henry Schein Animal Health, Melville NY	N/A
Ethilon 7-0 sutures	Owens and Minor	1696G, P-1
Ethilon 8-0 sutures	Owens and Minor	280G, BV130-5
Ethilon 10-0 sutures	Owens and Minor	2870G, BV-75-3;
Ethilon 11-0 sutures	Owens and Minor	2881G, BV50-3;
Saline Solution 0.9% NaCl aqueous, sterile injection USP, 10 mL (Hospira)	Owens and Minor	18912
Hard tissue homogenizing CK28 beads in 2 mL tube	Bertin-instruments	P000911-LYSK0-A
Chloroform(Approx. 0.75% Ethanol as Preservative/Molecular Biology),	Thermo Fisher Scientific	BP1145-1
Methanol, Optima™ LC/MS Grade, Fisher Chemical	Thermo Fisher Scientific	A456-1
Urea	Sigma Aldrich	U4884-500G
Thiourea ReagentPlus®, ≥99.0%	Sigma Aldrich	T7875-500G
UltraPure™ 1M Tris-HCI, pH 8.0	Thermo Fisher Scientific	15568025
Pierce™ BCA Protein Assay Kit	Thermo Fisher Scientific	23225
Triethylammonium bicarbonate	Sigma Aldrich	17902-100ML
RapiGest SF	Waters Technologies Corporation	186001861
Tris(2-carboxyethyl)-phosphine hydrochloride	Sigma -Aldrich	77720
Iodoacetamide	Thermo Fisher Scientific	A39271
Lysyl Endopeptidase	Fujifilm Wako	125-05061
Fisher chemical Water, Optima™ LC/MSGrade	Thermo Fisher Scientific	W6-1

Trifluoroacetic acid for HPLC, ≥99.0%	Sigma-Aldrich	302031-10X1ML
OASIS® HLB extraction cartridge	Waters Technologies Corporation	186000383
Acetonitrile	Thermo Fisher Scientific	A955-1
Formic Acid	Thermo Fisher Scientific	28905
Lipopolysaccharides from Escherichia coli O111:B4, LPS	Sigma- Aldrich	L4391
Dimethyl sulfoxide, DMSO, molecular biology grade	Sigma-Aldrich	D8418-100ML
Isotonic sodium chloride solution	Mountainside Medical Equipment	H04888-10
Macrophage colony stimulating factor, M-CSF	Peprotech Inc.	300-25
Dulbecco's modified eagle's medium	Thermo Fisher Scientific	10569010
Fetal calf serum	Thermo Fisher Scientific	97068-085
Penicillin and streptomycin	VWR	45000-652
PerfeCTa® qPCR SuperMix	VWR	95050-100
Magnetofection SilenceMag siRNA Delivery Reagent (1,000ul)	OZ Biosciences	SM11000
Lymphocyte separation media	VWR	IC50494
RPMI 1640 with L-glutamine	VWR	12001-590
Human serum	Gemini Bio-Products	100-512
EasySep™ Human Monocyte Enrichment Kit without CD16 Depletion	StemCell Technologies	19058
LabAssay Triglyceride	Fujifilm Wako	290-63701
Cholesterol E Assay	Fujifilm Wako	439-17501
Autokit Glucose	Fujifilm Wako	997-03001
Tetramethylrhodamine, Methyl Ester, Perchlorate (TMRM)	Thermo Fisher Scientific	T668
MitoSOX™ Red Mitochondrial Superoxide Indicator	Thermo Fisher Scientific	M36008
Accutase Cell detachment solution	BD Biosciences	561527

**Biological Samples**		
LeukoPak quarter packs	StemCell Technologies	70500.2
Unpurified buffy coat	Research Blood Components	N/A
RAW 264.7 (ATCC® TIB-71™), mouse macrophage-like cell line	ATCC	RRID:CVCL_0493
**Experimental models and animal diet**		
Clinton/Cybulsky High Fat Rodent Diet with Regular Casein and 1.25% Added Cholesterol	Research Diets	D12108Ci
Rodent Diet with 40 kcal% Fat, 1.25% Cholesterol, and 3.6 mg/kg pemafibrate	Research Diets	D14101401i
C57BL/6J	The Jackson Laboratory	000664, RRID:IMSR_JAX:000664
B6.129S7-Ldlrtm1Her/J	The Jackson Laboratory	002207, RRID:IMSR_JAX:002207
**Oligonucleotides**		
ON-TARGETplus siPeroxisome proliferator activation receptor alpha (mouse), siPPARα	Dharmacon	L-040740-01-0005
ON-TARGETplus Non-targeting Control siRNAs, siControl	Dharmacon	D-001810-01-05
ON-TARGETplus siPeroxisome proliferator activation receptor alpha (human), siPPARα	Dharmacon	L-003434-00-0005
**Software**		
Qlucore	https://qlucore.com/	
MetaCore	https://portal.genego.com/	RRID:SCR_008125

Prism 7 (GraphPad)	https://www.graphpad.com/scientific-software/prism/<br><br>RRID:SCR_002798
XINA	https://github.com/cics-bwh/XINA
Gephi	https://gephi.org/ RRID:SCR_004293
Cytoscape	https://cytoscape.org/download.html RRID:SCR_003032
Networkx	https://networkx.github.io/ RRID:SCR_016864
Venny 2.1	http://bioinfogp.cnb.csic.es/tools/venny/index.html<br><br>RRID:SCR_016561

CONTACT FOR REAGENT AND RESOURCE SHARING

Further information and requests for resources and reagents should be directed to the lead contact Masanori Aikawa (MAIKAWA@BWH.HARVARD.EDU)

METHODS DETAILS

Developing the vein graft animal model.

The Institutional Animal Care and Use Committee at Beth Israel Deaconess Medical Center (protocol # 021-2017) reviewed and approved all animal experimental protocols (protocol # 021-2017). Male wild type and male LDL receptor–deficient (Ldlr-/-) mice in a C57BL/6 background were purchased from Jackson Laboratory (Bar Harbor, ME) and maintained with normal chow diet and water ad libitum. 12-week-old Ldlr-/- male mice were switched to high fat diet containing 1.25% cholesterol (D12108CO; Research Diets, NJ) that was started one week prior to vein implantation and continued until four weeks post-surgery. Wild type mice were maintained on normal chow throughout the same time span before and after surgery.

After one week high-fat diet feeding for Ldlr-/- (normal chow for wild type), vein graft implantation was performed as previously reported [1]. An approximately 4.5 mm segment of the supra diaphragmatic segment of the inferior vena cava (IVC) was harvested from age- and genetic strain-matched donor mice (also pre-fed for one week on high fat diet for Ldlr-/- strains) and implanted into the recipient's left mid-common carotid artery using small cuffs made from polyetheretherketone, P.E.E.K., tubing (Zeus, Orangeburg, SC) and tied securely with Ethilon 10-0 sutures. Wound closure was done with interrupted sutures (Ethilon 7-0). Post-surgery routine care and pain medication were given (Buprenorphine 0.05 μg/gram body weight/dose) for 2 doses per day for 3 days. After vein graft implantation, blood flow and pulsation of the graft was visually assessed to confirm successful grafting and monitored weekly using high resolution vascular ultrasound imaging with the Vevo 2100 (Fujifilm Visualsonics) until four weeks post vein graft

implantation. Neointima plaque was estimated using wall thickness measurement tool from the VevoVasc™ analysis package of the VevoLab™ software version 1.6.0 build 6078 (Fujifilm Visualsonics), in order to choose the appropriate animal model for the study.

Vein graft vessel wall layer proteomics sample preparation

Vein graft implantation surgery.

Vein graft implantation was done at 12 weeks of age on two Ldlr-/- mice and two wild type C57BL6 mice using same strain vein donors. After four weeks of vein graft maturation, vein grafts were imaged by high resolution ultrasonography (Vevo 2100, Visual Sonics) to monitor development of the neointimal plaque. After imaging, animals were euthanized, bled and perfused through the left ventricle with cold phosphate buffered saline (PBS) to clear intravascular space of remaining blood cells. Approximately 4.5 mm length of supradiaphragmatic inferior vena cava (IVC) for all mice were harvested (along with the vein grafts at the carotid area) as non-arterialized controls for each of the animal vein graft (VG) samples. All IVC's were minced in 400 µL cold solution of radioimmunoprecipitation assay buffer (RIPA) buffer (Cell Signaling Technologies, Danvers, MA) with protease inhibitor cocktail, PIC, (Sigma) and processed for mechanical tissue disruption using Precellys beads (Precellys, Atkinson, NH) high speed multi-directional dual bead-grinding homogenization. Vein grafts of Ldlr-/- mice were isolated from their carotid attachments and dissected free of the P.E.E.K. cuffs and perivascular fat as much as possible. Thickened neointimal plaque (NEO) was micro-dissected under the microscope to cleanly separate it from the adventitial layer (ADV) of the graft. The NEO and ADV layers were immediately placed in separate containers with 400 µL RIPA buffer with PIC as above and minced using fine microdissecting scissors. Vein grafts of wild type mice (WVG) did not produce a visibly thickened plaque both by ultrasound imaging and by ocular inspection making vessel layer microdissection not feasible. Hence, they were cleaned of their minimal perivascular fat, harvested and minced

as above without separating neointima and adventitia. All vein graft tissue/tissue layer samples were processed for mechanical disruption and lysis using Precellys system as with the IVC controls above.

Protein and peptide preparation.

Tissue homogenates were sonicated on ice in a 4°C environment/room using Sonifire 450 (Branson, CT) four times for 15 seconds at constant duty cycle and power output of 2.0in 30-second intervals. Protein disk precipitate from the sonicated homogenate was extracted using a 2:1 chloroform: methanol solution through vigorous vortex mixing for 30 seconds followed by high speed centrifugation at 18,000g for 30 minutes at 4°C. Upper and lower liquid phases were discarded and each protein disk were solubilized in 100 µL of 6M urea/2M thiourea/25 mM Tris (pH 8.0) solution in a 37°C heated tube mixing shaker (700 rpm, Thermomixer; Thermo Fisher, Cambridge, MA) for 60 minutes. After complete dissolution, protein amount in the lysate was quantified using BCA protein quantitation assay (Thermo Fisher Scientific, Cambridge, MA). Across the board, 50 µg of protein samples were digested in-solution with 0.11 M triethylammonium bicarbonate, TEAB (Sigma, Burlington, MA), 0.11% w/v RapiGest SF surfactant (Waters, Milford, MA) and 55.56 mM tris(2-carboxyethyl)-phosphine hydrochloride, TCEP (Sigma, Burlington, MA) mixture in 56°C heated shaker at 800 rpm for 45 minutes. Free cysteine residues were alkylated by adding iodoacetamide (IAA) to a final concentration of 30 mM and then incubated in the dark for 30 minutes. Subsequently, peptide digestion was carried out overnight in 37°C using 0.5 µg Lysyl endopeptidase endoproteinase enzyme (Wako, Richmond, VA) per 50 µg sample protein input.

After peptide digestion, RapiGest reagent was removed by repeated dilution with LC/MS grade water followed by evaporative vacuum centrifugation for a total of five cycles, while being careful to avoid complete drying-out of the samples. After which, sample volume was raised to 1.4 mL volume with LC/MS grade water (Thermo Fisher, Cambridge, MA). Then, samples were

acidified on ice by adding trifluoroacetic acid, TFA (Sigma, Burlington, MA), to a final concentration of 10% and incubated at 37°C (shaker/mixing) for 30 minutes, followed by centrifugation at 14,000 rpm for 20 minutes to collect peptide supernatant solution and to discard the RapiGest® pellet. Samples are then desalted and eluted using 1.0 mL (10 mg) OASIS® HLB extraction cartridge columns (Waters, Milford, MA) as per manufacturer's instructions. Eluted peptide samples were completely dried by vacuum centrifugation using Vacufuge (Eppendorf, Hauppauge, NY) then resuspended in 42 µL of loading buffer (5% acetonitrile, 5% formic acid). Samples were further diluted 10-fold for mass spectrometry.

Vein graft time course proteomics.

Twelve Ldlr-/- mice underwent *vein graft implantation* as above. Each mouse was maintained on high triglyceride high cholesterol Clinton-Cybulsky diet (Research Diets, New Brunswick, NJ) one week prior to implantation and continued until designated time points for vein graft harvest. The four timepoints investigated were day 1, day 3, day 7 and day 28 post-vein graft implantation. Three mice for each time point were maintained on high fat diet and then were euthanized for vein graft (VG) and inferior vena cava (IVC) harvest as described above. Protein and peptide preparations steps were done as above with the following exceptions: the protein input was increased to 100 µg and Lysyl endopeptidase was used for proteolysis. The samples were diluted between 2 to 10-fold in order to acquire a constant total ion current.

Mass spectrometry and proteomics data analysis

Peptide samples were analyzed in duplicate with the high resolution/accuracy LTQ-Orbitrap (Elite model) mass spectrometer fronted with a Nanospray FLEX ion source and coupled to an Easy-nLC1000 HPLC pump (Thermo Scientific). The peptides were subjected to a dual column set-up: an Acclaim PepMap RSLC C18 trap column, 75 µm X 20 mm; and an analytical

column which was packed with 3 µm, 100 Å pore size C18 resin (Bruker) in a 75 µm X 200 mm long piece of fused silica capillary in-house. For the vein graft vessel layer samples, the analytical gradient was run at 350 nl/min from 15 to 28 % Solvent B (acetonitrile/0.1 % formic acid) for 60 minutes, followed by five minutes of 95 % Solvent B. Solvent A was 0.1 % formic acid. For the vein graft time course samples, the gradient was 10 to 28 % Solvent B for 120 minutes. All reagents were LC/MS-grade. The instrument was set at 120 K resolution, and the top 20 precursor ions (within a scan range of 380-2000 m/z) were subjected to collision induced dissociation (collision energy 35%) for peptide sequencing (MS/MS).

All MS/MS data were queried against the mouse UniProt database (downloaded on August 01, 2014) using the HT-SEQUEST search algorithm, via the Proteome Discoverer (PD) Package (version 1.4, Thermo Scientific). Methionine oxidation was set as a variable modification and carbamidomethylation of cysteine residues was set as a fixed modification. Peptides were filtered based on a 1 % FDR [43]. Peptides assigned to a given protein group (Master protein), and not present in any other protein group, were considered as unique and used for quantification. A minimum of two unique peptides were included for each dataset. For both vein graft proteomics studies, vessel wall layer and time course, we used the peptide (MS1)-based, area-under-the-curve (AUC) method for label-free-based quantification of proteins using up to the three most abundant peptides per protein, as provided by PD 1.4.

The proteins from each PD-exported dataset were normalized by the protein median AUC [15]. The protein abundance trends (heat maps and principle component analysis) were analyzed using Qlucore Omics Explorer 3.2 (Qlucore AB, Lund, Sweden). Using R, we applied a generalized linear model (y ~ time * group, where time is each time point and group represents IVC control or VG surgery group) to find differentially regulated proteins between two groups. The Welch's *t*-test was used for the mouse static data (ADV and NEO + WT VG #1 vs. IVC + WT VG#2, with minimal plaque).

For the time course proteomic analysis, normalized protein abundances from the different time point conditions, from vein graft tissues and IVC specimens were processed using XINA, a Bioconductor package in R that was previously published by our laboratory [47]. Each timepoint comprises of three biological replicates (post VG surgery mice) of vein graft tissues and matching same-animal in situ IVC control. Proteins were filtered out if they are not sequenced in all of the sample-conditions and if the protein only has less than 2 unique peptides sequenced. Abundances per sample conditions are averaged and these averaged values are the data introduced to the XINA analysis. XINA allows for integrated multiplexed proteomic kinetic data analysis of our different sample-conditions (i.e. vein graft day 1, IVC day 1, etc.) that demonstrates relative proteome abundance trends across time. By default, XINA implements model-based clustering to classify proteins depending on their abundance profiles. Sum-normalization method was used to standardize protein abundances across all sample-conditions to range from 0 to 1. Proteins are then clustered together into specific groups or "cluster numbers" based on the similarity of their trend lines. For clustering, the model-based algorithm either optimizes the number of clusters at a minimum Bayesian information criteria (BIC) in an unbiased manner. Clustering is performed using the "mclust" R package, which determines a covariance structure and the number of clusters at the best BIC, which in our case resulted in 30 clusters (**Figure 1F**). XINA provided statistical and graphical tools to investigate the coabundance patterns of proteins derived from this multiplexed (VG and IVC) time-series proteomics data. Further explanation of how the algorithm clusters the protein abundances can be found in the aforementioned methods report [6]. Trends may either be increasing, decreasing, fluctuating or relatively unchanging across the four time points (day 1, day 3, day 14, and day 28). We subdivided the trend patterns into two: early phase abundant or predominant proteins and late phase abundant or predominant proteins. Among these proteins found in these trend clusters, we identified proteins that have higher abundances in either day 1 or day 3 time point, relative to their abundances in day 14 and day 28 time points using a reference threshold (red dashed line in legend, **Figure 1F**) and classify

these proteins as early phase predominant proteins (see purple trend lines, **Figure 1F)**. On the other hand, protein trend clusters with higher abundances in either day 14 or day 28 time points are classified as late phase predominant proteins (see orange trend lines, **Figure 1F**). Of these proteins we exclude the ones that have either early phase or late phase trend patterns that are also found in the IVC samples. Proteins exhibiting early phase and late phase patterns found in vein graft samples alone, but have unchanging trend (gray trend lines, **Figure 1F**) found in IVC (control) samples, are considered for the analysis. The rationale behind this is that if proteins have the same trend in both vein graft samples and IVC non-arterialized or control samples, then these proteins may have little to no association with the arterialized phenotype of the vein. We also excluded proteins that have unchanging trend (clusters with gray trend lines) in our downstream analysis.

Using these statistically filtered data, we performed integrated networks-based analysis including pathways enrichment to find significant disease associations and potential therapeutic target(s) among 'hub' nodes using the proprietary computational algorithms of MetaCore™ (Clarivate Analytics/Thompson Reuters).

Target prioritization by network of pathways analysis.

Increasing differentially expressed protein (DEP) lists from each group (Grp 1: IVC, Grp 2: NEO/ADV, Grp 3: Early time point, and Grp 4: Late time point) were used separately as input proteins/gene list for pathways enrichment to MetaCore™. MetaCore™ is a network and pathways enrichment web application using network interactome curated by Clarivate Analytics (and Thompson Reuters). For each group we enriched for biological pathways and exported this list of pathways, including the corresponding sample group DEPs associated for each pathway, as CSV (comma separated values) files. We then constructed a network for each sample group where the pathways are designated as nodes. Each pair of nodes is connected by an edge if these

nodes (pathways) share at least one common protein. The edge thickness is proportional to the number of shared proteins between these nodes. The node sizes are proportional to the number of proteins in that pathway (node) that are present from the proteomics-derived DEP list. After the networks were constructed using python scripts-Networkx package (https://networkx.github.io/), network topology was analyzed using Cytoscape (https://cytoscape.org/download.html), while final network data visualization was done using Gephi (https://gephi.org/). Measure of centrality were taken such as betweenness centrality, degree count, and closeness centrality [44]. Using these centrality measures we ranked each pathway from each group network. We then took the top three most central pathways (hubs) of each sample group network (n=4) and explored them for their most central "hub" proteins using betweenness centrality measure of the proteins comprising each top-ranked pathway (top three pathways only). The three most central nodes (proteins) in each top-ranked pathway (top 3 per sample group network) are compiled as a list of 12 potential targets for vein graft disease.

Formulation of siRNA lipid nanoparticles (LNPs) for in vivo gene expression silencing

siRNA for firefly luciferase (control siRNA) and mouse PPARα were synthesized by Axolabs (Germany). We used 2 oligonucleotides XD-07623 and XD-07624 among a list of 10 candidate siRNA oligonucleotides listed in **Supp Table 6** after rigorous optimization of silencing efficacy (data not shown). Formulation of C12-200 lipid nanoparticles conjugated with siRNA targeting mouse PPARα, and non-targeting control siRNA (modified to avoid immune stimulation and off-target effects) were synthesized similarly as previously described [45]. Macrophage-targeted lipid nanoparticles were prepared and encapsulated with siRNA. In detail, lipid-like material C12-200 was combined with siRNA (whether PPARα siRNA or control (firefly luciferase) siRNA) using a spontaneous vesicle formation technique [45]. After nanoparticle assembly, particle size was

characterized using dynamic light scattering. Both control siRNA- and PPARα siRNA-LNPs had an average diameter of 97.6 nm.

In vivo loss-of-function study by using PPARα silencing RNA-lipid nanoparticles (LNP) complexes

Loss-of-function in vivo study of silencing PPARα was carried out in a blind fashion with the use of macrophage specific lipid nanoparticle (LNP) incorporating PPARα siRNA. In this dosage study, 0.25 mg/kg BW/dose of siRNA-LNPs were injected intravenously via tail vein of study mice. 40 16-week-old Ldlr-/- mice maintained on high fat high cholesterol diet for one week prior to undergoing vein graft surgery. Twenty mice served as VG donors to the other 20 "recipient" mice. At the day of surgery, VG recipient animals were assigned randomly to receive either the siControl group or siPPAR-α group depending on the type of siRNA-LNP will be given. Both trial analyst and surgeon were blinded to the groupings as assigned by a third party. Tail vein injections were given a week before surgery, right after surgery, and twice a week for the subsequent four weeks following VG surgery for a total of 11 doses. After 4 weeks, vascular ultrasound was done to assess vein graft plaque sizes. In a select set of animals, we tested for glucose uptake in the vein grafts using intravenously injected RediJect Glucose 24 hours prior to animal tissue harvesting. Glucose uptake is recorded by near-infrared fluorescence imaging using the Olympus MVX-10 macroview scope.

In vivo gain-of-function vein graft study through PPARα activation using highly selective PPARα agonist pemafibrate

A highly selective PPARα agonist pemafibrate (Kowa, Inc.) was used as PPARα activator given throughout the diet to dyslipidemic Ldlr-/- mice with vein graft implants to assess whether vein graft plaque lesion growth will be mitigated. Pemafibrate was admixed into the Clinton-

Cybulsky high fat diet for a final dose of 0.2 mg/kg body weight/day pemafibrate. 44 mice were used in the study, 22 of which are used as donor mice to provide supra-hepatic inferior vena cava vascular segment for the vein grafting. Each donor-recipient mouse match was determined by pairing mice with closely similar body weights in which the slightly heavier mouse becomes the donor. Twenty two pairs of donor-recipients were randomly assigned in an unbiased fashion (by a "third party" investigator) to either a control group (no pemafibrate treatment) or to the pemafibrate drug treated group. Experimenters and the surgeon were group-agnostic to the drug group assignments. Both donor and recipient mice were pre-fed with high fat diet with or without pemafibrate admixed into the diet for two weeks before undergoing vein graft surgery. After the surgery, assigned diets are maintained for each drug group up to 4 weeks post-surgery. Weekly ultrasound imaging monitoring the development of vein graft lesions and plaque post-implantation. After the 4th week time point, mice were sacrificed. 24 hours prior to sacrifice, mice were injected intravenously with MMPSense 680 tracer (peak emission at 680 nm) to assess protease activation in the vein grafts. At the point of sacrifice, blood and plasma samples were collected for plasma lipid assessments. After, PBS perfusion, vein grafts in situ were imaged via near infrared fluorescence using the Olympus MX10 macroview scope set at fluorescence scanning with the Cy5 filter cube set (~670-649 nm band pass filter set) to detect MMPSense substrate activation which approximates tissue protease activation. Vein grafts were harvested for histologic examination.

Near infrared imaging fluorescence of protease activation in vein grafts.

At four weeks post implantation, vein grafts have at various degrees developed intraluminal neointimal plaques. Macrophage accumulation in the neointima may be a source of proteases that eventually may affect local tissue remodeling influencing collagen fibrils integrity and consequently plaque stability. To assess protease activation as close to the in vivo condition

as possible we used MMPSense 680 (Perkin Elmer, Waltham, MA). MMPSense is a substrate activatable fluorescence sensor for presence of active proteases. AT 24 hours prior to scheduled animal sacrifice, mice with 4-week-old vein grafts were injected intravenously with 100 uL of 20 nmol/1.5 mL concentration of MMPSense (reconstituted in sterile saline solution). After 24 hours, in situ vein grafts were imaged by sufficiently surgically exposing the left paratracheal area of the mouse's neck and imaging using the Olympus MVX-10 Macroview scope set at fluorescence scanning mode using the Cy5 filter cube set. All exposure times, and image capture settings (gain, etc.) were kept constant across all animals. Relative fluorescence intensities were quantified using Image J software.

Double fluorescence immunohistochemistry of vein grafts

Mouse vein grafts were prepared as fresh frozen tissue using OCT (optimal cutting temperature) resin. Frozen tissue blocks are transversely sectioned as 7.0 micrometer thick sections onto Superfrost^{TM} plus-plus glass slides. The sectioned slides were thawed and dried at room temperature for 30 minutes followed by fixation with 4% paraformaldehyde solution in phosphate buffered saline (PBS) for 15 minutes. After rinsing the fixative solution off the slides with three washes of PBS, the slides were blocked with 5% goat serum and 5% donkey serum in PBS solution (blocker solution) for 30 minutes before exposing to the primary antibody solution. The primary antibodies that were used (e.g. CD68, α-SMA, MMP-9, & MMP13) were diluted with a solution of 1% bovine serum albumin (BSA) in phosphate buffered saline (PBS) at final concentration of 1.0 ug/mL. Primary antibody incubation was done overnight at 4°C followed by triple washes of the slide sections with room temperature PBS at 5 minutes each. Subsequently, the slides were incubated with a secondary antibody solution of fluorescent conjugated antibodies diluted at 1:500 ratio in 1% BSA solution (PBS) for 1 hour. Slides were washed in PBS three times for 5 minutes in room temperature. Each slide was then mounted using fluorescent mounting

medium VECTASHIELD with DAPI (Vector Laboratories, Burlingame, CA). Imaging was done on a Nikon Ti Eclipse confocal microscope. Image quantification was done by using Nikon Elements and % positive area of the signal was quantified within the neointimal area.

Picrosirius staining of vein graft sections

All succeeding steps in this workflow were carried out in room temperature ($25^{O}C$). Vein graft sections post-cryostat sectioning are left to dry for 30 minutes and were then fixed in 10% formalin solution (Fisher Scientific, Pittsburg, PA) for 10 minutes. After five minutes of rinsing in tap water, slides were washed with distilled deionized water (ddH_2O) twice for three minutes each time. The slides were then incubated with 0.1% Picrosirius red (VWR, Visalia, CA) at pH 2.0 for 90 minutes. Slides were then rinsed with 0.01 N HCl, twice for two minutes, followed by ddH_2O quick rinse. Slide sections were then serially dehydrated with increasing concentrations of ethanol (70%, 95%, 100%) then incubated in 100% Xylene for three minutes and coverslip mounted using SHUR/Mount™ mounting media (VWR, Visalia, CA). Fibrillar collagens shows bright red and green birefringence under polarized light. Red-appearing collagen fibers are typically mature and stable collagen fibers while the green collagen fibers are typically fragmented or immature collagen fibers.

Hematoxylin and Eosin Staining

All succeeding steps in this workflow were carried out in room temperature ($25^{O}C$). Vein graft sections post-cryostat sectioning are left to dry for 30 minutes and were then fixed in 10% formalin solution (Fisher Scientific, Pittsburg, PA) for ten minutes. After five minutes of rinsing in tap water, slides were washed with distilled deionized water (ddH_2O) twice for three minutes each. Slides are then placed in a solution of Harris hematoxylin (Fisher Scientific, Pittsburg, PA) (prefiltered prior to use) for two minutes. Slides were then rinsed well in ddH_2O. The slides were

then de-stained with 0.5% acetic acid quickly by dipping each slide in the acidic solution 2-3 times immediately followed by a rinsing step of ddH_2O. Slides are then incubated in “bluing” solution of ammonium water (0.2% ammonium hydroxide in water) for one minute. This bluing reagent is rinsed off with running tap water for five minutes. Subsequently, the slides are briefly rinsed in 70% ethanol followed by staining with 1% alcoholic eosin (Eosin Y, VWR, Visalia, CA) for 25 seconds with gentle agitation of the slides. Eosin stain is washed off by dipping the slides in 2 fresh washes of 95% ethanol solution. Slides are then dehydrated with 100% ethanol followed by 2 washes of xylene for 3 minutes. Slides are the mounted using SHUR/Mount™ mounting media (VWR, Visalia, CA).

Plasma lipid and glucose colorimetric assay

Venous blood was harvested from the animals under terminal sedation using heparinized phlebotomy needles. Blood samples underwent centrifugation at 700 g for 20 minutes to separate platelet poor plasma. Plasma samples were assayed for triglyceride levels, cholesterol levels and glucose levels. We used the LabAssay Triglyceride Assay Kit (Wako, Richmond, VA) to assay plasma triglycerides, the Cholesterol E Assay (Wako, Richmond, VA) to measure plasma total cholesterol, and the Autokit Glucose Assay (Wako, Richmond, VA) to measure plasma random glucose levels. All procedures for these colorimetric assays were done as per manufacturers' suggested protocols. Colorimetric absorbance measurements were done using the SpectraMax Gemini XPS (Molecular Devices, San Jose, CA).

Bone marrow derived mouse macrophage culture

Wild type C57BL/6 mice aged ~12 weeks were sacrificed (n=6) to harvest both right and left femurs. Both ends of the femurs were cut at the metaphysis region and the bone marrow was flushed with 1 ml sterile 0.9% sodium chloride saline solution (Mountainside Medical Equipment,

Marcy, NY) twice through a 40-micrometer cell strainer (BD Biosciences, San Jose, CA). The bone marrow filtrate was centrifuged (1500 rpm for ten minutes) to pellet the bone marrow cells from the saline solution. Cells were counted then resuspended in growth media: Dulbecco's modified Eagle's media, DMEM (Thermo Fisher, Cambridge, MA) supplemented with 10% fetal calf serum, 1% penicillin-streptomycin and macrophage colony stimulating factor, M-CSF (Peprotech, Rocky Hill, NJ) at a final concentration of 50 ng/mL. Resuspended cells are then plated at a concentration of 2.5 x 10^5 cells per cm^2 growth area of 6-well culture dishes with media replacement every other day. At day 5 in culture, M-CSF is then withheld from the growth media. At day 10 in culture, cells are used for functional experiments.

In vitro mRNA silencing experiments and real time quantitative PCR assay (RT-qPCR)

Silencing experiments with PPARα are carried out using magnetofection of siRNA of PPARα or a non-targeting siRNA (siControl) using SilenceMag transfection kit (OZ Biotechnology, San Diego CA). Protocols for silencing were based on manufacturer's recommendations and was described in a previous report [17]. We silenced PPARα expression using 40 nM of species specific PPARα siRNA SMART Pool ON-TARGET oligonucleotide or Non-targeting siRNA (Dharmacon, Lafayette, CO). To check for PPARα specificity of differential gene expression, we added pemafibrate 100 nM to either non-targeting control or PPARα silenced groups. For each silencing condition used, we nested the following additional conditions: (1) PBS for 6 hours for baseline macrophage status (plus 5 ppm dimethyl sulfoxide, DMSO), (2) lipopolysaccharide (Sigma-Aldrich, Burlington, MA), LPS, at 10ng/mL for 6 hours + DMSO (5 ppm), (3) LPS at 10ng/mL for 6 hours with 100 nM pemafibrate (Kowa, Tokyo). RT-qPCR was carried out using PerfeCTa® qPCR SuperMix kit (VWR, Visalia, CA) as per kit vendor's recommended protocols.

Peripheral blood mononuclear cell (PBMC) cell culture

Peripheral blood mononuclear cells (PBMCs) were derived from mononuclear cell preparations of commercially available buffy coats (Research Blood Products, Boston, MA) using Lymphocyte Separation Medium, LSM (Thermo Fisher, Cambridge, MA) as per manufacturer's recommendations. Human peripheral blood mononuclear cells (PBMCs) from buffy coats were maintained *in vitro* on 5% human serum in RPMI1640 underwent macrophage differentiation. The company recruited donors under a New England Institutional Review Board– approved protocol for the Collection of White Blood for Research Purposes (NEIRB#04–144). PBMCs were resuspended at a concentration of 5.0 x 10^6 cells/ mL in serum free RPMI 1640 basal media (Thermo Fisher, Cambridge, MA) and allowed to attach onto the cell culture surface of 6-well cell culture plates for one hour; after which, the original serum free media containing non-attached cells were discarded and replaced with fresh growth media, RPMI 1640 (Thermo Fisher, Cambridge, MA) with 5% human serum (Gemini Bioproducts, Sacramento, CA). Human peripheral blood mononuclear cells (PBMCs) from buffy coats were maintained *in vitro* on 5% human serum in RPMI1640 underwent macrophage differentiation after 10 days. Mouse primary macrophages were derived from bone marrow cells of normal wild type 8-10 week old male mice and cultured in DMEM with 10% fetal calf serum and 50 ng/mL M-CSF (Peprotech, New Jersey). Cells were cultured for seven days with regular growth media change intervals of 2-3 days and allowed to fully differentiate into macrophages. At day 8 post-seeding, cells were stimulated with either of the following stimulations: (1) PBS for six hours for baseline macrophage status (plus 5 ppm DMSO), (2) lipopolysaccharide (Sigma-Aldrich, Burlington, MA), LPS, at 10ng/mL for 6 hours + DMSO (5 ppm), (3) LPS at 10ng/mL for six hours with 100 nM pemafibrate (Kowa, Tokyo). Samples were then processed for single cell qPCR experiments. All experiments were carried out in biological triplicates using three buffy coat donors with PBMC isolations done at different occasions depending on the schedule of blood collections.

Single cell qPCR and analysis

Monocytes are isolated from peripheral blood mononuclear cells derived from buffy coats (Research Blood Products, Boston, MA) using EasySep human monocyte negative selection kit (STEMCELL Technologies, Cambridge, MA). Following manufacturer's recommended protocol, monocytes are isolated and plated for cell culture using serum free RPMI 1640 for 2 hours. Subsequently, serum free media was discarded along with floating non-adherent monocytes and was replaced with complete growth media: RPMI 1640 with 5% human serum and supplemented with penicillin streptomycin. Cells were maintained in this culture media for ten days to allow cells into fully differentiating into adherent macrophages. Cells were divided into three conditions: (1) baseline non-stimulate macrophages, pre-treated with 5 ppm DMSO for 12 hours, (2) pre-treated macrophages with 5 ppm DMSO for 12 hours then stimulated with 10 ng/mL LPS treatment for 6 hours and (3) pre-treated macrophages with 100 nM pemafibrate for 12 hours then stimulated with 10 ng/mL LPS treatment for 6 hours with cotreatment of 100 nM pemafibrate. Cells were detached from the cell culture dish by incubating cells in Accutase (BD Biosciences, San Jose, CA) for 10 minutes in room temperature followed by gentle cell scraping. Cells are then washed once with PBS containing 1% bovine serum albumin (BSA). Cells were resuspended in Live/Dead Cell cocktail solution (Thermo Fisher, Cambridge, MA) and incubated for 10 minutes for viability staining. Cells were then washed once with PBS with 1% BSA and then sorted in a flow sorter, BD FACS Aria II SORP to isolate viable cells. Sorted cells were then washed and resuspended in 3% BSA in PBS at a concentration of 250,000 cells/mL. The resulting cell suspension was applied into a pre-primed Fluidigm C1 IFC plate and sorted in a C1 sorter (Fluidigm, San Francisco, CA) to isolate single cells for lysis, RNA harvest and cDNA synthesis. All procedures for C1 sorting were done as per manufacturer's instructions. The resulting single cell cDNA were harvested and processed for single cell qPCR using HD BioMark (Fluidigm, San Francisco, CA). Procedures for single cell qPCR using the HD BioMark were performed as per vendor's (Fluidigm's) recommended protocol. Using Fluidigm Real-Time PCR Analysis software, Ct

thresholds were analyzed using the following settings: quality threshold at 0.65, baseline correction was linear, and the Ct threshold method used was “Auto(Detectors)”. Cells expressing undetectable signals for GAPDH or RPLP0 were eliminated from the analysis. Raw qPCR results were normalized using GAPDH. For genes that had undetected values (C_t = 999), we substitute those values with 999 in the calculation of -ΔΔCt and consider the cell to have undetectable expression for this gene. To simplify summarizing the expression profile of every cell in this experiment, we used viSNE visualization (Cytobank) to describe how gene expression changes from cell to cell, condition-to-condition. To do this, we converted the gene expression data matrix (cell ID x Gene assay) for each donor with each condition into FCS files using FlowJo software version 10.4. To do this, we multiplied all data points by 10,000 and adjusted the axes of each previewed FCS plot for each donor-condition sample set using Arcsinh scales. We then exported this into FCS v3.0 files and uploaded to Cytobank for further analyses. To do the viSNE analysis (tSNE) the following criteria are used: (1) Genes (channels) were selected to separate cellular subsets of interest are all the genes that showed marked heterogeneity in expression levels across the cells (32 genes), see table below.

TaqMan Gene Expression primers used

BMAL1	ACACA	IL1B	NLRP3
CCL2	CXCL11	IL4R	ADIPOR1
CCL8	CXCL5	IL6	PPARA126BP
CD29	CXCL6	JAK1	REVERBa
CD36	CXCL9	JAK2	STAT1
CD44	CYTB	JAK3	STAT6
CLOCK	ACADM	NFKB	TNF
CPT1A	IL10	NLRP1	RPLP0

Iteration for the algorithm was set at 1000. The perplexity value was 50 while theta was designated at 0.75. All analysis done was done using Office Excel 2016, FlowJo and Cytobank Premium suite (tSNE analyses). A total of 273 cells per condition was used for the analyses.

Single Cell RNA sequencing

Single-cell RNA sequencing was done on 10x Genomics platform using Chromium controller and Chromium Next GEM Single Cell 3' GEM, Library & Gel Bead Kit v3.1 (10x Genomics). Cell hashing (Totalseq, Biolegend) was performed prior to sorting to ensure we control for batch-to-batch variation between sample conditions. cDNA library synthesis was done on barcoded and pooled samples, including separate libraries for gene expression cDNA and hash tagged oligonucleotide (HTO) libraries. Sequencing of cDNA library was carried out with a commercial sequencing service (Novagene) and analysis of raw data was done using the following software: Cell Ranger, Loupe browser, SeqGeq, MetaCore, Plotly (python), and Excel.

Arteriovenous fistula disease mouse model creation and optimization

To assess a second maladaptive vein disease, we proceeded to create a surgical arteriovenous fistula (AVF) in fat fed Ldlr-/- mice to model AVF failure and disease. We based our surgical technique from previously reported mouse AVF models [26, 46] with some modifications. The Institutional Animal Care and Use Committee at Beth Israel Deaconess Medical Center reviewed and approved all animal experimental protocols (protocol # 021-2017). Male LDL receptor–deficient (Ldlr-/-) mice in a C57BL/6 background were purchased from Jackson Laboratory (Bar Harbor, ME) and maintained with normal chow diet and water ad libitum. Twelve-week-old Ldlr-/- mice were converted to high fat diet containing 1.25% cholesterol (D12108CO; Research Diets, NJ). After a two-week high-fat diet feeding, arteriovenous fistula (AVF) was performed by an end-to-side anastomosis between the left carotid artery and the ipsilateral external jugular vein (EJV) using ten to eleven interrupted stitches of Ethilon 11-0 suture (Owens and Minor, Mechanicsville, VA) per anastomosis. The cut portions of the source EJV were ligated

using Ethilon 8-0 sutures. This procedure generates the arterialized EJV (a-EJV). After the AVF procedure prior to closing the surgical site, blood flow and pulsation of the a-EJV was visually assessed to confirm successful fistula formation. The surgical site is then closed using 6-0 silk sutures.

Gain-of-function study of PPARα in the murine AVF disease model

To test if PPARα activation could produce a similar benefit to AVF disease model as it did for the vein graft disease model, we conducted a similarly randomized gain-of-function study on the mouse AVF model. We used 22 fat-fed Ldlr-/- male mice, aged 12 weeks old, randomized into two groups: (1) a control group fed high fat diet and (2) a treatment group fed with high fat diet admixed with pemafibrate (Kowa, Inc.). The diet was designed to ensure that the AVF mice in the treatment group would receive 0.2 mg/kg body weight/ day dose of pemafibrate daily (D14101401i, Research Diets, New Brunswick, NJ). Mice were pre-fed with either control or treatment food one week prior to AVF surgery and continued on this diet for seven weeks post-surgery. The AVF was non-invasively monitored weekly using high resolution vascular ultrasound imaging with the Vevo 2100 (Fujifilm Visualsonics) up to seven weeks post-AVF. The patency of the AVF among the groups was assessed by color doppler ultrasound overlaid in B-mode during the third week post AVF surgery. If there was color doppler signal in the a-EJV, we called it as "open" or "patent" due to high velocity turbulent flow of blood as detected by the color doppler. If there was no color doppler signal, we called this as "non-patent" or "closed". However, we are aware that absence of color doppler signal may also mean that the stenosis is significant enough to slow down the velocity of the blood flow to an extent below the threshold of detection by the color doppler, even at the lowest pulse repetition frequency (PRF) threshold of 1.0. VevoLab software version 1.6.0 build 6078 (Fujifilm Visualsonics) was used in the assessment. At seven weeks post -AVF surgery, the animals were sacrificed and the AVF venous limb, the a-EJV, was

harvested and processed for histology. Majority of the neointima formation is in the proximal third of the a-EJV nearest to the anastomosis site. By taking this proximal third segment (~1.2 mm for every AVF study mice), we sectioned and took this proximal third segment's mid-length cross section and stained for Hematoxylin and Eosin (H&E). Histologic examination was done to assess for patency ("open") of the a-EJV lumen described as having minimum stenosis of 30% patency. Otherwise, we designate that specimen as "occluded" or "closed". Statistical analysis was done using GraphPad Prism software calculating for the Fisher's exact as a measure for statistical significance of categorical variable (observations).

Seahorse metabolic assays

We assessed energy metabolism in LPS stimulated macrophages and the influence of perturbation via PPARα activation. The rate of glycolysis of macrophages is inferred by measuring the extracellular acidification rate (ECAR) and the rate of oxidative respiration is inferred by measuring the oxygen consumption rate (OCR). The ECAR and the OCR are measured using the Seahorse Bioanalyzer XF24. We performed two assay setups. First, we used BMDMs to assess to demonstrate the peak time of LPS' influence on cellular metabolism (n=4 donors, from wild type C57BL/6, 12-week-old, male mice). Second, we used human PBMC derive macrophages from buffy coats (n=3 donors) to assess the effect of PPARα in LPS stimulated macrophages with or without PPARα silencing to demonstrate PPARα specific mitigation of the hyper glycolysis happening during pro-inflammatory activation of macrophages. BMDMs were plated onto 24 well XF24 assay plates (Agilent, Santa Clara, CA). Using the Seahorse XF Cell Mito Stress Test Kit (Agilent, Santa Clara, CA) we tested for the ECAR profile of cultured BMDMs with or without LPS stimulation happening during the assay run to identify the time when LPS stimulation reaches maximum (and "plateauing") effect on glycolytic rate (ECAR). Assay procedure was performed according to manufacturer's kit protocol. PBMC derived macrophages

were divided into four sample conditions: (1) LPS stimulated, 5 ppm DMSO co-treatment, control siRNA silencing, (2) LPS stimulated, 100 nM pemafibrate treatment, control siRNA silencing, (3) LPS stimulated, 5 ppm DMSO treatment, PPARα siRNA silencing and (4) LPS stimulated, 100 nM pemafibrate treatment, PPARα siRNA silencing. All siRNA silencing was done 48 hours prior to Seahorse assay. All DMSO and pemafibrate treatment were started 12 hours prior to the assay and continued throughout the assay. 100 nM pemafibrate is made by dissolving pemafibrate powder in DMSO to make a concentration of 4 mM. this was then serially diluted with appropriate cell culture media down to 100 nM concentration accounting for 5 ppm DMSO vehicle included in the final culture media solution.

Metabolomic survey of human primary macrophages

We outsourced metabolomic profiling of human primary macrophages to Metabolon, Inc. We availed of three analysis survey packages: (1) macrophage whole cell metabolomic survey using the **HD4 platform**, (2) whole cell lipidomic survey using the **CLP platform** and the (3) isolated macrophage mitochondria metabolomic survey using the **HD4 platform**. Human PBMC macrophages were cultured from LeukoPak buffy coat preparations (StemCell Technologies, Cambridge MA). For each of the three analyses surveys listed we set up five sample conditions: (1) baseline macrophage status, no LPS stimulation, 12 hours DMSO pretreatment (5 ppm), (2) 12 hours DMSO pretreatment (5 ppm), 1 hour 10 ng/mL LPS treatment + DMSO (5 ppm), (3) 12 hours 100 nM pemafibrate pretreatment, 1 hour 10 ng/mL LPS treatment + 100 nM pemafibrate, (4) 12 hours DMSO pretreatment (5 ppm), 4 hours 10 ng/mL LPS treatment + DMSO (5 ppm), and (5) 12 hours 100 nM pemafibrate pretreatment, 4 hours 10 ng/mL LPS treatment + 100 nM pemafibrate. For each sample condition we used 5-6 biological replicate donors. Each sample-condition specimen submitted contained on average 75 milligrams of frozen cell pellet (or 50 mg mitochondrial pellet) and were submitted to Metabolon as per Metabolon's specimen submission

instructions. Whole cell specimens were harvested by washing the culture plates with ice cold PBS followed by scraping the cells using a cell scraper. Cells were immediately frozen in liquid nitrogen after discarding excess PBS post-centrifugation. Each mitochondria specimen was prepared by harvesting and pooling macrophage cells in culture from ten 10 cm^2 culture dishes, then isolating mitochondria using an isolation procedure that has been previously reported [46,47]. In summary, this 72-sample study examined global metabolic profiles in human macrophages and mitochondrial isolates treated with either LPS alone, or LPS with an unknown drug. Samples were collected at baseline, one hour, and four hours. Global metabolic profiles were determined from the experimental groups outlined in the table below.

Group	Description	Cells – Global	Cells – CLP	Mitochondria – Global
Baseline (M0)	Baseline untreated cells	N=4	N=4	N=4
LPS 1HR	Cells stimulated with LPS for 1 hour	N=5	N=5	N=5
LPS 4HR	Cells stimulated with LPS for 4 hours	N=5	N=5	N=5
LPS+Drug 1HR	Cells stimulated with LPS and pemafibrate for 1 hour	N=5	N=5	N=5
LPS+Drug 4HR	Cells stimulated with LPS and pemafibrate for 4 hours	N=5	N=5	N=5

The HD4 platform measures global metabolomic profiles. In Metabolon Discovery HD4™ Platform, samples were inventoried and immediately stored at -80°C. Each sample received was accessioned into the Metabolon LIMS system and was assigned by the LIMS a unique identifier that was associated with the original source identifier only. This identifier was used to track all sample handling, tasks, results, etc. The samples (and all derived aliquots) were tracked by the LIMS system. All portions of any sample were automatically assigned their own unique identifiers by the LIMS when a new task was created; the relationship of these samples was also tracked. All samples were maintained at -80C until processed. Samples are then prepared using the automated MicroLab STAR® system from Hamilton Company. Several recovery standards were

added prior to the first step in the extraction process for quality control, QC purposes. To remove protein, dissociate small molecules bound to protein or trapped in the precipitated protein matrix, and to recover chemically diverse metabolites, proteins were precipitated with methanol under vigorous shaking for two minutes (Glen Mills GenoGrinder 2000) followed by centrifugation. The resulting extract was divided into five fractions: two for analysis by two separate reverse phase (RP)/UPLC-MS/MS methods with positive ion mode electrospray ionization (ESI), one for analysis by RP/UPLC-MS/MS with negative ion mode ESI, one for analysis by HILIC/UPLC-MS/MS with negative ion mode ESI, and one sample was reserved for backup. Samples were placed briefly on a TurboVap® (Zymark, Hopkinton, MA) to remove the organic solvent. The sample extracts are stored overnight under nitrogen before preparation for analysis.

On the other hand, the Complex Lipid Platform (CLP) performs lipidomic survey on the cell samples. Lipids were extracted from samples in methanol:dichloromethane in the presence of internal standards. The extracts were concentrated under nitrogen and reconstituted in 0.25mL of 10 mM ammonium acetate dichloromethane:methanol (50:50). The extracts were transferred to inserts and placed in vials for infusion-MS analysis, performed on a Shimazdu LC with nano PEEK tubing and the Sciex SelexIon-5500 QTRAP. The samples were analyzed via both positive and negative mode electrospray. The 5500 QTRAP scan was performed in MRM mode with the total of more than 1,100 MRMs. Individual lipid species are quantified by taking the peak area ratios of target compounds and their assigned internal standards, then multiplying by the concentration of internal standard added to the sample. Lipid class concentrations were calculated from the sum of all molecular species within a class, and fatty acid compositions were determined by calculating the proportion of each class comprised by individual fatty acids.

Mitochondrial fitness assay by flow cytometry

PBMC derived macrophages are used for this flow cytometric analysis. Macrophages in culture are silenced with either control siRNA or human PPARα siRNA at 40 nM using

SilenceMag. After 48 hours, cells from the siControl and siPPARα conditions were preincubated with either 5 ppm DMSO or 100 nM pemafibrate for 12 hours. Subsequently, cells were treated with 10ng/mL LPS for 3 hours +/- pemafibrate or DMSO to continue previous pretreatment. Cells were then incubated for 30 minutes at 37°C with Tetramethylrhodamine, Methyl Ester, Perchlorate, TMRM (Thermo Fisher, Cambridge, MA) while under growth media by adding TMRM in the media for a final concentration of 50 nM. Growth media was replaced, and cells were detached from the cultured plate by scraping with a cell scraper. Cells were then assayed in using a BD FACS Aria II SORP flow cytometer. High TMRM staining indicated the cells had "healthy" mitochondria with high membrane gradient potential, while low TMRM staining indicated cells possessing "leaky" mitochondria with low membrane gradient potential. In a parallel sample conditions setup, cells were stained with MitoSOX™ Red Mitochondrial Superoxide Indicator (Thermo Fisher, Cambridge, MA) similarly at a staining concentration of 5 μM for 10 minutes in 37°C. Staining solution is similarly washed, and cells were detached for flow cytometric analysis. High MitoSox staining indicates high probability of reactive oxygen species in the mitochondria causing membrane damage leading to "leakiness" and low gradient membrane potential. Flow cytometric histograms were produced using BD FACS Diva software ver.

Time lapse microscopy of macrophage mitochondria

Using cultured RAW 264.7 cells (ATCC, Manassas, VA) stimulated with LPS, cells were stained with TMRM and MitoTracker Green, a membrane potential independent stain for the mitochondria. Disappearance of TMRM staining as cells are exposed to LPS in a 60-minute incubation was recorded in time lapse fluorescence microscopy using a Nikon inverted microscope

SUPPLEMENTAL FIGURES
I. - XX.

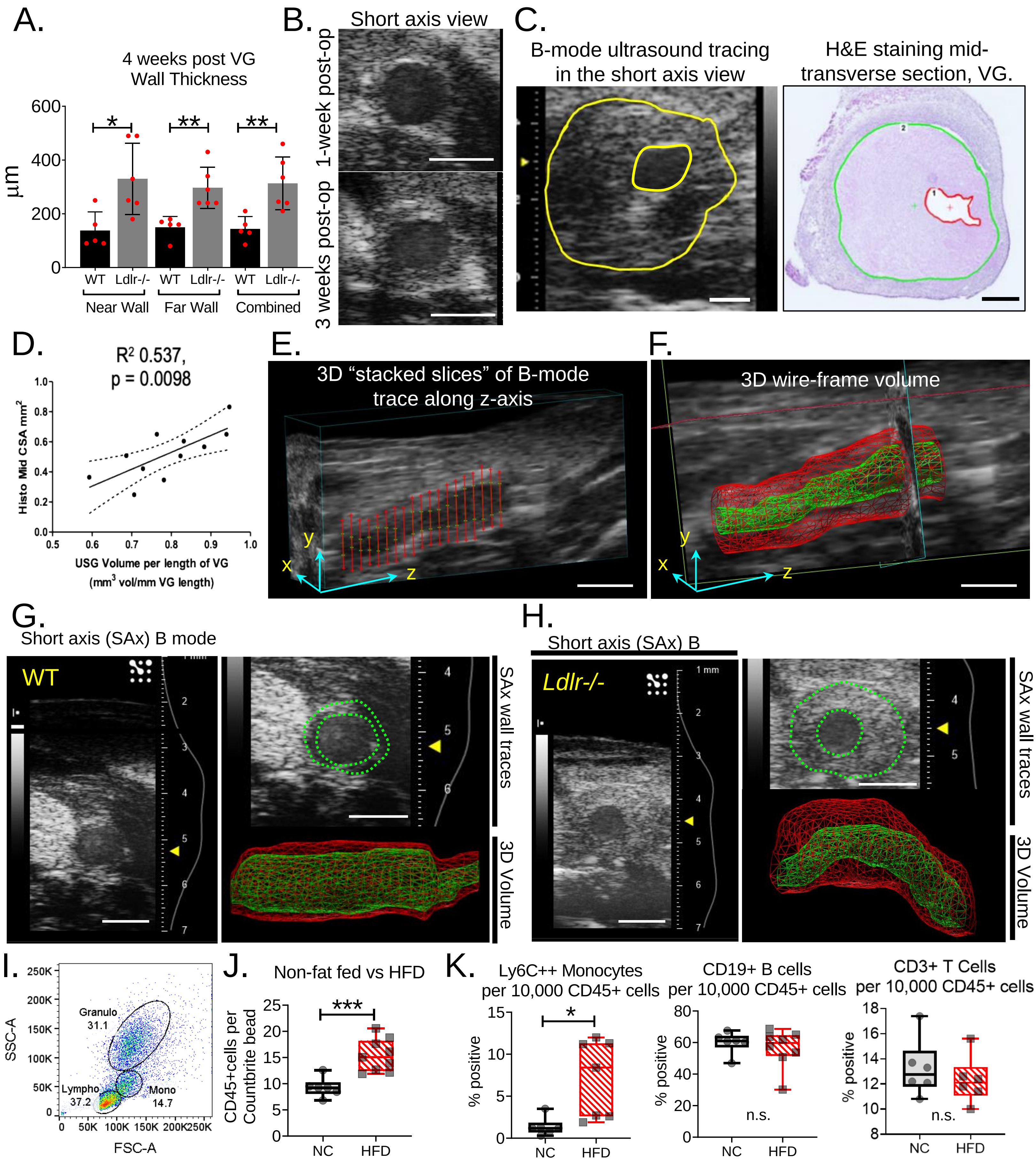

Figure I. Mouse vein graft (VG) disease model. A. Plaque burden of wild type (WT) and *Ldlr-/-* mice, estimated as wall thickness. (*) $p < 0.05$, (**) $p < 0.01$. **B.** Short axis (SAx) view of the *Ldlr-/-* vein grafts, B-mode, 1-week vs. 3-weeks post-surgery. **C.** VG plaque B mode SAx plaque tracing, representative image (scalebar 500 µm), and counterpart H&E histologic morphometry of mid-cross-sectional area (scalebar 500 µm) and their **(D.)** correlation plot, n=11 mice. **E.-F.** 3D ultrasound volumes. Long axis view, LAx (scale bar 1 mm) **G.** Wild type vein graft 4-week time point ultrasound imaging in SAx B mode and 3D volume wireframe rendering. Representative images. Lumen and outer wall tracings in green and red, respectively **H.** *Ldlr-/-* vein graft 4-week time point ultrasound imaging. SAx B-mode and 3D volume wireframe rendering. Representative images. Scale=1 mm. **I.** FACS plot of CD45+ cells from the whole blood (RBCs lysed and residuals gated out) of fat-fed *Ldlr-/-* mice. Event percentages of Granulo=granulocytes, Mono=monocytes, Lympho=lymphocytes shown. **J.** CD45+ cell count between non-fat fed (normal chow, NC) animals vs. fat-fed (high-fat diet, HFD), after four weeks, counts are normalized using CountBright™ absolute counting beads (BD Biosciences). **K.** Differential WBC count normalized per 10,000 CD45+ cells for Ly6C++ monocytes, CD19+ B cells and CD3+ T cells. (*) $p < 0.05$, (***) $p < 0.001$, (n.s.) $p \geq 0.05$.

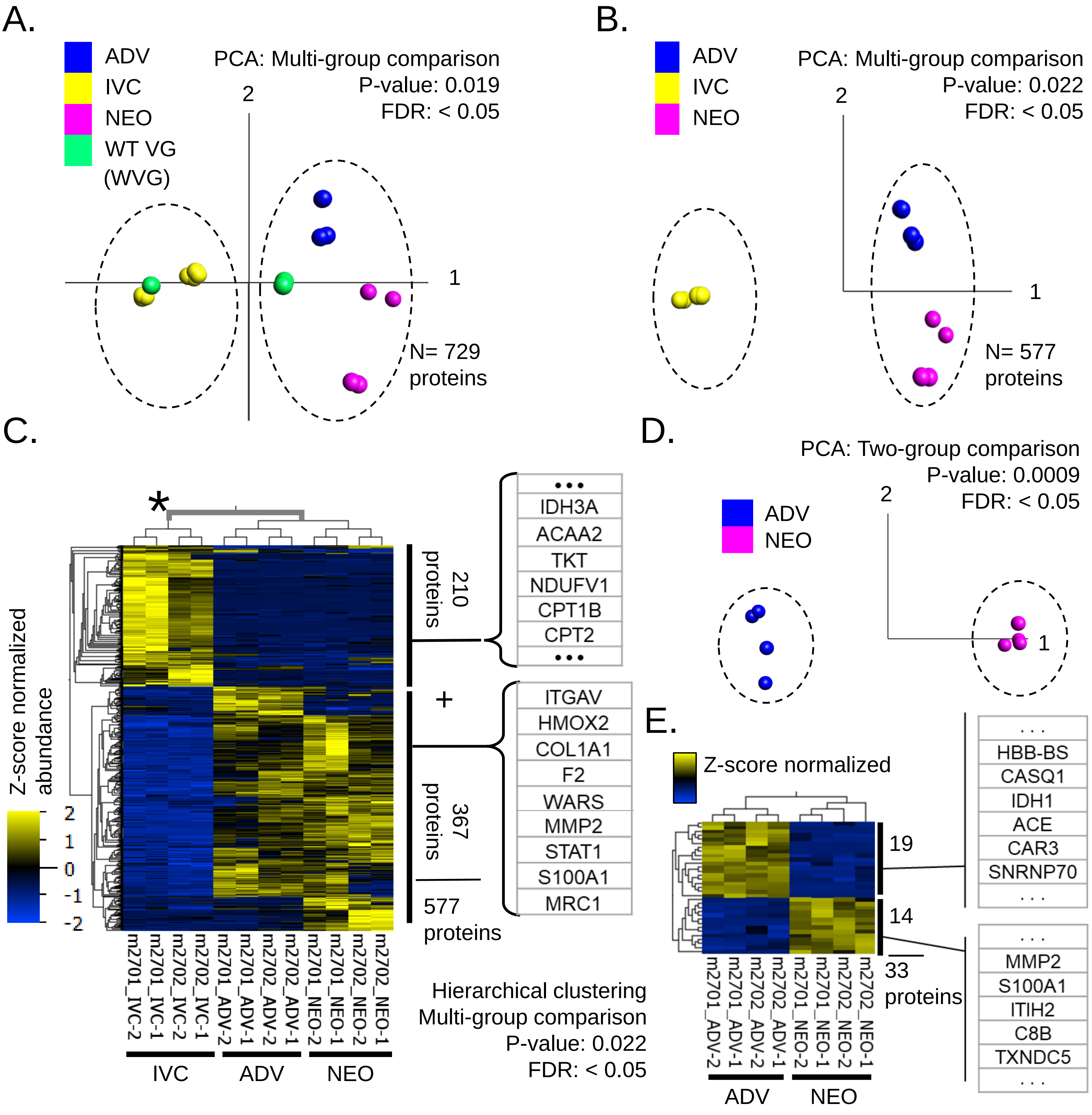

Figure II. Mouse vein graft tissue layer and vein graft time course proteomics. A. Principal component analysis (PCA) of WT vein graft (WVG) and *Ldlr-/-* vein graft tissue layers (NEO and ADV) and corresponding IVCs (WT and *Ldlr-/-*) proteome. Multi-group comparisons filtered for p-value < 0.05 and false discovery rate, FDR < 0.05 yields 729 shared proteins among the tissue samples **B.** Similar analysis with PCA of *Ldlr-/-* vein graft tissue layers and their counterpart IVC only. **C.** Proteome profile of *Ldlr-/-* NEO, ADV and IVC in hierarchical clustering confirms what PCA clustering showed: IVC samples cluster separately from NEO and ADV as seen on the first level of hierarchical clustering (*) of their proteomic profile. NEO and ADV may be more similar (expressing inflammation and thrombosis proteins) versus IVC in proteomic profile (cell respiration proteins) based on the multi-group comparison expressing. **D.** PCA of NEO and ADV without the effect of IVC samples showing the distribution of NEO and ADV samples after two-group comparison with FDR < 0.05. **E.** Proteomic profile of the same two-group comparison of NEO and ADV as an expression heatmap. All expression heatmaps are z-score normalized. All multi-group and two-group comparisons are made with a threshold of p-value < 0.05, FDR < 0.05. ADV= adventitial layer of the vein graft sample. IVC= inferior vena cava endogenous control. NEO= neointimal layer of the vein graft sample.

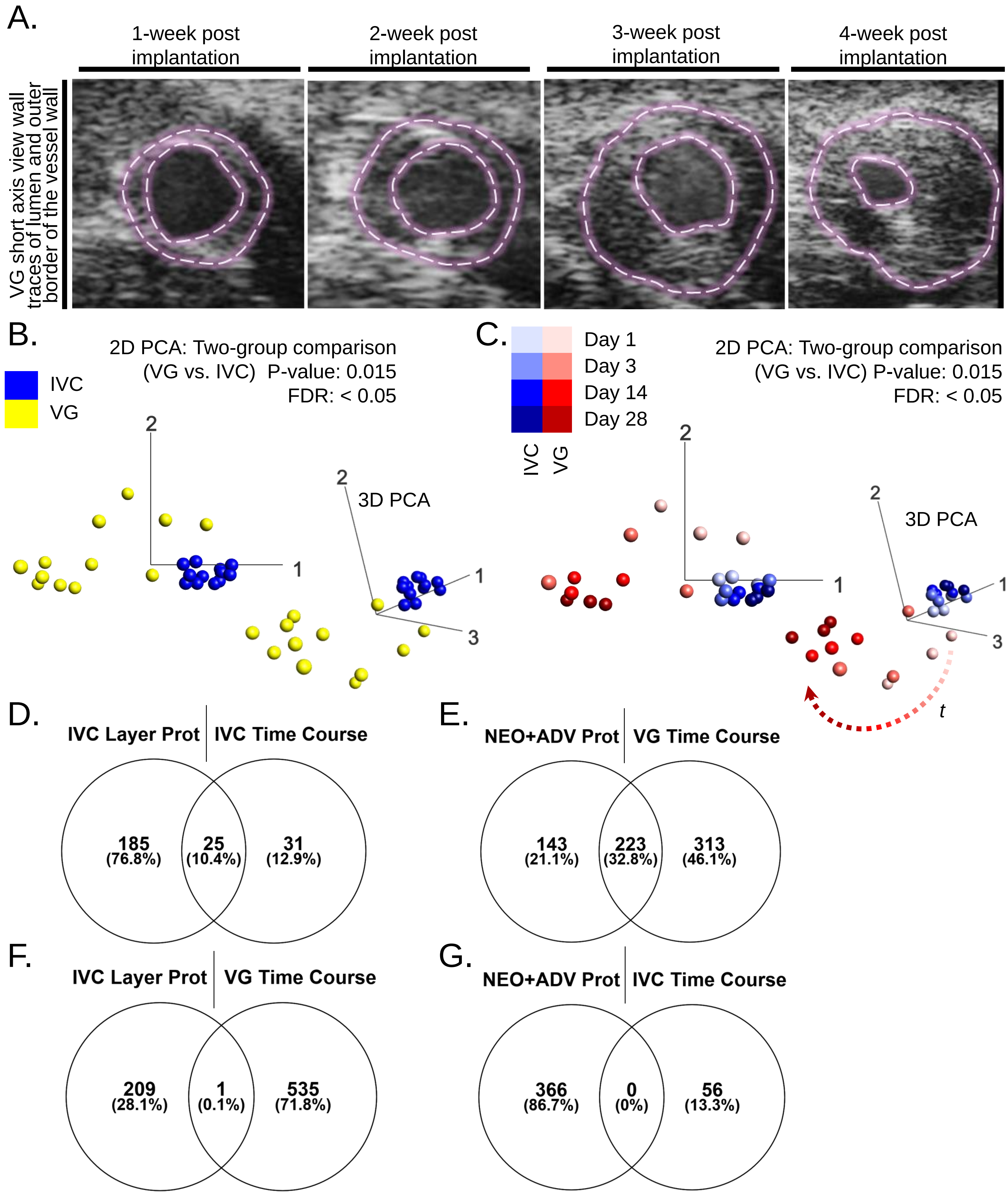

Figure III. Mouse vein graft time course proteomics. A. Short axis B-mode images of *Ldlr-/-* vein graft time course: 1 week, 2 weeks, 3 weeks, and 4 weeks after vein graft implantation. Lumen and outer wall tracings in white broken lines. **B.** Principal component analysis, PCA of all time course samples colored by tissue type: VG or IVC. **C.** PCA of all VG samples in the time course proteomics depicted per timepoint. Venn diagrams showing shared proteins between IVC Layer/static and IVC time course predominant proteins **(D.)**, NEO+ADV from static and whole vein graft (VG) time course predominant proteins **(E.)**, IVC Layer/static and VG time course predominant proteins **(F.)**, and NEO+ADV static and IVC time course predominant proteins **(G.)**

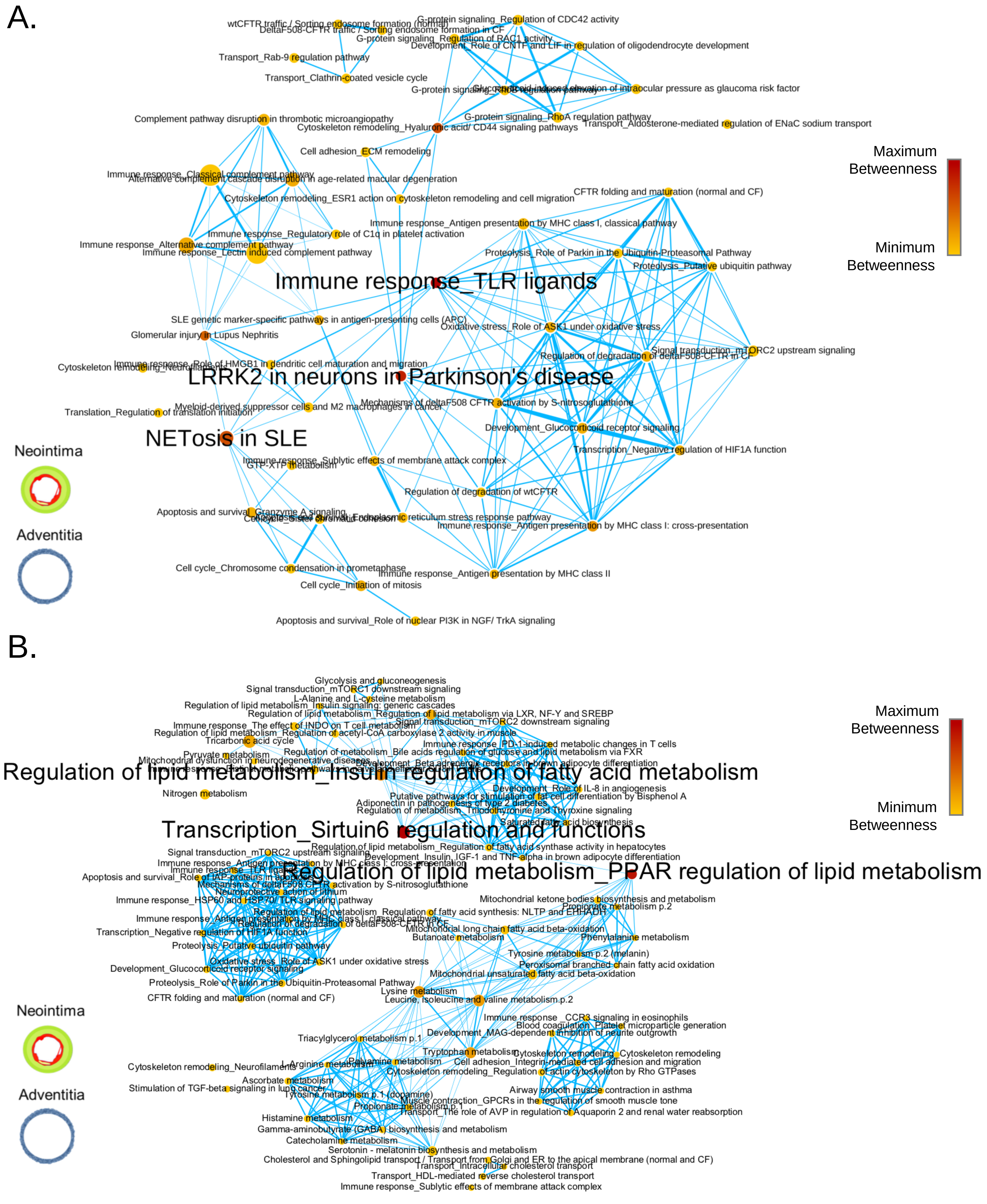

Figure IV. Network of enriched pathways. Static proteomics A. *Ldlr*-/- VG neointimal and adventitial predominant proteins pathways enrichment network. **B.** IVC and wild type VG predominant proteins pathways enrichment network. Node sizes are proportional to the number of proteins from the dataset that are present in the pathway node. Node color depicts the intensity of betweenness centrality for a specific pathway node.

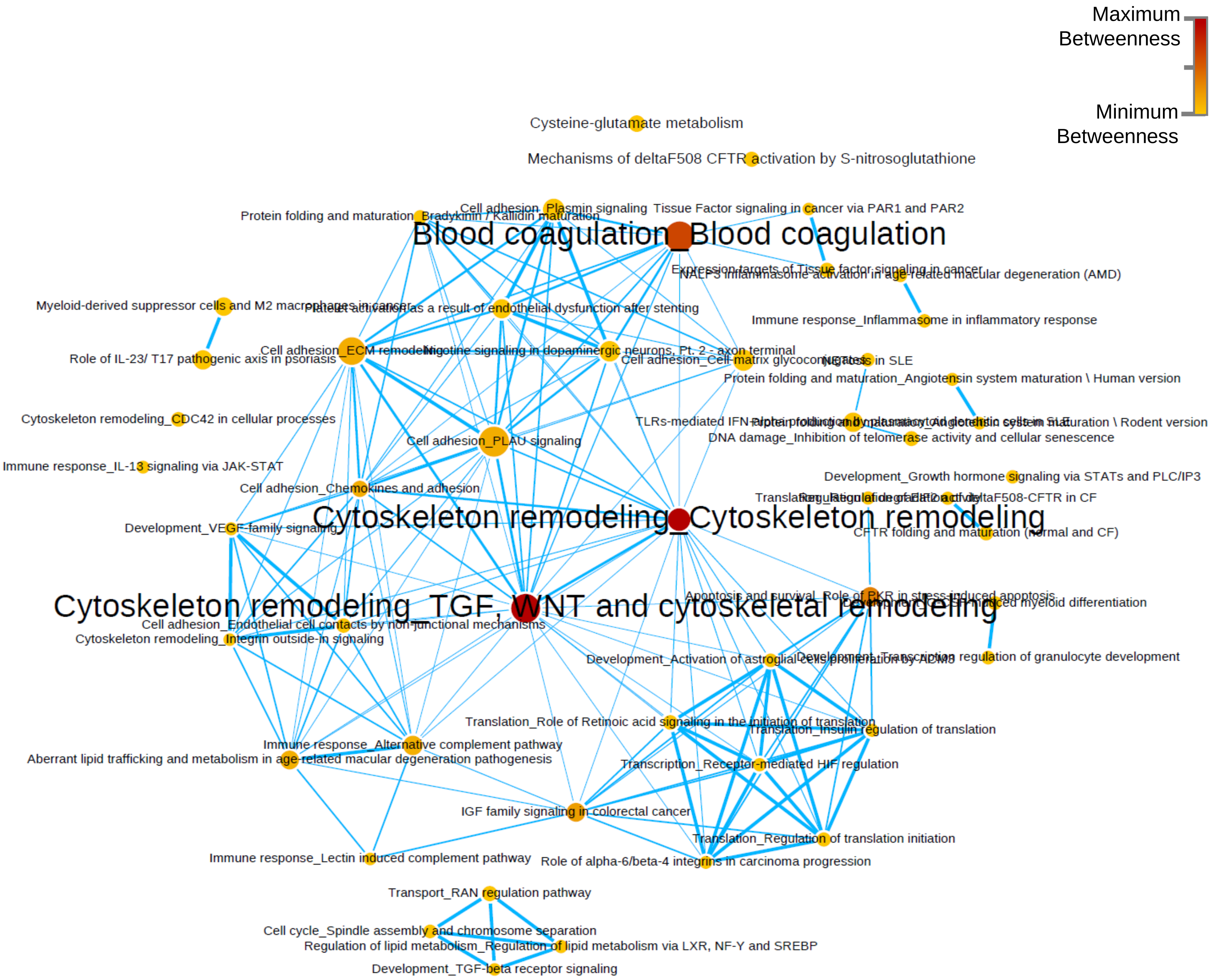

Figure V. Network of enriched pathways. Time course proteomics. *Ldlr*-/- VG early timepoints predominant proteins pathways enrichment network. Node sizes are proportional to the number of proteins from the dataset present in the pathway node. Node color depicts the intensity of betweenness centrality for a specific pathway node.

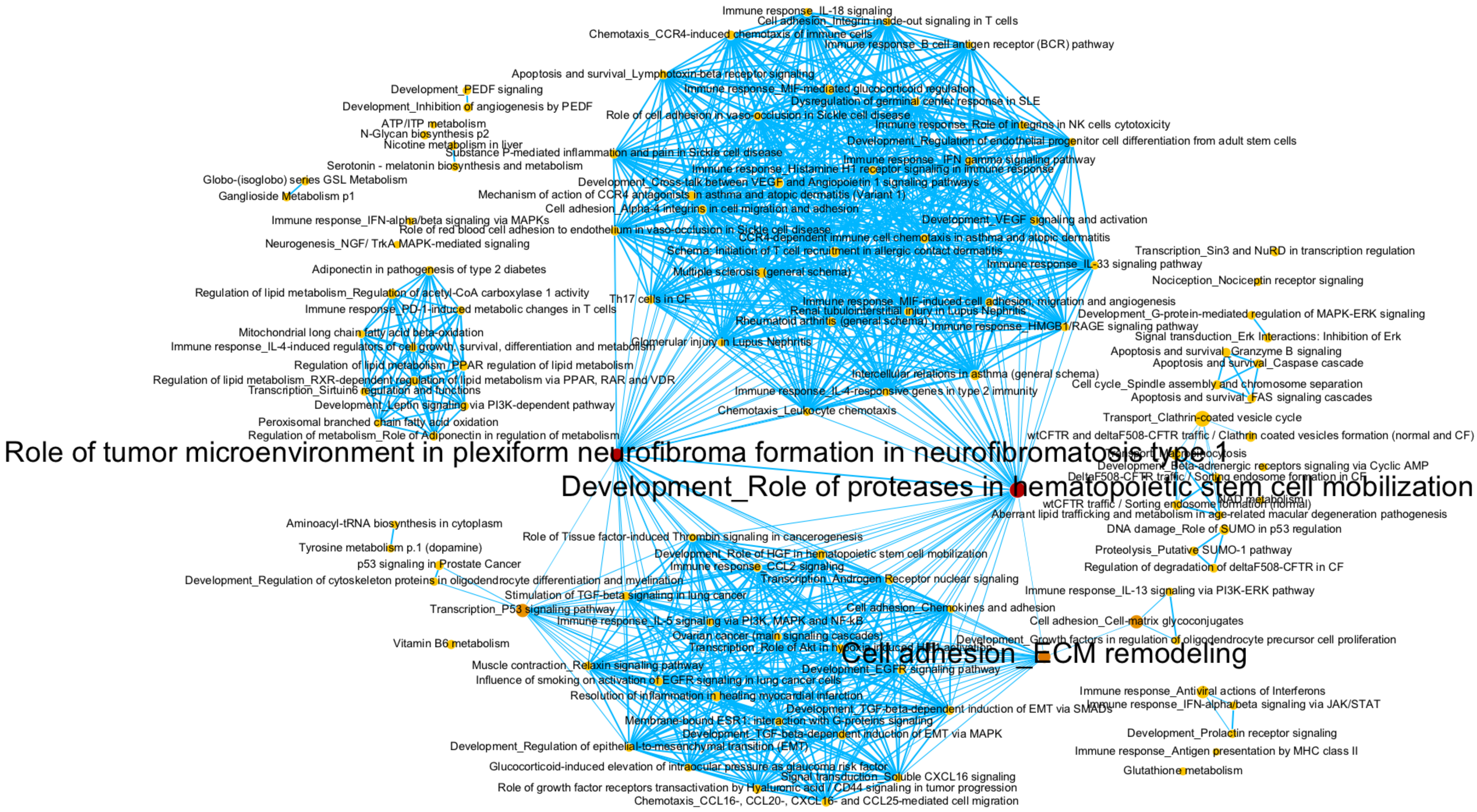

Figure VI. Network of enriched pathways. Time course proteomics *Ldlr-/-* VG late timepoints predominant proteins pathways enrichment network. Node sizes are proportional to the number of proteins from the dataset present in the pathway node. Node color depicts the intensity of betweenness centrality for a specific pathway node.

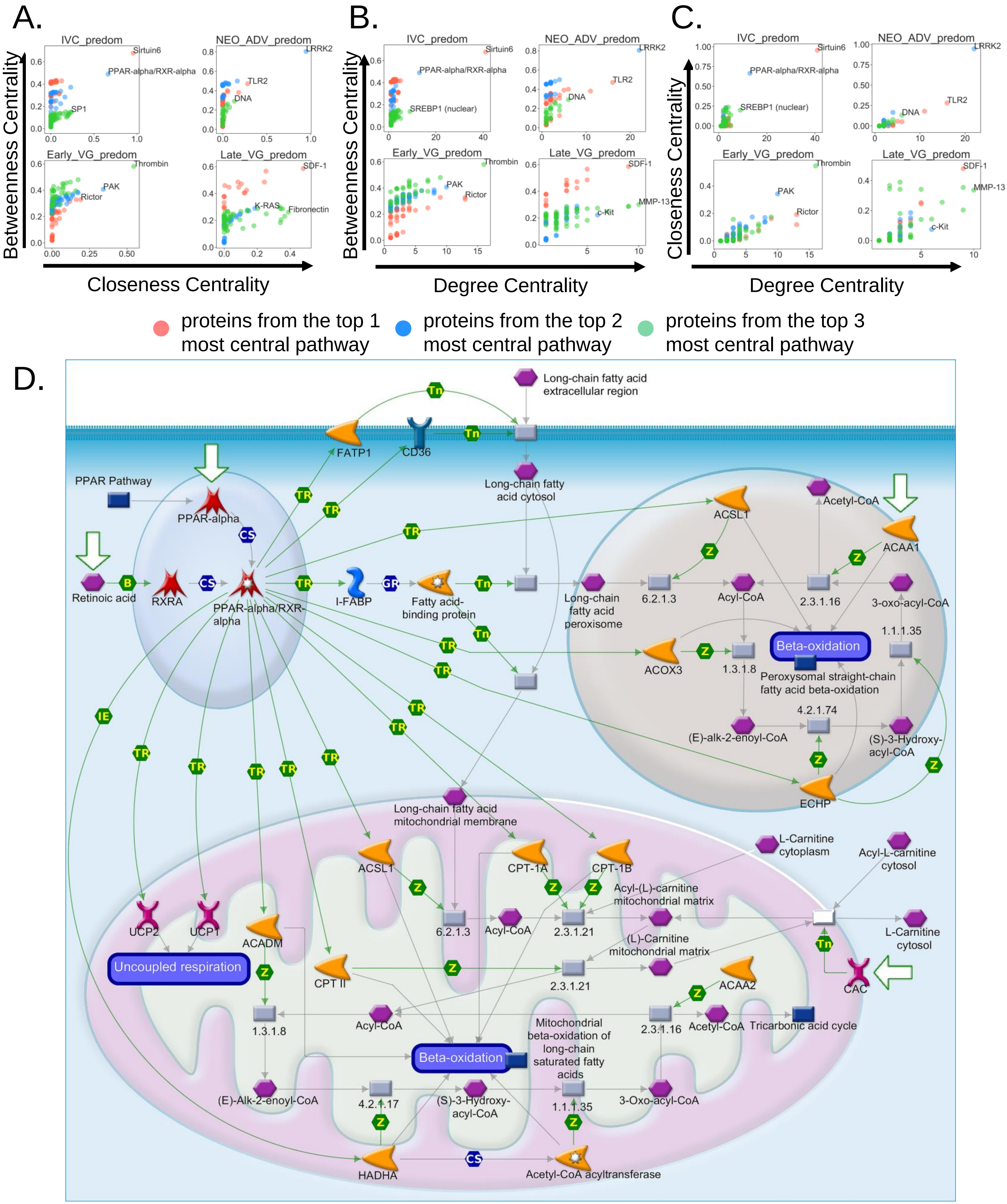

Figure VII. Measures of hub centrality for proteins within the top 3 most central pathway nodes of each of the proteomic networks. A. Scatterplot of betweenness centrality by closeness centrality, (**B.**) betweenness centrality by degree centrality, and (**C.**) closeness centrality by degree centrality. Red dots represent proteins found in the proteomic dataset from the top 1 biological pathway for the indicated sample condition group (e.g., NEO/ADV predominant proteins group). Blue dots are proteins from the top 2 biological pathways. Green dots are proteins from the top 3 pathway. **D.** PPARα lipid regulation pathway (MetaCore) in detail.

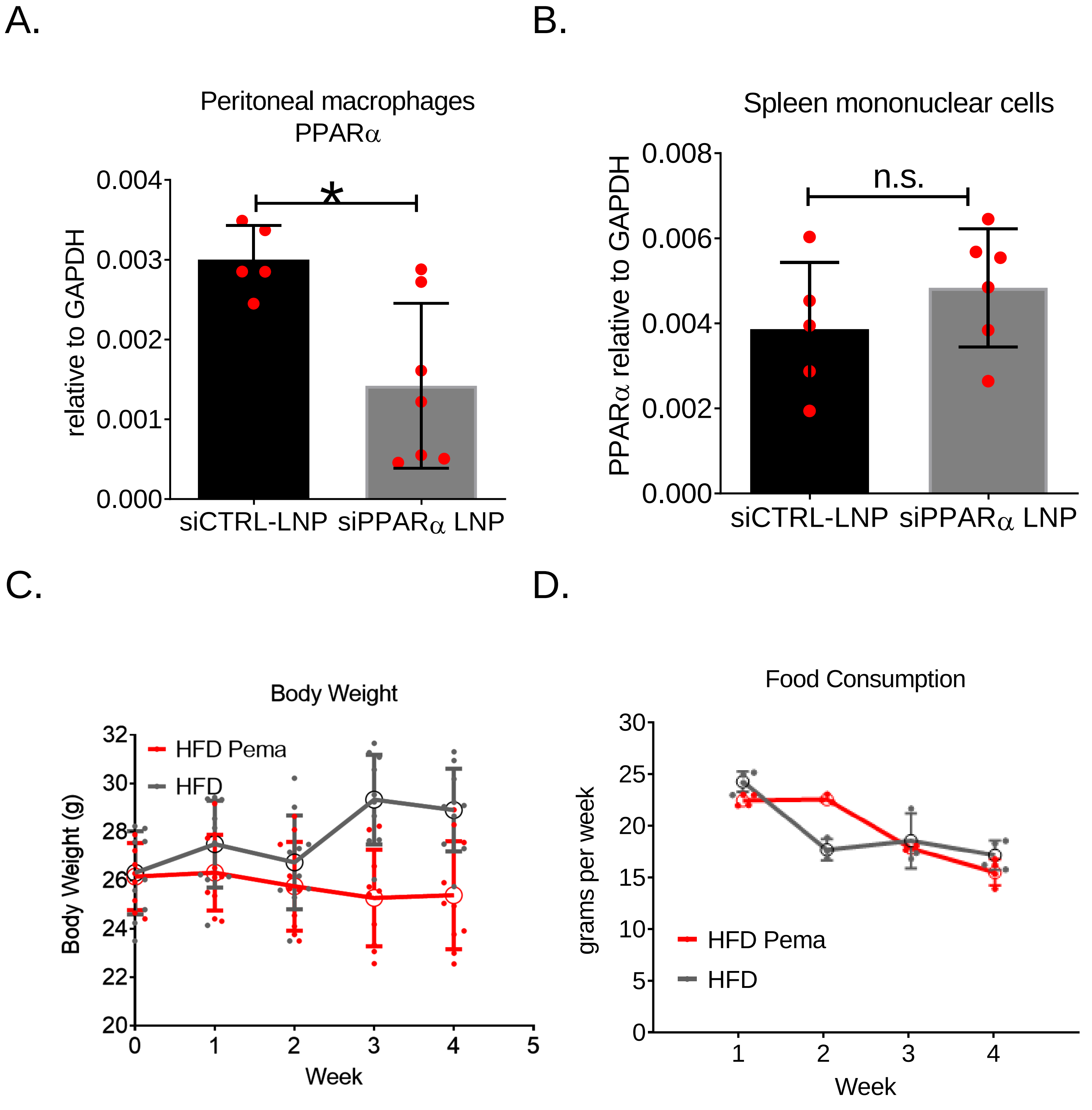

Figure VIII. A. Optimization of silencing of PPAR αusing siRNA lipid nanoparticle (C12-200, LNP) conjugates. siControl (n=5) and siPPAR α(n=6) groups of *Ldlr-/- mice* were used. siRNA LNP were injected in the tail vein at a dose of 0.5 mg/kg/dose twice a week for three weeks. Animals were challenged with an intraperitoneal injection of 2% thioglycolate (w/v) for 48 hours (pain medications were appropriately given). Afterward, peritoneal lavage was done to harvest the peritoneal macrophages. **B.** Spleens were also harvested, and mononuclear cells were isolated by homogenizing the spleen and gradient centrifugation (Histopaque 1083). qPCR of peritoneal macrophages and spleen mononuclear preps were done to assess silencing. (*) $p < 0.05$, (n.s.) $p \geq 0.05$. **C.** Bodyweight monitoring between Pemafibrate and Control group, n=11 mice per group comparison **D.** Food consumption monitoring between Pemafibrate and Control group, n=11 mice per group comparison (per cage average).

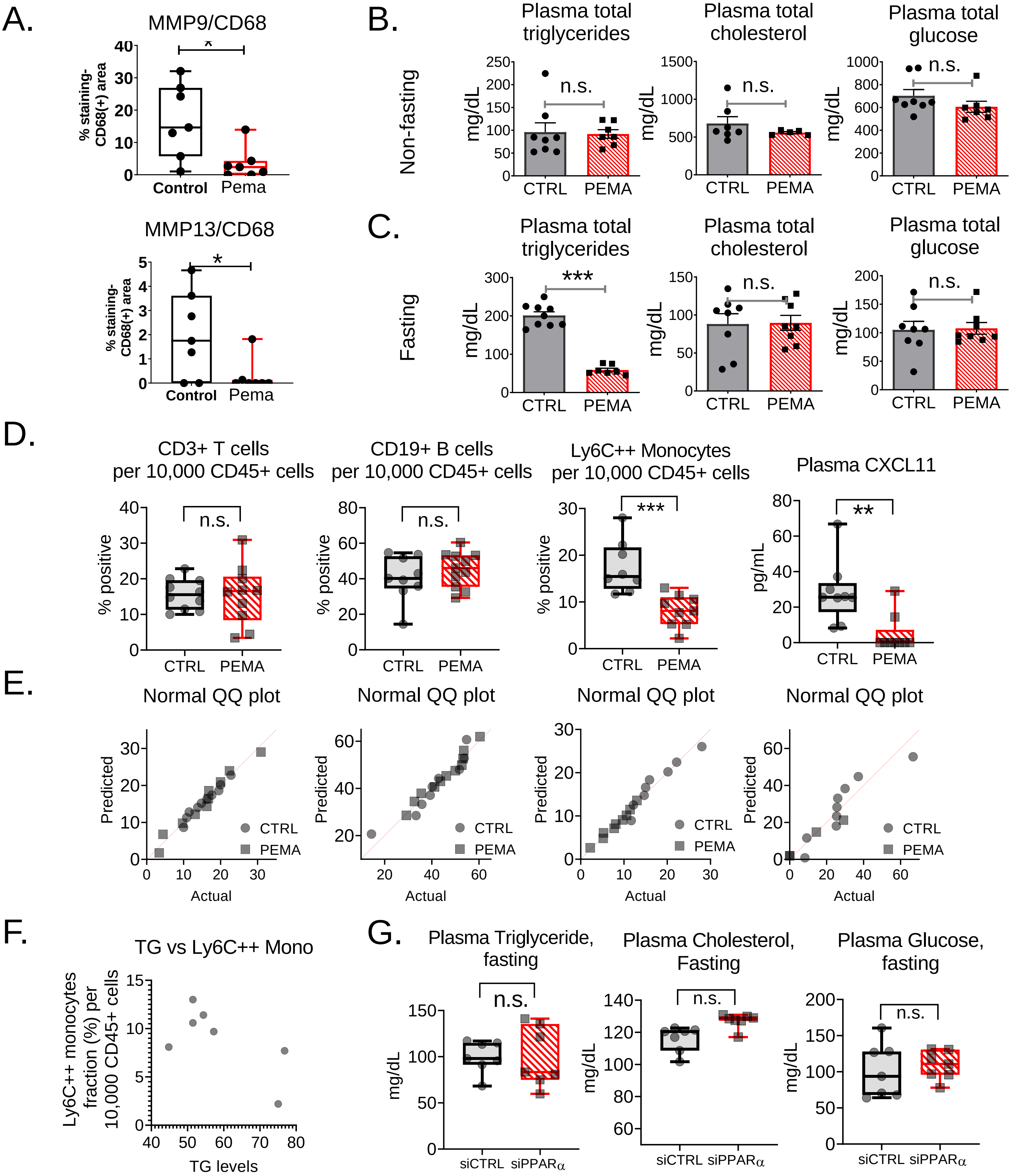

Figure IX. Immune cells and lipid profile assessment *in vivo*. A. Protease expression of MMP9 and MMP13 per CD68 staining is also significantly less in pemafibrate treated vein grafts. **B.** Non-fasting and (**C.**) fasting blood levels of triglycerides, cholesterol, and glucose between two groups: control vs. pemafibrate treated group (n=7-9 mice per group). Fasting was done for 17 hours. **D.** Differential WBC count between control and pemafibrate group, ELISA quantitation of blood circulating CXCL11, a chemokine secreted by monocytes. **E.** Corresponding normality plots. CTRL= control group, PEMA= pemafibrate group. **F.** Spearman correlation plot showed no statistical correlation between TG levels and circulating Ly6C++ monocyte fraction among the pemafibrate treated group. **G.** Fasting plasma concentrations of triglycerides, cholesterol, and glucose between control siRNA group (siCTRL, n=7) and PPAR αsilenced group (siPPARα, n=7). (*) $p < 0.05$, (**) $P < 0.01$, (***) $p < 0.001$, (n.s.) $p \geq 0.05$.

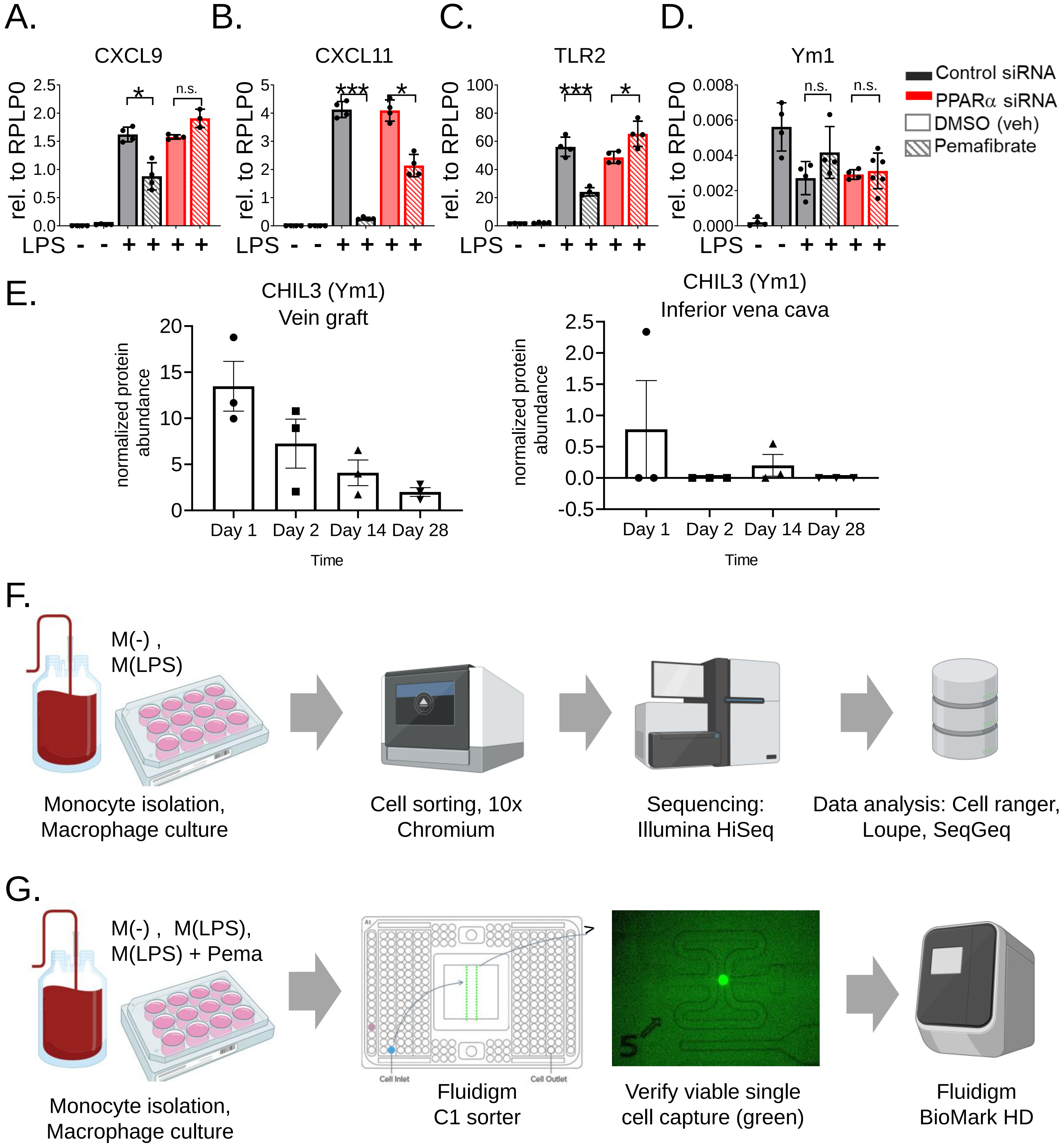

Figure X. ***In vitro*** **validation: mRNA expression in primary macrophages. A.** Bulk qPCR of pro-inflammatory and anti-inflammatory markers in mouse bone marrow derived macrophages (BMDM), with loss-of-function (PPARα siRNA) and gain-of-function (PPARα agonism by pemafibrate – 100nM). CXCL9 , CXCL11(**B.**), TLR2 (**C.**)and (**D.**) anti-inflammatory marker Ym1 in mouse BMDM primary macrophages, with loss-of-function (PPARα siRNA) and gain-of-function (PPARα agonism by pemafibrate – 100nM). **E.** Relative abundances (normalized values) of proteins by LC/MS/MS of vein graft tissue in time course proteomics and IVC tissue samples in time course proteomics. **F.** Workflow of single cell RNA sequencing. Macrophages are derived from monocytes isolated from buffy coats and then differentiated into macrophages *in vitro*. **G.** Similar workflow for single cell qPCR using Fluidigm C1 sorter and BioMark HD RT PCR. Chil3, chitinase-like 3 (Ym1). (*) $p < 0.05$, (***) $p < 0.001$, (n.s.) $p \geq 0.05$. Green fluorescent viability dye is Calcein AM.

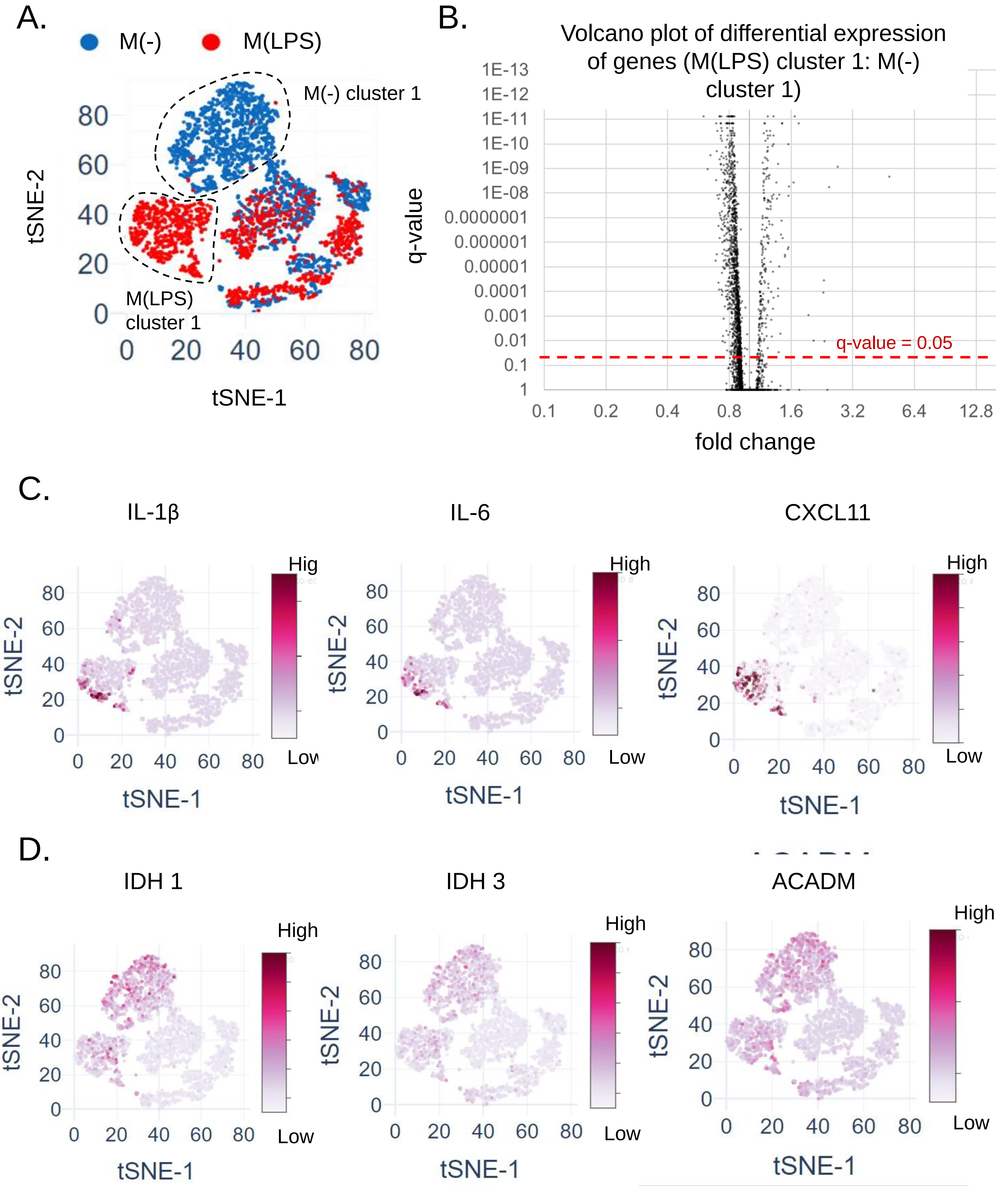

Figure XI. Single-cell RNA sequencing results in tSNE (viSNE) plots. A. tSNE plot M(-) vs. M(LPS), gated clusters of interest: M(-) cluster 1, M(LPS) cluster 1 **B.** Volcano plot of differential gene expression between M(LPS) cluster 1 and M(-) cluster 1 **C.** Single-cell expression of proinflammatory genes IL-1β, IL-6, and CXCL11. **D.** Single-cell gene expression of TCA related genes IDH1, IDH3, and ACADM. TCA= tricarboxylic acid cycle.

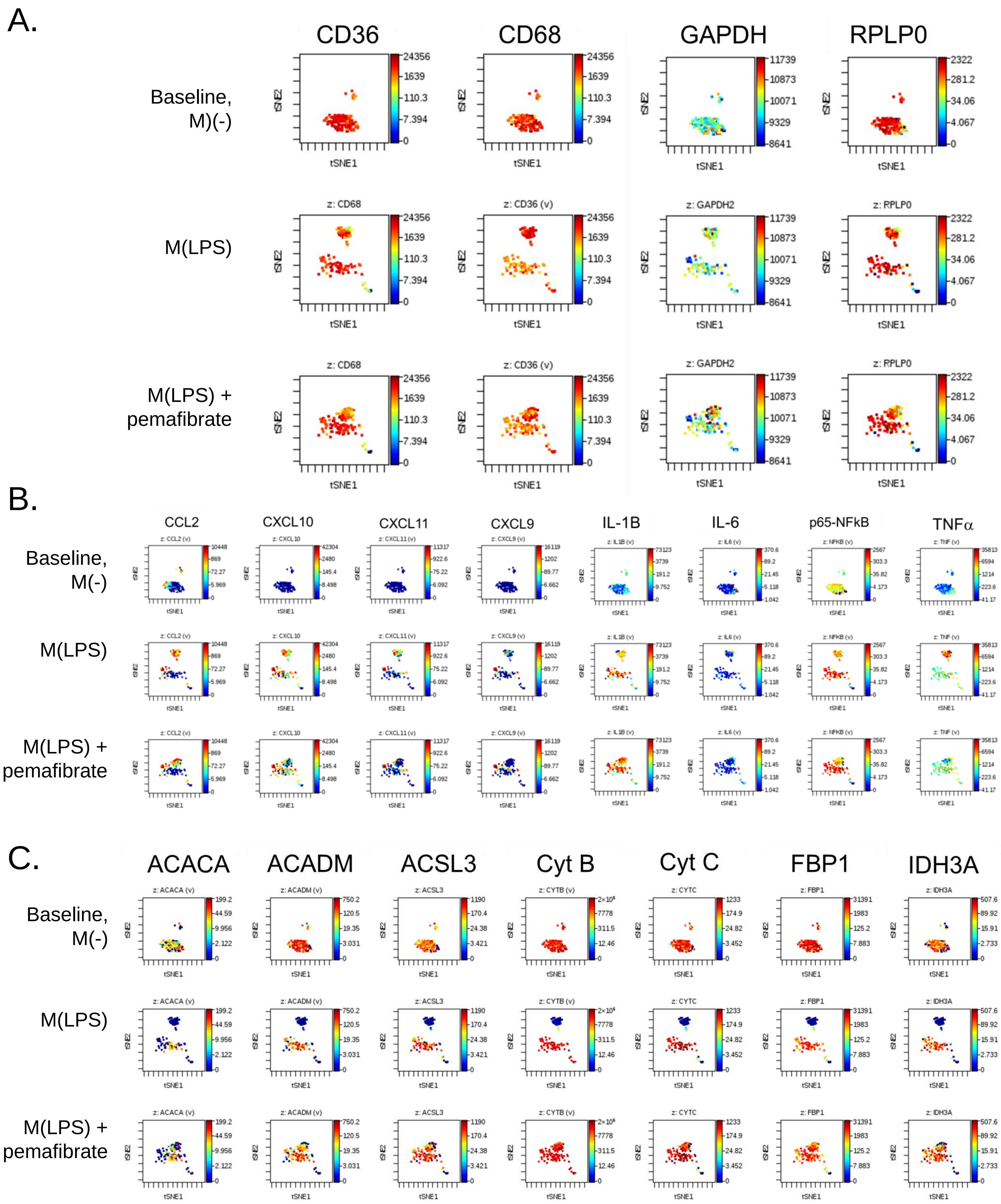

Figure XII. Single-cell qPCR results in tSNE (viSNE) plots. A. Scatterplot heatmap of cells expressing general macrophage markers (**A.**). Scale bar represents relative expression **B.** Pro-inflammatory markers CCL2, CXCL10, CXCL11, CXCL9, IL-1β, IL-6, p65-NFκB, and TNF α**C.** OXPHOS pathways markers (FAO, TCA, ETC). FAO= fatty acid oxidation. TCA= tricarboxylic acid cycle. ETC= electron transport chain. Each dot represents a cell in any of the three conditions being compared: (1) Baseline M(-) or non-stimulated human primary macrophages, monocyte-derived, (2) M(LPS) or LPS stimulated (~10 ng/mL) , and (3) M(LPS) pre- and co-treated with 100 nM pemafibrate (PPAR αagonism)

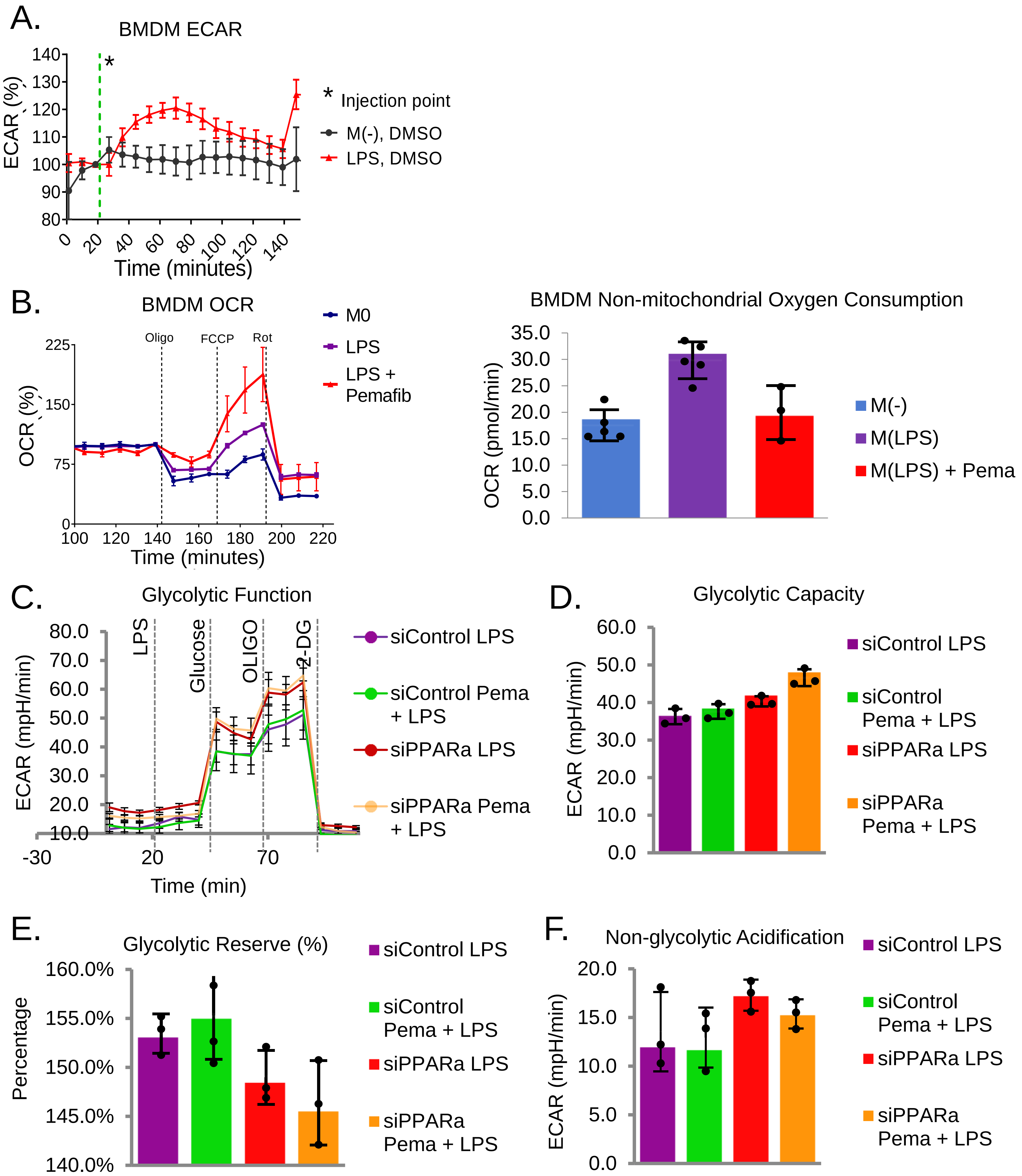

Figure XIII. Seahorse metabolic assays. A. Glycolytic rate as extracellular acidification rate, ECAR, of BMDM cells, M(-) vs. M(LPS) in 140 minutes. LPS (10 ng/mL) or PBS injected at 20 minutes. **B.** Mitochondrial stress test measuring oxidative consumption rate (OCR) as a measure of mitochondrial respiration rate. Non-mitochondrial oxygen consumption, like ROS generation, is also measured, right panel. **C.** Glycolytic function, ECAR mpH/min. **D.** Glycolytic capacity, ECAR mpH/min. **E.** Glycolytic reserve (%). **F.** Non-glycolytic acidification, ECAR mpH/min 2-DG= 2-deoxyglucose, BMDM= bone marrow-derived macrophages. DMSO= dimethyl sulfoxide. ECAR= extracellular acidification rate, FCCP= Carbonyl cyanide-4-(trifluoromethoxy)phenylhydrazone, a mitochondrial uncoupler ionophore. LPS= lipopolysaccharide. Oligo/OLIGO= oligomycin Pema/PEMA= pemafibrate. PPARa= Peroxisome Proliferator-Activated Receptor Alpha. ROS = reactive oxygen species. Rot= rotenone.

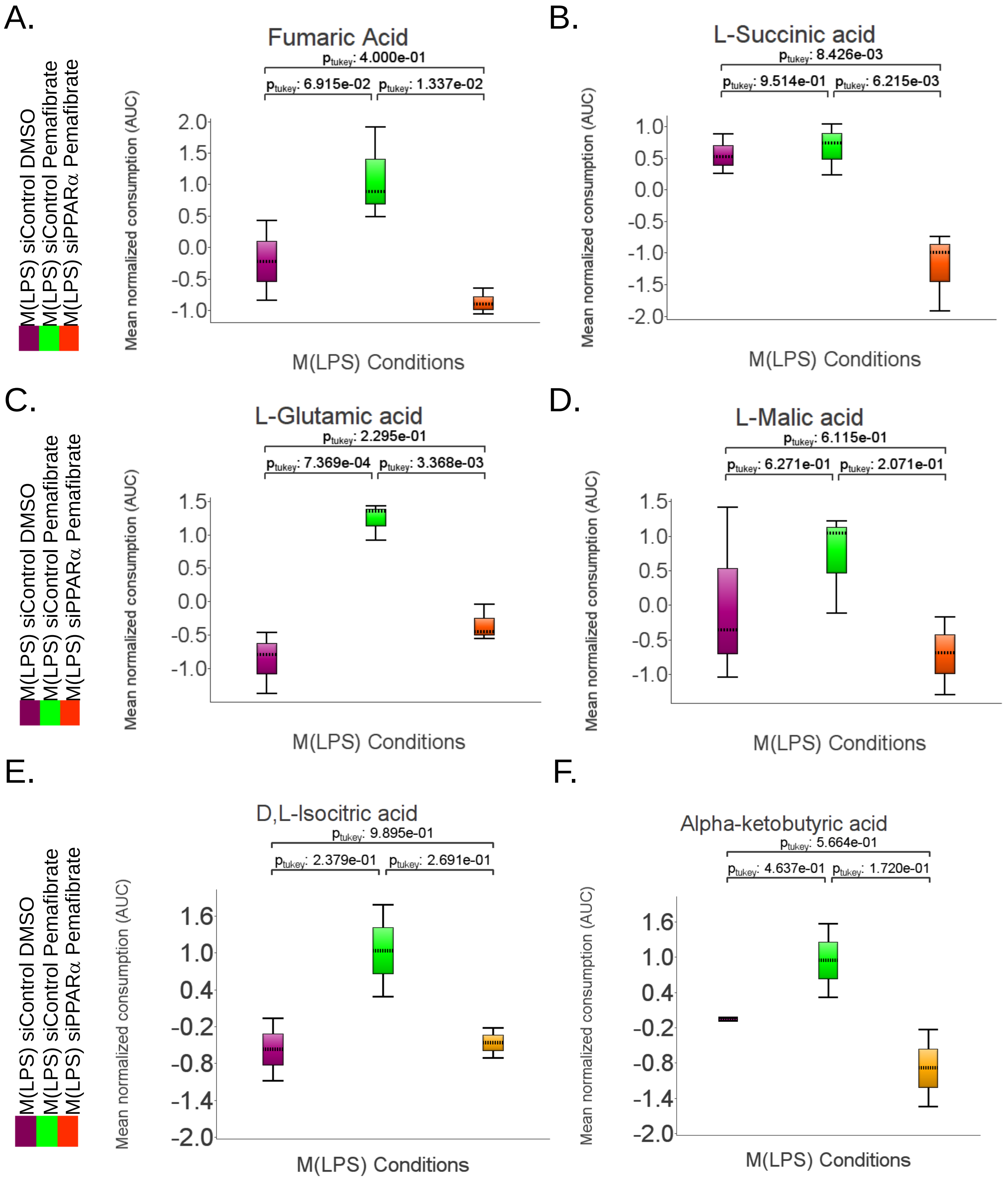

Figure XIV. TCA substrates consumption as measured by Mitoplate S assay. A time-series measurement using the area-under-the-curve (AUC) of TCA substrate utilization in cell count normalized primary human macrophages (n=3 donors). Each assay plate measures only up to three conditions. The conditions tested were M(LPS) (LPS, siControl, DMSO), M(LPS) siControl + Pema, and M(LPS) siPPAR α+ Pema. Fumaric acid (**A.**)**,** succinic acid **(B.**), L-glutamic acid (**C.**), and L-malic acid (**D.**) substrate utilization were measured in human primary macrophages, n = 3 donors. D, L-isocitric acid (**E.**), and α-ketobutyric acid (**F.**) substrate utilization is measured in THP-1 differentiated macrophage-like cells. PPARα= Peroxisome Proliferator-Activated Receptor Alpha; TCA=tricarboxylic acid cycle

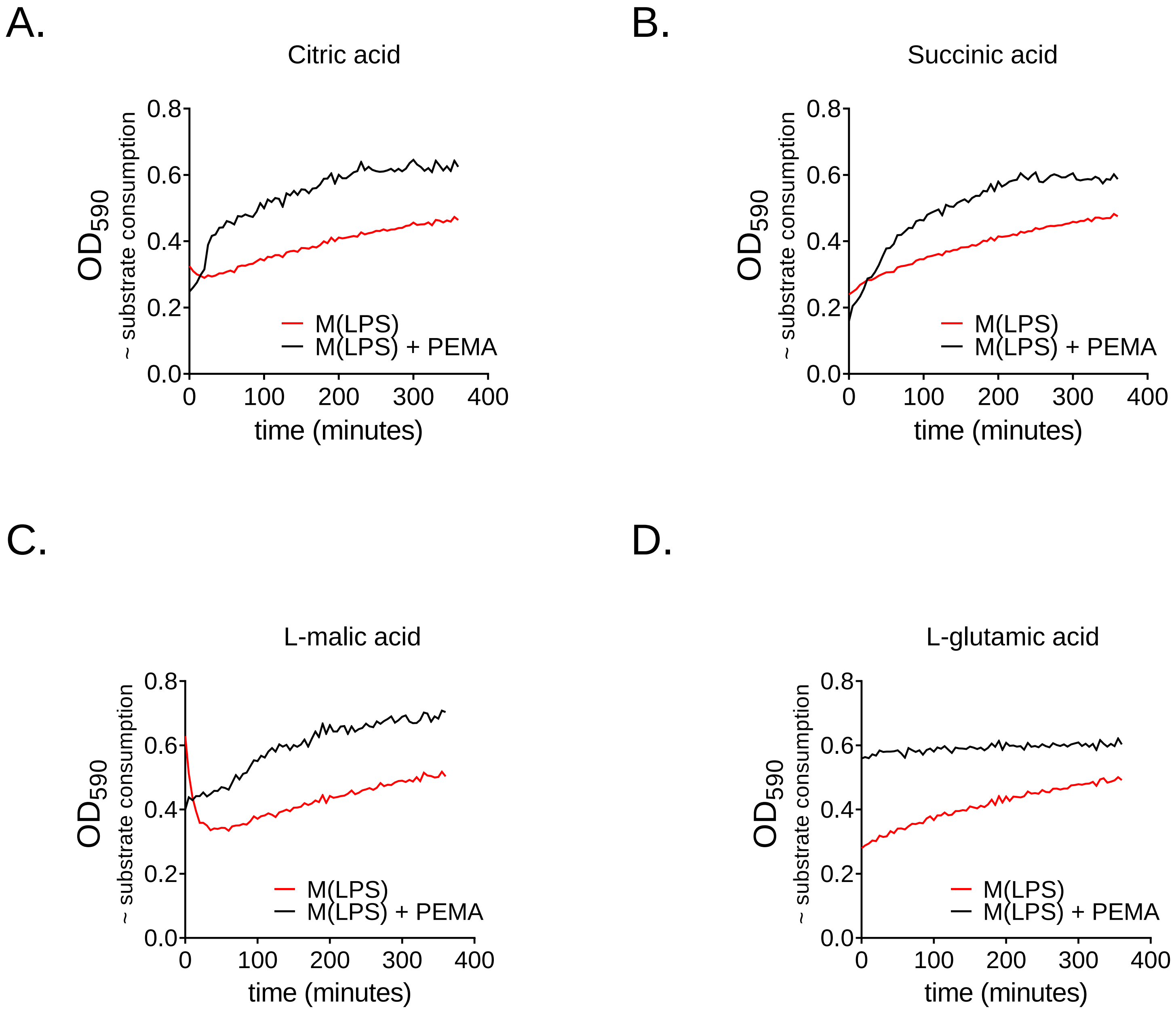

Figure XV. Isolated mitochondria TCA substrates consumption as measured by Mitoplate S assay. Time course monitored substrate consumption measured at 37°C of citric acid (**A.),** succinic acid **(B.**), L-malic acid (**C.**), and L-glutamic acid (**D.**) PEMA = pemafibrate. Mitochondria are isolated from THP-1 differentiated cells treated with LPS for 2 hours with or without pemafibrate (100 nM). Pemafibrate group was pretreated with 100 nM for 2 hours before LPS stimulation, while DMSO at 4 ppm was used for vehicle control. TCA=tricarboxylic acid cycle.

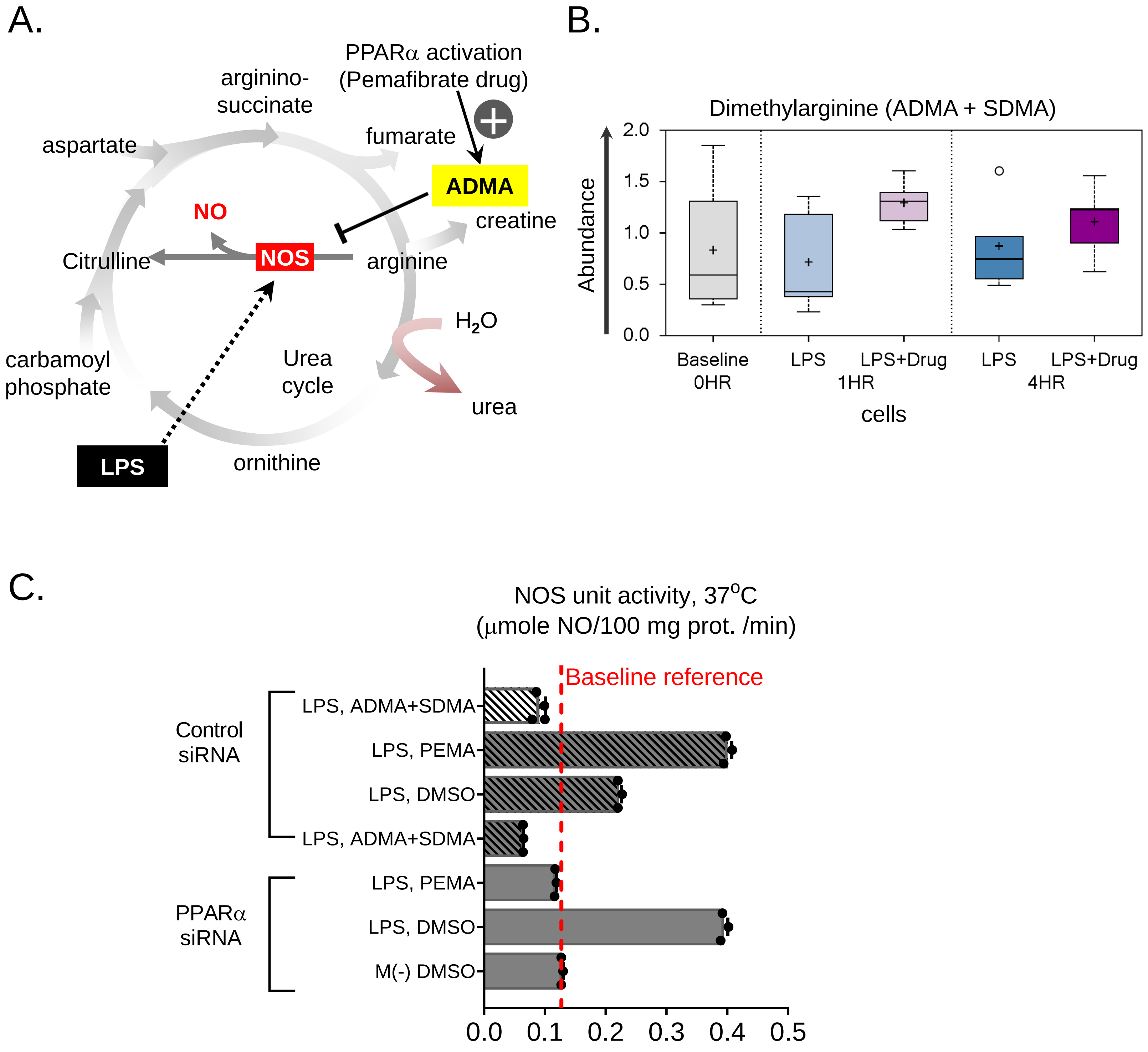

Figure XVI. NOS activity assay A. Urea cycle. ADMA is a known inhibitor of nitric oxide synthetase (NOS). **B.** PPAR αactivation (pemafibrate drug) increases ADMA and SDMA, as seen in whole-cell metabolomics (Metabolon, n=5 per sample condition). **C.** Nitric oxide synthetase (NOS) activity measured by NOS activity assay (Abcam) comparing various conditions of human primary monocyte-derived macrophages (n= 3 biological donors). Activity is measured as µmole NO produced per 100 mg protein cell lysate per minute at 37°C temperature. ADMA= asymmetric dimethylarginine, DMSO= dimethyl sulfoxide (vehicle control), SDMA = symmetric dimethylarginine. PPARα= Peroxisome Proliferator-Activated Receptor Alpha.

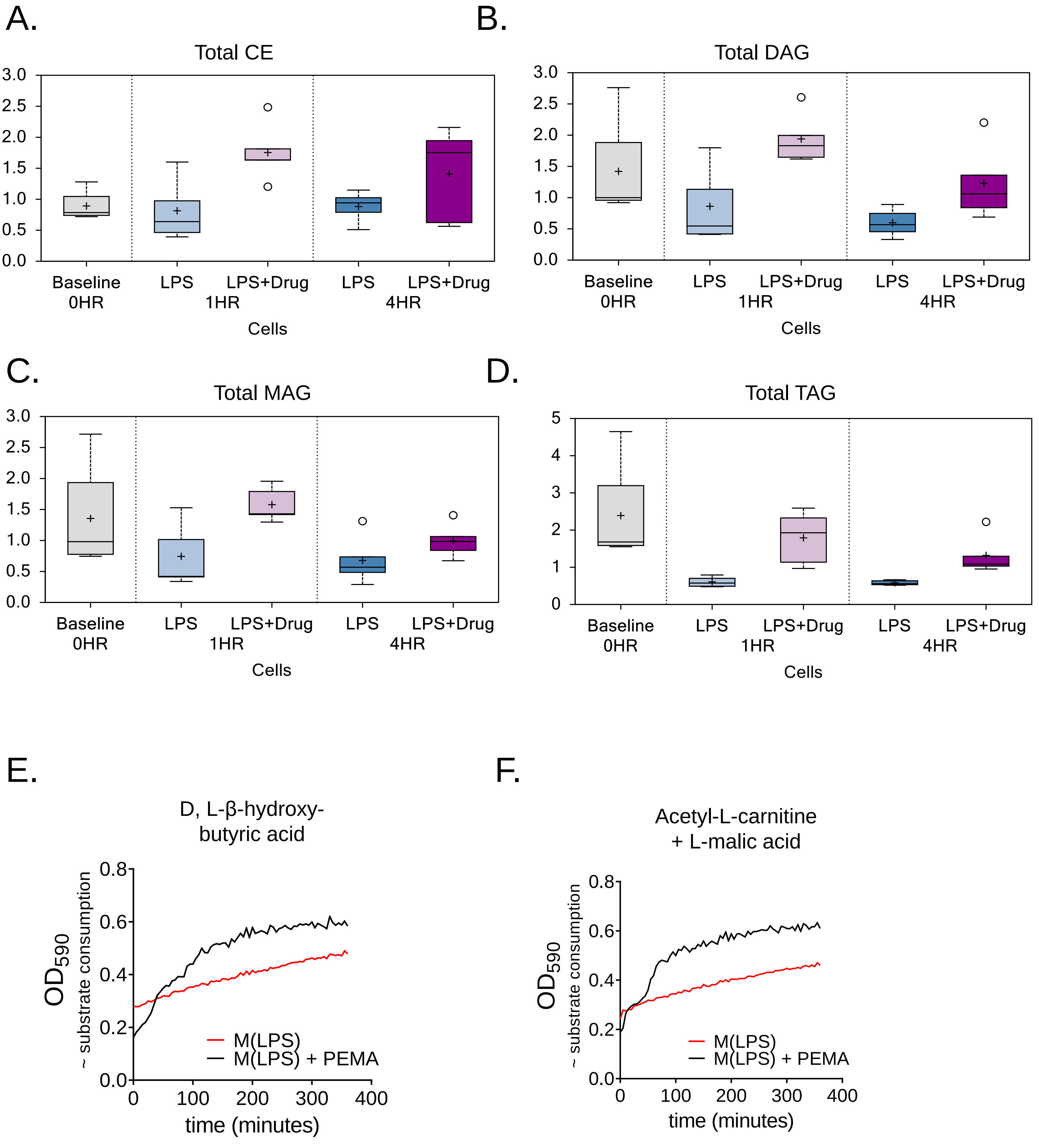

Figure XVII. Macrophage lipid loading, as assessed by lipidomics (Metabolon). A. CE= Cholesterol esters. **B.** DAG= diacylglycerol. **C.** MAG=monoacylglycerol. **D.** TAG= triacylglycerols. **E.-F.** THP-1 (M(LPS) vs M(LPS + PEMA)) isolated mitochondria tested for fatty acid oxidation substrate consumption assay (Mitoplate S, Biolog) for **E.** D, L-β-Hydroxy Butyric Acid, and **F.** Acetyl-L-Carnitine + L-Malic Acid. Drug/PEMA=pemafibrate.

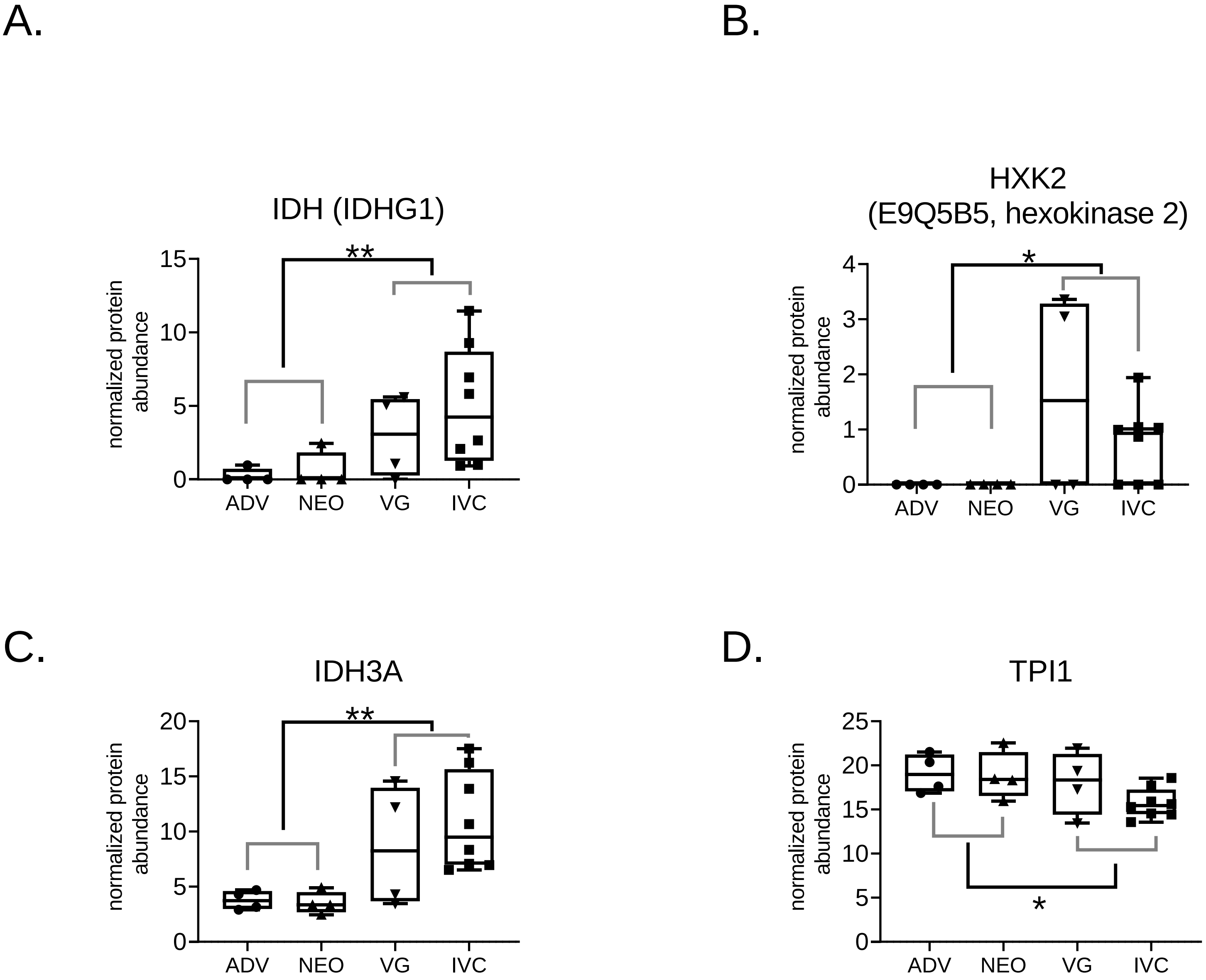

Figure XVIII. Relative abundances (normalized values) of proteins by LC/MS/MS. A. IDH, isocitrate dehydrogenase. **B.** HXK2, hexokinase 2. **C.** IDH3, isocitrate dehydrogenase isoform 3. **D.** TPI1, Triosephosphate isomerase 1. ADV= adventitia, (*Ldlr-/-*) IVC = inferior vena cava (wild type and *Ldlr-/-),* NEO= neointima (*Ldlr-/-*), VG= whole vein grafts (wild type). (*) p-value ≤ 0.05, (**) p-value ≤ 0.01

A.

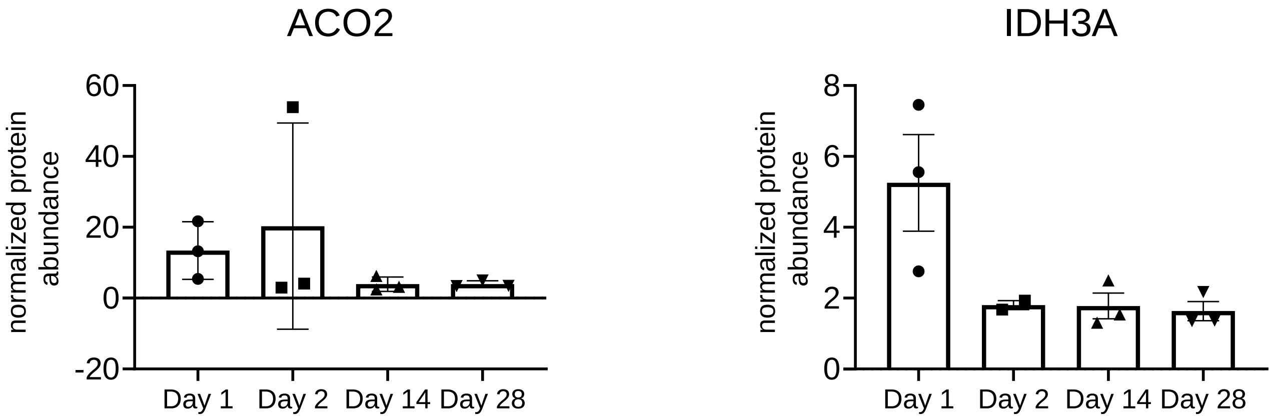

B.

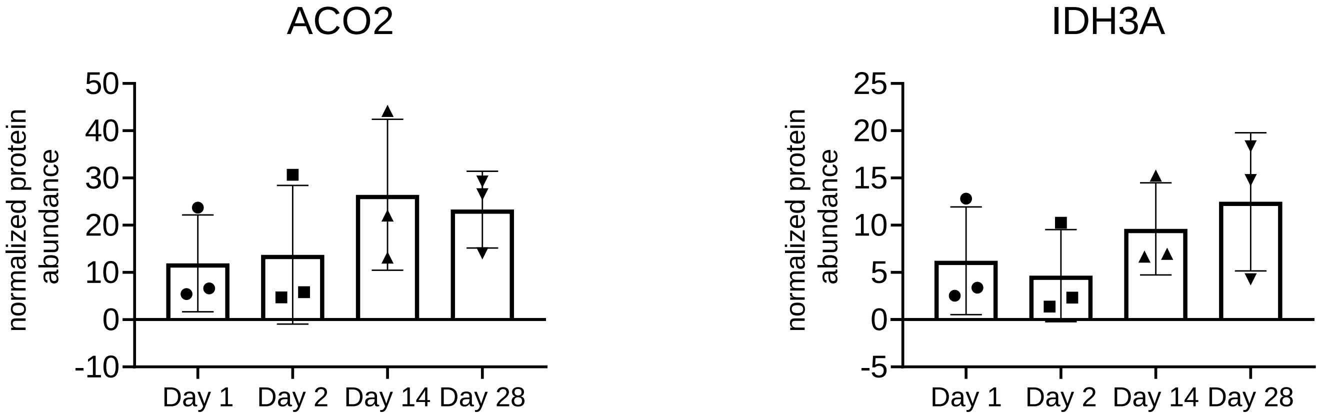

Figure XIX. Relative abundances (normalized values) of proteins by LC/MS/MS. A. Vein graft tissue in time course proteomics. **B.** IVC tissue samples in time course proteomics. ACO2, aconitase 2, IDH3A, isocitrate dehydrogenase-3A

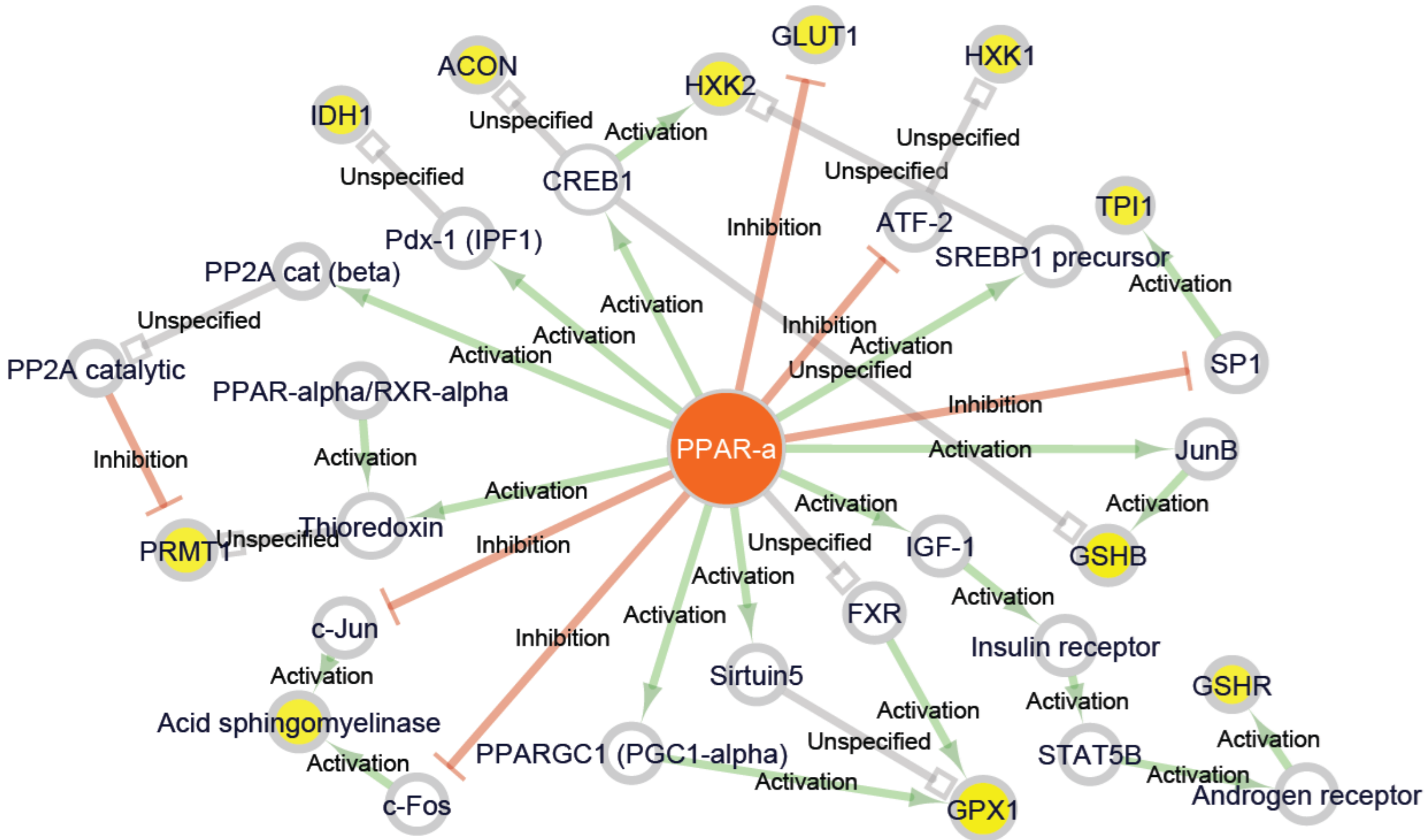

Figure XX. PPARα regulatory network. PPARα regulatory network for enzymes related to metabolomic changes (see Results). Edges = activation (green), inhibition (orange), and unspecified (gray).

Supplementary figure legends

Figure I. Mouse vein graft (VG) disease model. A. Plaque burden of wild type (WT) and *Ldlr-/-* mice, estimated as wall thickness. (*) $p < 0.05$, (**) $p < 0.01$. **B.** Short axis (SAx) view of the *Ldlr-/-* vein grafts, B-mode, 1-week vs. 3-weeks post-surgery. **C.** VG plaque B mode SAx plaque tracing, representative image (scalebar 500 µm), and counterpart H&E histologic morphometry of mid-cross-sectional area (scalebar 500 µm) and their (**D.)** correlation plot, n=11 mice. **E.-F.** 3D ultrasound volumes. Long axis view, LAx (scale bar 1 mm) **G.** Wild type vein graft 4-week time point ultrasound imaging in SAx B mode and 3D volume wireframe rendering. Representative images. Lumen and outer wall tracings in green and red, respectively **H.** *Ldlr*-/- vein graft 4-week time point ultrasound imaging. SAx B-mode and 3D volume wireframe rendering. Representative images. Scale=1 mm. **I.** FACS plot of CD45+ cells from the whole blood (RBCs lysed and residuals gated out) of fat-fed *Ldlr-/-* mice. Event percentages of Ganulo=granulocytes, Mono=monocytes, Lympho=lymphocytes shown. **J.** CD45+ cell count between non-fat fed (normal chow, NC) animals vs. fat-fed (high-fat diet, HFD), after four weeks, counts are normalized using CountBright™ absolute counting beads (BD Biosciences). **K.** Differential WBC count normalized per 10,000 CD45+ cells for Ly6C++ monocytes, CD19+ B cells and CD3+ T cells. (*) $p < 0.05$, (***) $p < 0.001$, (n.s.) $p \geq 0.05$.

Figure II. Mouse vein graft tissue layer and vein graft time course proteomics. A. Principal component analysis (PCA) of WT vein graft (WVG) and *Ldlr-/-* vein graft tissue layers (NEO and ADV) and corresponding IVCs (WT and *Ldlr-/-*) proteome. Multi-group comparisons filtered for p-value < 0.05 and false discovery rate, FDR < 0.05 yields 729 shared proteins among the tissue samples **B.** Similar analysis with PCA of *Ldlr-/-* vein graft tissue layers and their counterpart IVC only. **C.** Proteome profile of *Ldlr-/-* NEO, ADV and IVC in hierarchical clustering confirms what PCA clustering showed: IVC samples cluster separately from NEO and ADV as seen on the first

level of hierarchical clustering (*) of their proteomic profile. NEO and ADV may be more similar (expressing inflammation and thrombosis proteins) versus IVC in proteomic profile (cell respiration proteins) based on the multi-group comparison expressing. **D.** PCA of NEO and ADV without the effect of IVC samples showing the distribution of NEO and ADV samples after two-group comparison with FDR < 0.05. **E.** Proteomic profile of the same two-group comparison of NEO and ADV as an expression heatmap. All expression heatmaps are z-score normalized. All multi-group and two-group comparisons are made with a threshold of p-value < 0.05, FDR < 0.05. ADV= adventitial layer of the vein graft sample. IVC= inferior vena cava endogenous control. NEO= neointimal layer of the vein graft sample.

Figure III. Mouse vein graft time course proteomics. A. Short axis B-mode images of *Ldlr*-/- vein graft time course: 1 week, 2 weeks, 3 weeks, and 4 weeks after vein graft implantation. Lumen and outer wall tracings in white broken lines. **B.** Principal component analysis, PCA of all time course samples colored by tissue type: VG or IVC. **C.** PCA of all VG samples in the time course proteomics depicted per timepoint. Venn diagrams showing shared proteins between IVC Layer/static and IVC time course predominant proteins**,** NEO+ADV from static **(D.)** and whole vein graft (VG) time course predominant proteins **(E.),** IVC Layer/static and VG time course predominant proteins **(F.),** and NEO+ADV static and IVC time course predominant proteins **(G.)**

Figure IV. Network of enriched pathways. Static proteomics A. *Ldlr*-/- VG neointimal and adventitial predominant proteins pathways enrichment network. **B.** IVC and wild type VG predominant proteins pathways enrichment network. Node sizes are proportional to the number of proteins from the dataset that are present in the pathway node. Node color depicts the intensity of betweenness centrality for a specific pathway node.

Figure V. Network of enriched pathways. Time course proteomics. *Ldlr*-/- VG early timepoints predominant proteins pathways enrichment network. Node sizes are proportional to the number of proteins from the dataset present in the pathway node. Node color depicts the intensity of betweenness centrality for a specific pathway node.

Figure VI. Network of enriched pathways. Time course proteomic. *Ldlr*-/- VG late timepoints predominant proteins pathways enrichment network. Node sizes are proportional to the number of proteins from the dataset present in the pathway node. Node color depicts the intensity of betweenness centrality for a specific pathway node.

Figure VII. Measures of hub centrality for proteins within the top 3 most central pathway nodes of each of the proteomic networks. A. Scatterplot of betweenness centrality by closeness centrality, (**B.)** betweenness centrality by degree centrality, and **(C.)** closeness centrality by degree centrality. Red dots represent proteins found in the proteomic dataset from the top 1 biological pathway for the indicated sample condition group (e.g., NEO/ADV predominant proteins group). Blue dots are proteins from the top 2 biological pathways. Green dots are proteins from the top 3 pathway. **D.** PPARα lipid regulation pathway (MetaCore) in detail.

Figure VIII. A. Optimization of silencing of PPARα using siRNA lipid nanoparticle (C12-200, LNP) conjugates. siControl (n=5) and siPPARα (n=6) groups of *Ldlr-/- mice* were used. siRNA LNP were injected in the tail vein at a dose of 0.5 mg/kg/dose twice a week for three weeks. Animals were challenged with an intraperitoneal injection of 2% thioglycolate (w/v) for 48 hours (pain medications were appropriately given). Afterward, peritoneal lavage was done to harvest the peritoneal macrophages. **B.** Spleens were also harvested, and mononuclear cells were isolated by homogenizing the spleen and gradient centrifugation (Histopaque 1083). qPCR of peritoneal macrophages and spleen mononuclear preps were done to assess silencing. (*) $p < 0.05$, (n.s.) p 0.05. **C.** Bodyweight monitoring between Pemafibrate and Control group, n=11 mice per group comparison **D.** Food consumption monitoring between Pemafibrate and Control group, n=11 mice per group comparison (per cage average).

Figure IX. Immune cells and lipid profile assessment in vivo. **A.** Protease expression of MMP9 and MMP13 per CD68 staining is also significantly less in pemafibrate treated vein grafts. **(B.)** Non-fasting and **(C.)** fasting blood levels of triglycerides, cholesterol, and glucose between two groups: control vs. pemafibrate treated group (n=7-9 mice per group). Fasting was done for 17 hours. **D.** Differential WBC count between control and pemafibrate group, ELISA quantitation of blood circulating CXCL11, a chemokine secreted by monocytes. **E.** Corresponding normality plots. CTRL= control group, PEMA= pemafibrate group. **F.** Spearman correlation plot showed no statistical correlation between TG levels and circulating Ly6C++ monocyte fraction among the pemafibrate treated group. **G.** Fasting plasma concentrations of triglycerides, cholesterol, and glucose between control siRNA group (siCTRL, n=7) and PPARα silenced group (siPPARα, n=7).(*) $p < 0.05$, (**) $P < 0.01$, (***) $p < 0.001$, (n.s.) $p > 0.05$.

Figure X. *In vitro* validation: mRNA expression in primary macrophages. A. Bulk qPCR of proinflammatory and anti-inflammatory markers in mouse bone mar row derived macrophages (BMDM), with loss-of-function (PPARα siRNA) and gain-of-function (PPARα agonism by pemafibrate – 100nM). CXCL9, (**B.**) CXCL11, (**C.**) TLR2 and (**D.**) anti-inflammatory marker Ym1 in mouse BMDM primary macrophages, with loss-of-function (PPARα siRNA) and gain -of-function (PPARα agonism by pemafibrate – 100nM). **E.** Relative abundances (normalized values) of proteins by LC/MS/MS of vein graft tissue in time course proteomics and IVC tissue samples in time course proteomics. **F.** Workflow of single cell RNA sequencing. Macrophages are derived from monocytes isolated from buffy coats and then differentiated into macrophages *in vitro*. **G.** Similar workflow for single cell qPCR using Fluidigm C1 sorter and Bi oMark HD RT-PCR. Chil3 , chitinase-like 3 (Ym1). (*) $p <0.05$, (***) $p < 0.001$, (n.s.) $p > 0.05$. Green fluorescent viability dye is Calcein AM.

Figure XI. Single-cell RNA sequencing results in tSNE (viSNE) plots. A. tSNE plot M(-) vs. M(LPS), gated clusters of interest: M(-) cluster 1, M(LPS) cluster 1 **B.** Volcano plot of differential gene expression between M(LPS) cluster 1 and M(-) cluster 1 **C.** Single-cell expression of proinflammatory genes IL-1β, IL-6, and CXCL11. **D.** Single-cell gene expression of TCA related genes IDH1, IDH3, and ACADM. TCA= tricarboxylic acid cycle.

Figure XII. Single-cell qPCR results in tSNE (viSNE) plots. A. Scatterplot heatmap of cells expressing general macrophage markers (**A.**). Scale bar represents relative expression **B.** Pro-inflammatory markers CCL2, CXCL10, CXCL1 1, CXCL9, IL-1 β, IL-6, p 65-NFκB, and TNF α **C.** OXPHOS pathways markers (FAO, TCA, ETC). FAO= fatty acid oxidation. TCA= tricarboxylic acid cycle. ETC= electron transport chain. Each dot represents a cell in any of the three conditions being compared: (1) Baseline M(-) or non-stimulated human primary macrophages, monocyte-derived, (2) M(LPS) or LPS stimulated (~10 ng/mL) , and (3) M(LPS) pre- and co-treated with 100 nM pemafibrate (PPARα agonism).

Figure XIII. Seahorse metabolic assays. A. Glycolytic rate as extracellular acidification rate, ECAR, of BMDM cells, M(-) vs. M(LPS) in 140 minutes. LPS (10 ng/mL) or PBS injected at 20 minutes. **B.** Mitochondrial stress test measuring oxidative consumption rate (OCR) as a measure of mitochondrial respiration rate. Non-mitochondrial oxygen consumption, like ROS generation, is also measured, right panel. **C.** Glycolytic function, ECAR mpH/min. **D.** Glycolytic capacity, ECAR mpH/min. **E.** Glycolytic reserve (%). F. Non-glycolytic acidification, ECAR mpH/min 2-DG= 2-deoxyglucose, BMDM= bone marrow-derived macrophages. DMSO= dimethyl sulfoxide. ECAR= extracellular acidification rate, FCCP= Carbonyl cyanide-4-(trifluoromethoxy)phenylhydrazone, a mitochondrial uncoupler ionophore. LPS= lipopolysaccharide. Oligo/OLIGO= oligomycin Pema/PEMA= pemafibrate. PPARa= Peroxisome Proliferator-Activated Receptor Alpha. ROS = reactive oxygen species. Rot= rotenone.

Figure XIV. TCA substrates consumption as measured by Mitoplate S assay. A time-series measurement using the area-under-the-curve (AUC) of TCA substrate utilization in cell count normalized primary human macrophages (n=3 donors). Each assay plate measures only up to three conditions. The conditions tested were M(LPS) (LPS, siControl, DMSO), M(LPS) siControl + Pema, and M(LPS) siPPARα + Pema. Fumaric acid (**A.),** succinic acid **(B.**), L-glutamic acid (**C.**), and L-malic acid (**D.**) substrate utilization were measured in human primary macrophages, n = 3 donors. D, L-isocitric acid (**E.**), and α-ketobutyric acid (**F.**) substrate utilization is measured in THP-1 differentiated macrophage-like cells. PPARα= Peroxisome Proliferator-Activated Receptor Alpha; TCA=tricarboxylic acid cycle.

Figure XV. Isolated mitochondria TCA substrates consumption as measured by Mitoplate S assay. Time course monitored substrate consumption measured at 37°C of citric acid (**A.),** succinic acid **(B.**), L-malic acid (**C.**), and L-glutamic acid (**D.**) PEMA = pemafibrate. Mitochondria are isolated from THP-1 differentiated cells treated with LPS for 2 hours with or without pemafibrate (100 nM). Pemafibrate group was pretreated with 100 nM for 2 hours before LPS stimulation, while DMSO at 4 ppm was used for vehicle control. TCA=tricarboxylic acid cycle.

Figure XVI. NOS activity assay. A. Urea cycle. ADMA is a known inhibitor of nitric oxide synthetase (NOS). **B.** PPARα activation (pemafibrate drug) increasesADMA and SDMA, as seen in whole-cell metabolomics (Metabolon, n=5 per sample condition). **C.** Nitric oxide synthetase (NOS) activity measured by NOS activity assay (Abcam) comparing various conditions of human primary monocyte-derived macrophages (n= 3 biological donors). Activity is measured as μmole NO produced per 100 mg protein cell lysate per minute at $37^{O}C$ temperature. ADMA= asymmetric dimethylarginine, DMSO= dimethyl sulfoxide (vehicle control), SDMA = symmetric dimethylarginine. PP ARα= Peroxisome Proliferator-Activated Receptor Alpha.

Figure XVII. Macrophage lipid loading, as assessed by lipidomics (Metabolon). A. CE= Cholesterol esters. **B.** DAG= diacylglycerol. **C.** MAG=monoacylglycerol. **D.** TAG= triacylglycerols. Isolated mitochondria tested for fatty acid oxidation substrate consumption assay (Mitoplate S, Biolog) for **E.** D, L-β-Hydroxy Butyric Acid, and F. Acetyl-L-Carnitine + L-Malic Acid. Drug/PEMA=pemafibrate.

Figure XVIII. Relative abundances (normalized values) of proteins by LC/MS/MS.

A. IDH, isocitrate dehydrogenase. **B.** HXK2, hexokinase 2. **C.** IDH3, isocitrate dehydrogenase isoform 3. **D.** TPI1, Triosephosphate isomerase 1. ADV= adventitia, (Ldlr-/-) IV C = inferior vena cava (wi ld type and Ldlr-/-), NEO= neointima (Ldlr-/-), VG= whole vein grafts (wild type), (Ldlr-/-) IVC = inferior vena cava (wild type and Ldlr-/-), NEO= neointima (Ldlr-/-), VG= whole vein grafts (wild type). (*) p-value ≤ 0.05, (**) p-value ≤ 0.01

Figure XIX. Relative abundances (normalized values) of proteins by LC/MS/MS. A. Vein graft tissue in time course proteomics. **B.** IVC tiss ue samples in t me course proteomics. ACO2, aconitase 2, IDH3A, isocitrate dehydrogenase-3 A.

Figure XX. PPARα regulatory network. PPARα regulatory network for enzymes related to metabolomic changes (see Results). Edges = activation (green), inhibition (orange), and unspecified (gray).

SUPPLEMENTAL TABLES

Table I. NEO+ADV pathways enrichment and network nodes

	Pathways ID	log(p-values)	Betweenness Centrality
1	NETosis in SLE	5.08066	0.208696
2	Cytoskeleton remodeling_Hyaluronic acid/ CD44 signaling pathways	1.842241	0.208696
3	Glomerular injury in Lupus Nephritis	1.23277	0.170938
4	Immune response_Antigen presentation by MHC class I: cross presentation	2.06844	0.084016
5	Immune response_Alternative complement pathway	7.082915	0.046968
6	Alternative complement cascade disruption in age related macular degeneration	5.08066	0.046968
7	SLE genetic marker specific pathways in antigen presenting cells (APC)	1.303032	0.041945
8	Cell cycle_Initiation of mitosis	2.266081	0.039614
9	Apoptosis and survival_Granzyme A signaling	2.144118	0.039614
10	Development_Glucocorticoid receptor signaling	2.299642	0.037822
11	Oxidative stress_Role of ASK1 under oxidative stress	2.038342	0.037822
12	Regulation of degradation of deltaF508 CFTR in CF	1.92336	0.037822
13	Mechanisms of deltaF508 CFTR activation by S nitrosoglutathione	1.76853	0.037822
14	Transcription_Negative regulation of HIF1A function	1.493089	0.037822
15	Immune response_Antigen presentation by MHC class II	1.045323	0.031448
16	Complement pathway disruption in thrombotic microangiopathy	3.226067	0.02751
17	Apoptosis and survival_Endoplasmic reticulum stress response pathway	1.625435	0.026125
18	Immune response_Sublytic effects of membrane attack complex	1.469288	0.026125
19	Immune response_Antigen presentation by MHC class I, classical pathway	2.811634	0.023253
20	Transport_Clathrin coated vesicle cycle	1.434979	0.001932
21	Immune response_Lectin induced complement pathway	12.380698	0
22	Immune response_Classical complement pathway	12.136915	0
23	Signal transduction_mTORC2 upstream signaling	2.580044	0
24	G protein signaling_Regulation of CDC42 activity	2.063486	0
25	G protein signaling_RhoA regulation pathway	2.038342	0

26	G protein signaling_Regulation of RAC1 activity	1.990124	0
27	Cell adhesion_ECM remodeling	1.670195	0
28	Glucocorticoid induced elevation of intraocular pressure as glaucoma risk factor	1.543179	0
29	Myeloid derived suppressor cells and M2 macrophages in cancer	1.517698	0
30	Transport_Rab 9 regulation pathway	1.377786	0
31	GTP-XTP metabolism	1.249646	0
32	Immune response_Regulatory role of C1q in platelet activation	1.20621	0
33	G-protein signaling_RhoB regulation pathway	1.179076	0
34	Regulation of degradation of wtCFTR	1.085763	0
35	Cell cycle_Chromosome condensation in prometaphase	1.065451	0
36	Cytoskeleton remodeling_ESR1 action on cytoskeleton remodeling and cell migration	1.046144	0
37	Cell cycle_Sister chromatid cohesion	1.046144	0
38	wtCFTR traffic / Sorting endosome formation (normal)	1.046144	0
39	Proteolysis_Putative ubiquitin pathway	1.027751	0
40	CFTR folding and maturation (normal and CF)	1.01015	0
41	Proteolysis_Role of Parkin in the Ubiquitin-Proteasomal Pathway	1.01015	0
42	Apoptosis and survival_Role of nuclear PI3K in NGF/ TrkA signaling	0.993106	0
43	Cytoskeleton remodeling_Neurofilaments	0.993106	0
44	Immune response_Role of HMGB1 in dendritic cell maturation and migration	0.977159	0
45	DeltaF508-CFTR traffic / Sorting endosome formation in CF	0.961777	0
46	Translation_Regulation of translation initiation	0.961777	0
47	Development_Role of CNTF and LIF in regulation of oligodendrocyte development	0.946922	0
48	Transport_Aldosterone-mediated regulation of ENaC sodium transport	0.918653	0

Table II. IVC pathways enrichment and network nodes

	**Pathways ID**	**log(p-values)**	**Betweenness Centrality**
1	Transcription_Sirtuin6 regulation and functions	3.618704	0.224125
2	Regulation of lipid metabolism_PPAR regulation of lipid metabolism	2.531357	0.200913
3	Regulation of lipid metabolism_Insulin regulation of fatty acid metabolism	3.196543	0.07344
4	Leucine, isoleucine and valine metabolism.p.2	3.348334	0.047692
5	Lysine metabolism	3.348334	0.047692
6	Tryptophan metabolism	3.07443	0.047692
7	Tricarbonic acid cycle	3.79129	0.033866
8	Regulation of lipid metabolism_Regulation of lipid metabolism via LXR, NF-Y and SREBP	2.617443	0.028919
9	Mitochondrial unsaturated fatty acid beta-oxidation	2.472241	0.004566
10	Mitochondrial long chain fatty acid beta-oxidation	1.955068	0.004566
11	Immune response_Distinct metabolic pathways in naive and effector CD8+ T cells	2.050903	0.001142
12	Immune response_The effect of INDO on T cell metabolism	1.88773	0.001142
13	Nitrogen metabolism	2.663941	0
14	Serotonin - melatonin biosynthesis and metabolism	2.399463	0
15	Regulation of lipid metabolism_Regulation of fatty acid synthesis: NLTP and EHHADH	1.462937	0
16	Regulation of lipid metabolism_Regulation of acetyl-CoA carboxylase 2 activity in muscle	1.439854	0
17	Regulation of lipid metabolism_Regulation of fatty acid synthase activity in hepatocytes	1.439854	0
18	Cytoskeleton remodeling_Regulation of actin cytoskeleton by Rho GTPases	1.358427	0
19	Proteolysis_Putative ubiquitin pathway	1.358427	0
20	CFTR folding and maturation (normal and CF)	1.340369	0
21	Proteolysis_Role of Parkin in the Ubiquitin-Proteasomal Pathway	1.340369	0
22	Development_Glucocorticoid receptor signaling	1.323032	0
23	Cytoskeleton remodeling_Neurofilaments	1.323032	0
24	Mitochondrial ketone bodies biosynthesis and metabolism	1.290391	0
25	Ascorbate metabolism	1.274987	0
26	Histamine metabolism	1.26019	0
27	Adiponectin in pathogenesis of type 2 diabetes	1.26019	0

28	Apoptosis and survival_Role of IAP-proteins in apoptosis	1.232028	0
29	Putative pathways for stimulation of fat cell differentiation by Bisphenol A	1.218604	0
30	Oxidative stress_Role of ASK1 under oxidative stress	1.193074	0
31	Immune response_TLR ligands	1.193074	0
32	Propionate metabolism p.1	1.169027	0
33	Cholesterol and Sphingolipid transport / Transport from Golgi and ER to the apical membrane (normal and CF)	1.157578	0
34	Development_MAG-dependent inhibition of neurite outgrowth	1.157578	0
35	Development_Beta adrenergic receptors in brown adipocyte differentiation	1.157578	0
36	Regulation of degradation of deltaF508-CFTR in CF	1.135489	0
37	Regulation of metabolism_Bile acids regulation of glucose and lipid metabolism via FXR	1.114526	0
38	Transport_HDL-mediated reverse cholesterol transport	1.104467	0
39	L-Alanine and L-cysteine metabolism	1.085075	0
40	Regulation of metabolism_Triiodothyronine and Thyroxine signaling	1.075721	0
41	Regulation of lipid metabolism_Insulin signaling: generic cascades	1.057595	0
42	Mechanisms of deltaF508 CFTR activation by S-nitrosoglutathione	1.057595	0
43	Stimulation of TGF-beta signaling in lung cancer	1.048857	0
44	Cell adhesion_Integrin-mediated cell adhesion and migration	1.048857	0
45	Immune response_PD-1-induced metabolic changes in T cells	1.040291	0
46	Transport_The role of AVP in regulation of Aquaporin 2 and renal water reabsorption	1.031938	0
47	Development_Insulin, IGF-1 and TNF-alpha in brown adipocyte differentiation	1.015698	0
48	Gamma-aminobutyrate (GABA) biosynthesis and metabolism	1.0078	0
49	Immune response_HSP60 and HSP70/ TLR signaling pathway	1.000087	0
50	Immune response_Antigen presentation by MHC class I, classical pathway	1.000087	0
51	Airway smooth muscle contraction in asthma	0.98506	0
52	Pyruvate metabolism	0.96377	0
53	Mitochondrial dysfunction in neurodegenerative diseases	0.96377	0
54	Triacylglycerol metabolism p.1	0.956638	0
55	Signal transduction_mTORC1 downstream signaling	0.950007	0
56	Propionate metabolism p.2	0.936667	0

57	Butanoate metabolism	0.936667	0
58	Neuroprotective action of lithium	0.936667	0
59	Polyamine metabolism	0.930332	0
60	Development_Role of IL-8 in angiogenesis	0.923724	0
61	Signal transduction_mTORC2 upstream signaling	0.923724	0
62	Transcription_Negative regulation of HIF1A function	0.917574	0
63	Phenylalanine metabolism	0.917574	0
64	Signal transduction_mTORC2 downstream signaling	0.905529	0
65	Immune response_Sublytic effects of membrane attack complex	0.905529	0
66	Saturated fatty acid biosynthesis	0.899629	0
67	Tyrosine metabolism p.1 (dopamine)	0.888066	0
68	Blood coagulation_Platelet microparticle generation	0.882397	0
69	Catecholamine metabolism	0.860436	0
70	Immune response _CCR3 signaling in eosinophils	0.855115	0
71	L-Arginine metabolism	0.844664	0
72	Tyrosine metabolism p.2 (melanin)	0.824778	0
73	Transport_Intracellular cholesterol transport	0.824778	0
74	Peroxisomal branched chain fatty acid oxidation	0.824778	0
75	Muscle contraction_GPCRs in the regulation of smooth muscle tone	0.824778	0
76	Glycolysis and gluconeogenesis	0.806041	0
77	Immune response_Antigen presentation by MHC class I: cross-presentation	0.754487	0
78	Cytoskeleton remodeling_Cytoskeleton remodeling	0.742802	0

Table III. Early time point pathways enrichment and network nodes

	**Pathways ID**	**log(p-values)**	**Betweenness Centrality**
1	Cytoskeleton remodeling_TGF, WNT and cytoskeletal remodeling	3.516413	0.081219
2	Cytoskeleton remodeling_Cytoskeleton remodeling	2.476774	0.081219
3	Blood coagulation_Blood coagulation	3.695941	0.052854
4	Apoptosis and survival_Role of PKR in stress-induced apoptosis	1.970211	0.027484
5	IGF family signaling in colorectal cancer	1.8671	0.016209
6	Cell adhesion_PLAU signaling	3.695941	0.009866
7	Cell adhesion_ECM remodeling	3.29921	0.009866
8	Cell adhesion_Chemokines and adhesion	1.452102	0.009866
9	Immune response_Alternative complement pathway	1.970211	0.008985
10	Aberrant lipid trafficking and metabolism in age-related macular degeneration pathogenesis	1.8671	0.008985
11	TLRs-mediated IFN-alpha production by plasmacytoid dendritic cells in SLE	1.970211	0.001057
12	Cell adhesion_Plasmin signaling	2.320481	0
13	Cell adhesion_Cell-matrix glycoconjugates	2.250496	0
14	Nicotine signaling in dopaminergic neurons, Pt. 2 - axon terminal	2.145755	0
15	Role of IL-23/ T17 pathogenic axis in psoriasis	1.954677	0
16	Platelet activation as a result of endothelial dysfunction after stenting	1.924453	0
17	Myeloid-derived suppressor cells and M2 macrophages in cancer	1.813609	0
18	Transport_RAN regulation pathway	1.282246	0
19	DNA damage_Inhibition of telomerase activity and cellular senescence	1.237697	0
20	Translation_Role of Retinoic acid signaling in the initiation of translation	1.197568	0
21	Cytoskeleton remodeling_CDC42 in cellular processes	1.197568	0
22	Expression targets of Tissue factor signaling in cancer	1.197568	0
23	Cell adhesion_Endothelial cell contacts by non-junctional mechanisms	1.161025	0
24	CFTR folding and maturation (normal and CF)	1.161025	0
25	Translation_Regulation of translation initiation	1.111708	0
26	Cysteine-glutamate metabolism	1.081917	0
27	Development_G-CSF-induced myeloid differentiation	1.067831	0
28	NETosis in SLE	1.054187	0
29	Development_Transcription regulation of granulocyte development	1.041006	0
30	Protein folding and maturation_Bradykinin / Kallidin maturation	1.041006	0
31	Cell cycle_Spindle assembly and chromosome separation	1.02826	0
32	Development_Activation of astroglial cells proliferation by ACM3	1.02826	0
33	Immune response_Inflammasome in inflammatory response	1.00397	0
34	Development_Growth hormone signaling via STATs and PLC/IP3	1.00397	0
35	NALP3 inflammasome activation in age-related macular degeneration (AMD)	1.00397	0

36	Regulation of lipid metabolism_Regulation of lipid metabolism via LXR, NF-Y and SREBP	0.970211	0
37	Regulation of degradation of deltaF508-CFTR in CF	0.959398	0
38	Transcription_Receptor-mediated HIF regulation	0.959398	0
39	Translation _Regulation of EIF2 activity	0.959398	0
40	Development_VEGF-family signaling	0.938925	0
41	Translation_Insulin regulation of translation	0.929224	0
42	Protein folding and maturation_Angiotensin system maturation \ Human version	0.919374	0
43	Immune response_IL-13 signaling via JAK-STAT	0.910095	0
44	Role of alpha-6/beta-4 integrins in carcinoma progression	0.90101	0
45	Mechanisms of deltaF508 CFTR activation by S-nitrosoglutathione	0.883392	0
46	Protein folding and maturation_Angiotensin system maturation \ Rodent version	0.874844	0
47	Tissue Factor signaling in cancer via PAR1 and PAR2	0.866461	0
48	Cytoskeleton remodeling_Integrin outside-in signaling	0.866461	0
49	Development_TGF-beta receptor signaling	0.858237	0

Table IV. Late time point pathways enrichment and network nodes

	Pathways ID	log(p-values)	Betweenness Centrality
1	Development_Role of proteases in hematopoietic stem cell mobilization	2.926282	0.093213
2	Role of tumor microenvironment in plexiform neurofibroma formation in neurofibromatosis type 1	2.257353	0.093213
3	Cell adhesion_ECM remodeling	1.998699	0.036727
4	Cell adhesion_Cell-matrix glycoconjugates	2.279427	0.024914
5	Transcription_P53 signaling pathway	2.257353	0.024914
6	Transport_Clathrin-coated vesicle cycle	2.971022	0.000859
7	Immune response_Antiviral actions of Interferons	2.014618	0
8	Transport_Macropinocytosis	1.46916	0
9	Intercellular relations in asthma (general schema)	1.346595	0
10	DNA damage_Role of SUMO in p53 regulation	1.320845	0
11	Regulation of lipid metabolism_Regulation of acetyl-CoA carboxylase 1 activity	1.296623	0
12	Schema: Initiation of T cell recruitment in allergic contact dermatitis	1.273762	0
13	wtCFTR and deltaF508-CFTR traffic / Clathrin coated vesicles formation (normal and CF)	1.252045	0
14	Development_Role of HGF in hematopoietic stem cell mobilization	1.231436	0
15	Multiple sclerosis (general schema)	1.231436	0
16	wtCFTR traffic / Sorting endosome formation (normal)	1.211832	0
17	Immune response_MIF-mediated glucocorticoid regulation	1.193142	0
18	Resolution of inflammation in healing myocardial infarction	1.15814	0
19	Development_Cross-talk between VEGF and Angiopoietin 1 signaling pathways	1.141703	0
20	DeltaF508-CFTR traffic / Sorting endosome formation in CF	1.125924	0
21	Proteolysis_Putative SUMO-1 pathway	1.096042	0
22	Adiponectin in pathogenesis of type 2 diabetes	1.096042	0
23	Regulation of lipid metabolism_RXR-dependent regulation of lipid metabolism via PPAR, RAR and VDR	1.081917	0
24	Vitamin B6 metabolism	1.081917	0
25	Development_Inhibition of angiogenesis by PEDF	1.068288	0
26	Cell cycle_Spindle assembly and chromosome separation	1.042345	0
27	p53 signaling in Prostate Cancer	1.042345	0
28	Apoptosis and survival_Granzyme B signaling	1.042345	0
29	CCR4-dependent immune cell chemotaxis in asthma and atopic dermatitis	1.029932	0
30	Cell adhesion_Alpha-4 integrins in cell migration and adhesion	1.029932	0
31	Chemotaxis_CCR4-induced chemotaxis of immune cells	1.029932	0
32	Signal transduction_Erk Interactions: Inhibition of Erk	1.029932	0
33	Mechanism of action of CCR4 antagonists in asthma and atopic dermatitis (Variant 1)	1.029932	0
34	Apoptosis and survival_Caspase cascade	1.029932	0
35	Development_TGF-beta-dependent induction of EMT via SMADs	1.017955	0

36	Role of growth factor receptors transactivation by Hyaluronic acid / CD44 signaling in tumor progression	1.017955	0
37	Role of red blood cell adhesion to endothelium in vaso-occlusion in Sickle cell disease	0.994819	0
38	Immune response_Role of integrins in NK cells cytotoxicity	0.984221	0
39	Substance P-mediated inflammation and pain in Sickle cell disease	0.984221	0
40	Regulation of degradation of deltaF508-CFTR in CF	0.973467	0
41	Transcription_Sin3 and NuRD in transcription regulation	0.952725	0
42	Transcription_Role of Akt in hypoxia induced HIF1 activation	0.952725	0
43	Apoptosis and survival_Lymphotoxin-beta receptor signaling	0.943095	0
44	Regulation of lipid metabolism_PPAR regulation of lipid metabolism	0.943095	0
45	Chemotaxis_CCL16-, CCL20-, CXCL16- and CCL25-mediated cell migration	0.933301	0
46	Regulation of metabolism_Role of Adiponectin in regulation of metabolism	0.933301	0
47	N-Glycan biosynthesis p2	0.933301	0
48	Role of cell adhesion in vaso-occlusion in Sickle cell disease	0.933301	0
49	Development_VEGF signaling and activation	0.933301	0
50	Apoptosis and survival_FAS signaling cascades	0.924088	0
51	Influence of smoking on activation of EGFR signaling in lung cancer cells	0.924088	0
52	Development_G-protein-mediated regulation of MAPK-ERK signaling	0.905878	0
53	Immune response_MIF-induced cell adhesion, migration and angiogenesis	0.905878	0
54	Transcription_Androgen Receptor nuclear signaling	0.905878	0
55	Development_TGF-beta-dependent induction of EMT via MAPK	0.896881	0
56	Development_Leptin signaling via PI3K-dependent pathway	0.896881	0
57	Muscle contraction_Relaxin signaling pathway	0.888401	0
58	Globo-(isoglobo) series GSL Metabolism	0.888401	0
59	Stimulation of TGF-beta signaling in lung cancer	0.888401	0
60	Immune response_Histamine H1 receptor signaling in immune response	0.888401	0
61	Signal transduction_Soluble CXCL16 signaling	0.880085	0
62	Development_PEDF signaling	0.880085	0
63	Immune response_PD-1-induced metabolic changes in T cells	0.880085	0
64	Serotonin - melatonin biosynthesis and metabolism	0.880085	0
65	Immune response_IL-13 signaling via PI3K-ERK pathway	0.871924	0
66	Rheumatoid arthritis (general schema)	0.871924	0
67	Development_Beta-adrenergic receptors signaling via Cyclic AMP	0.856049	0
68	Immune response_HMGB1/RAGE signaling pathway	0.848324	0
69	Ganglioside Metabolism p1	0.841035	0
70	Immune response_CCL2 signaling	0.841035	0
71	Th17 cells in CF	0.841035	0
72	Membrane-bound ESR1: interaction with G-proteins signaling	0.841035	0
73	Nicotine metabolism in liver	0.826231	0
74	Immune response _IFN gamma signaling pathway	0.826231	0

75	Development_Regulation of cytoskeleton proteins in oligodendrocyte differentiation and myelination	0.812197	0
76	Immune response_IL-33 signaling pathway	0.812197	0
77	Development_Prolactin receptor signaling	0.812197	0
78	Immune response_IL-18 signaling	0.798603	0
79	Aberrant lipid trafficking and metabolism in age-related macular degeneration pathogenesis	0.798603	0
80	Development_Regulation of endothelial progenitor cell differentiation from adult stem cells	0.798603	0
81	Glucocorticoid-induced elevation of intraocular pressure as glaucoma risk factor	0.785686	0
82	Immune response_IL-4-induced regulators of cell growth, survival, differentiation and metabolism	0.779369	0
83	Transcription_Sirtuin6 regulation and functions	0.772885	0
84	Immune response_IFN-alpha/beta signaling via JAK/STAT	0.772885	0
85	Development_Regulation of epithelial-to-mesenchymal transition (EMT)	0.772885	0
86	Renal tubulointerstitial injury in Lupus Nephritis	0.76675	0
87	Ovarian cancer (main signaling cascades)	0.76675	0
88	Dysregulation of germinal center response in SLE	0.76675	0
89	Role of Tissue factor-induced Thrombin signaling in cancerogenesis	0.760701	0
90	Development_Growth factors in regulation of oligodendrocyte precursor cell proliferation	0.754981	0
91	Immune response_IL-4-responsive genes in type 2 immunity	0.737549	0
92	Glutathione metabolism	0.737549	0
93	Development_EGFR signaling pathway	0.732125	0
94	Tyrosine metabolism p.1 (dopamine)	0.732125	0
95	Cell adhesion_Integrin inside-out signaling in T cells	0.715795	0
96	Chemotaxis_Leukocyte chemotaxis	0.715795	0
97	Nociception_Nociceptin receptor signaling	0.705313	0
98	Immune response_IL-5 signaling via PI3K, MAPK and NF-kB	0.705313	0
99	Immune response_IFN-alpha/beta signaling via MAPKs	0.700275	0
100	Peroxisomal branched chain fatty acid oxidation	0.671213	0
101	Mitochondrial long chain fatty acid beta-oxidation	0.671213	0
102	Glomerular injury in Lupus Nephritis	0.631713	0
103	Aminoacyl-tRNA biosynthesis in cytoplasm	0.607831	0
104	Cell adhesion_Chemokines and adhesion	0.600153	0
105	Neurogenesis_NGF/ TrkA MAPK-mediated signaling	0.581865	0
106	Immune response_B cell antigen receptor (BCR) pathway	0.564633	0
107	Immune response_Antigen presentation by MHC class II	0.538652	0
108	NAD metabolism	0.53551	0
109	ATP/ITP metabolism	0.520569	0

Table V. Late time point pathways enrichment and network nodes

Network Dataset		Pathway	log_ pval	Betweenness centrality	Central object	Gene Name
IVC enriched pathways	1	Transcription_Sirtuin6 regulation and functions	3.619	0.224125	Sirt6	*Sirt6*
	2	Regulation of lipid metabolism_PPAR regulation of lipid metabolism	2.531	0.200913	PPAR-a	*Ppara*
	3	Regulation of lipid metabolism_Insulin regulation of fatty acid metabolism	3.197	0.07344	SREBP1	*Srebp1*
NEO+ADV enriched pathways	1	Immune response_TLR ligands	2.038	0.344529	TLR2	*Tlr2*
	2	LRRK2 in neurons in Parkinson's disease	2.063	0.286957	LRRK2	*Lrrk2*
	3	NETosis in SLE	5.081	0.208696	DNA material	*n/a*
Early timepoint enriched pathways	1	Cytoskeleton remodeling_TGF, WNT and cytoskeletal remodeling	3.516	0.081219	Rictor	*Rictor*
	2	Cytoskeleton remodeling_Cytoskeleton remodeling	2.477	0.081219	PAK	*Pak*
	3	Blood coagulation_Blood coagulation	3.696	0.052854	Thrombin	*F2*
Late timepoint enriched pathways	1	Development_Role of proteases in hematopoietic stem cell mobilization	2.926	0.093213	SDF-1	*Sdf1*
	2	Role of tumor microenvironment in plexiform neurofibroma formation in neurofibromatosis type 1	2.257	0.093213	c-kit	*Kit*
	3	Cell adhesion_ECM remodeling	1.999	0.036727	MMP-13	*Mmp13*
